# Association of body-shape phenotypes with imaging measures of body composition in the UK Biobank cohort: relevance to colon cancer risk

**DOI:** 10.1186/s12885-021-08820-6

**Published:** 2021-10-15

**Authors:** Sofia Christakoudi, Konstantinos K. Tsilidis, Evangelos Evangelou, Elio Riboli

**Affiliations:** 1 Department of Epidemiology and Biostatistics, School of Public Health, Imperial College London, St Mary’s Campus, Norfolk place, London, W2 1PG UK; 2 MRC Centre for Transplantation, King’s College London, Great Maze Pond, London, SE1 9RT UK; 3 Department of Hygiene and Epidemiology, University of Ioannina School of Medicine, Ioannina, Greece

## Abstract

**Background:**

Body mass index (BMI), waist and hip circumference are strongly correlated and do not reflect body composition. A Body Shape Index (ABSI) and Hip Index (HI) define waist and hip size among individuals with the same weight and height and would thus reflect body density. We examined differences in body composition between body-shape phenotypes defined with ABSI and HI and used this information to propose explanations for associations between body-shape phenotypes and colon cancer risk.

**Methods:**

We used data from the UK Biobank Resource for 15,520 men, 16,548 women with dual-emission X-ray absorptiometry (DXA) measurements; 3997 men, 4402 women with magnetic resonance imaging (MRI) measurements; 200,289 men, 230,326 women followed-up for colon cancer. We defined body-shape phenotypes as: large-ABSI-small-HI (“apple”), small-ABSI-large-HI (“pear”), small-ABSI-small-HI (“slim”), large-ABSI-large-HI (“wide”). We evaluated differences in body composition in linear models and associations with colon cancer risk in Cox proportional hazards models adjusted for confounders and explored heterogeneity by BMI.

**Results:**

Among individuals with the same height and weight, visceral adipose tissue (VAT) was lowest for “pear” and highest for “apple”, while abdominal subcutaneous adipose tissue (ASAT) was lowest for “slim” and highest for “wide” phenotype. In the gynoid region, differences between “apple” and “pear” phenotypes were accounted for mainly by fat mass in women but by lean mass in men. In men, lean mass was inversely associated with waist size, while the pattern of gynoid fat resembled ASAT in women. Lean and fat mass were higher for higher BMI, but not hand grip strength. Compared to normal weight “pear”, the risk of colon cancer in men (1029 cases) was higher for “apple” phenotype for normal weight (hazard ratio HR = 1.77; 95% confidence interval: 1.16–2.69) and comparably for overweight and obese, higher for “wide” phenotype for overweight (HR = 1.60; 1.14–2.24) and comparably for obese, but higher for “slim” phenotype only for obese (HR = 1.98; 1.35–2.88). Associations with colon cancer risk in women (889 cases) were weaker.

**Conclusions:**

ABSI-by-HI body-shape phenotypes provide information for body composition. Colon cancer risk in men appears related to ASAT quantity for “slim” and “wide” but to factors determining VAT accumulation for “apple” phenotype.

**Supplementary Information:**

The online version contains supplementary material available at 10.1186/s12885-021-08820-6.

## Background

Excess body weight, as reflected in body mass index (BMI), is accepted as a risk factor for cardiovascular diseases and several cancers [1], but BMI cannot distinguish the contribution of fat and lean mass. Abdominal size is associated positively with the metabolic complications of obesity, while gluteofemoral size is associated inversely [2], but waist (WC) and hip circumference (HC) are correlated strongly with each other and with BMI and cannot distinguish subcutaneous from visceral fat or lean from fat mass [3]. Although A Body Shape Index (ABSI) and Hip Index (HI) are also anthropometric indices, they are independent of weight and height by design and thus complement BMI [4, 5]. In analogy to BMI, which compares body mass among individuals with the same height, ABSI and HI, which are related to body volume, compare the transversal body dimensions, waist and hip circumference, among individuals with the same weight and height. Positive associations with ABSI and inverse with HI have been reported for mortality, cardio-metabolic risk factors and various cancers, including several cancers which are not considered obesity related [3, 4, 6, 7].

We hypothesised that body-shape phenotypes defined with ABSI and HI would provide information for body composition as follows. Individuals with small-ABSI-small-HI (“slim”) would have the smallest volume for a given weight and height and would thus comprise body components with higher density, i.e. a larger proportion of lean mass. Individuals with large-ABSI-large-HI (“wide”) would have the largest volume for a given weight and height and would thus comprise body components with lower density, i.e. a larger proportion of fat mass. Hence, there would be a density gradient between individuals with concordant ABSI and HI (“wide” vs “slim”). As the opposing metabolic effects of waist and hip size are mainly related to visceral fat [2], individuals with large-ABSI-small-HI (“apple”) would have the largest visceral fat depot for a given weight and height, while individuals with small-ABSI-large-HI (“pear”) would have the smallest visceral fat depot. Hence, there would be a visceral fat gradient between individuals with discordant ABSI and HI (“apple” vs “pear”).

Using high-quality body-composition measurements from dual-emission X-ray absorptiometry (DXA) and magnetic resonance imaging (MRI) scans available for a subset of UK Biobank participants, we examined the body-composition profile of body-shape phenotypes defined with ABSI and HI and explored heterogeneity by BMI. To illustrate the relevance of this information to cancer epidemiology, we examined in the complete cohort the association between body-shape phenotypes and the risk of development of colon cancer, as the most common obesity-related cancer relevant to both men and women, for which we have already reported for UK Biobank positive associations with ABSI and BMI and, in men, an inverse association with HI [7].

## Methods

### Study population

UK Biobank comprises half a million participants from the general population in the United Kingdom (UK), with age at enrolment 40 to 70 years, who were recruited between 2006 and 2010 and have been followed-up prospectively [8, 9]. In accordance with our previous studies in UK Biobank [7, 10], we restricted the study dataset to participants with self-reported white ancestry. To examine associations with body composition, we retained 32,068 participants with DXA measurement and 8399 with MRI measurements, excluding participants with missing imaging measurements, with anthropometric measurements which were extreme or missing at the imaging visit, with a mismatch between genetically determined and self-reported sex, younger than 45 or older than 75 at the imaging visit (to match the 30 years age window at enrolment and to minimise the influence of sarcopenia in the elderly), with prevalent cancer at the imaging visit, or with incident cancer or death within the first two years after the imaging visit (to minimise the influence of cancer cachexia). To examine associations with cancer risk, we excluded 71,873 participants with anthropometric measurements which were extreme or missing at enrolment, with a mismatch between genetically-determined and self-reported sex, younger than 40 or older than 70 years at enrolment, or with prevalent cancer at enrolment and pregnant women (Supplementary Fig. S1).

### Cancer ascertainment

Cancer cases in UK Biobank are ascertained based on cancer registry linkage. The outcome of interest was first primary colon cancer diagnosed after enrolment, defined as in our previous publication with code C18 from the 10th version of the International Classification of Diseases, and with behavioural code 3 or 5, excluding rare morphologies (histological codes 8240, 8241, 8243, 8245, 8246, 8472, 8743, 8936, 9680, 9699) [7]. We censored at the date of diagnosis participants with first colon cancer with behavioural codes 6, 9, or missing, or with colon cancer with rare morphology (as stated above), or with first primary cancer with other location. We censored follow-up at 31st March 2016 (up to which date the cancer registry information was complete) for all participants who had remained cancer-free, or censored follow-up at the date of death, if this was earlier.

### Anthropometric indices

Anthropometric measurements in UK biobank have been obtained by specifically trained technicians, at the natural indent or the umbilicus for waist circumference, and at the widest point for hip circumference [8]. To calculate ABSI for both sexes and HI for women, we used coefficients from the National Health and Nutrition Examination Survey (NHANES) [4, 5]. To calculate HI for men, we used simple-fraction coefficients based on UK Biobank data. HI calculated with coefficients from NHANES was uncorrelated with BMI in women but was inversely correlated with BMI in men from the UK Biobank cohort [7] and, similarly, in men from the European Prospective Investigation into Cancer and Nutrition (EPIC) cohort [3]. This suggests ethnic differences in HI, as 28% of NHANES participants had black ethnic background [5]. We have previously noted that the over-adjustment of hip circumference for weight and height observed in HI calculated with coefficients from NHANES can be corrected when ABSI, HI, BMI and height are examined in an additive model [7]. It was, however, important to avoid correlations with BMI when using HI as a free-standing index for cross-classification, as otherwise body-shape phenotypes would not be independent of body size. Calculating HI in men with coefficients from NHANES would have resulted in up to 40% differential misclassification with respect to BMI and HI, with normal weight men with small HI (“slim and “apple” phenotypes) being misclassified as large HI and obese men with large HI (“pear” and “wide” phenotypes) being misclassified as small HI (Supplementary Table S1). To calculate the waist-to-hip index (WHI), we used the waist-to-hip ratio (WHR) and simple-fraction coefficients based on UK Biobank data [10]:

$$ \mathrm{ABSI}=\mathrm{WC}\left(\mathrm{m}\mathrm{m}\right)\ast \mathrm{Weight}{\left(\mathrm{kg}\right)}^{\hbox{-} 2/3}\ast \mathrm{Height}{\left(\mathrm{m}\right)}^{5/6} $$

$$ {\mathrm{HI}}_{\mathrm{women}}=\mathrm{HC}\left(\mathrm{cm}\right)\ast \mathrm{Weight}{\left(\mathrm{kg}\right)}^{\hbox{-} 0.482}\ast \mathrm{Height}{\left(\mathrm{cm}\right)}^{0.310} $$

$$ {\mathrm{HI}}_{\mathrm{men}}=\mathrm{HC}\left(\mathrm{cm}\right)\ast \mathrm{Weight}{\left(\mathrm{kg}\right)}^{\hbox{-} 2/5}\ast \mathrm{Height}{\left(\mathrm{cm}\right)}^{1/5} $$

$$ \mathrm{WHI}=\mathrm{WHR}\ast \mathrm{Weight}{\left(\mathrm{kg}\right)}^{\hbox{-} 1/4}\ast \mathrm{Height}{\left(\mathrm{cm}\right)}^{1/2} $$

$$ \mathrm{BMI}=\mathrm{Weight}\left(\mathrm{kg}\right)\ast \mathrm{Height}{\left(\mathrm{m}\right)}^{\hbox{-} 2} $$

We dichotomised ABSI and HI using as cut-offs rounded numbers close to the sex-specific medians in the complete study dataset at enrolment: ≥73 for women and ≥ 80 for men for ABSI, ≥64 for women and ≥ 49 for men for HI. We categorised WHI in sex-specific quartiles and BMI in three groups according to the World Health Organisation cut-offs: normal weight (BMI ≥ 18.5 and < 25 kg/m^2^), overweight (BMI ≥ 25 and < 30 kg/m^2^) and obese (BMI ≥ 30 and < 45 kg/m^2^). Individuals with BMI < 18.5 and BMI ≥ 45 kg/m^2^ were excluded from the study, as they represented very small groups, which could have large leverage but could not be examined individually.

### Body-composition measurements

Body-composition measurements were obtained on average 8.7 years after enrolment for DXA and 6.7 years for MRI. We used DXA measurements for regional lean and fat mass and compared these with bioelectrical impedance analysis (BIA) measurements obtained at the imaging visit. For visceral (VAT) and abdominal subcutaneous adipose tissue (ASAT), we compared DXA mass and MRI volume measurements. As an indicator of muscle functionality, we used hand grip strength [11].

Whole-body DXA images were acquired with GE-Lunar iDXA scanner (GE Healthcare, Madison, Wisconsin, USA) and were analysed with GE enCORE software [12]. Regional fat and lean mass and VAT were available for a limited number of participants (2030 men and 2201 women). We used total regional mass and regional tissue fat percentage to calculate regional lean and fat mass for the complete DXA dataset (see details in Supplementary Methods). DXA regions were defined as follows: “arms” included the arms and shoulders areas; “trunk” included the neck, chest, abdominal and pelvic areas; “legs” included all remaining areas below the trunk; “android” overlapped the trunk region between the ribs and the pelvis; “gynoid” overlapped the legs and trunk regions, including the hips and upper thighs [13]. MRI images were acquired with a Siemens Aera 1.5 T scanner (Syngo MR D13) (Siemens, Erlangen, Germany), with a dual-echo Dixon Vibe protocol, and were analysed with AMRA profiler (Advanced MR Analytics, Linköping, Sweden) [14]. Measurements flagged with error codes by UK Biobank were considered missing. BIA measurements were obtained with Tanita BC-418MA Body Fat Analyser (Tanita Corp, Tokyo, Japan).

In analogy to allometric body-shape indices, we defined allometric body-composition indices. We scaled each body composition measurement with weight and height in a log-linear regression model:

$$ \log \left(\mathrm{Measurement}\right)\sim \upbeta \ast \log \left(\mathrm{Weight}\right)+\upgamma \ast \log \left(\mathrm{Height}\right) $$

and then calculated an allometric index according to the formula:

$$ \mathrm{Index}=\mathrm{Measurement}\ast {\mathrm{Weight}}^{\hbox{-} \upbeta}\ast {\mathrm{Height}}^{\hbox{-} \upgamma} $$

To explore heterogeneity by BMI, we calculated allometric body-composition indices with scaling only for height. The scaling regression coefficients are listed in Supplementary Table S2.

### Statistical analysis

We performed all analyses separately in men and women, because body shape and several cancers show substantial differences between sexes [15, 16].

To examine the association of body-shape phenotypes with body composition, we used body-composition indices as continuous variables, on the standardised scale of sex-specific z-scores (value minus mean, divided by standard deviation, SD; see means and SD per index in Supplementary Table S2). We calculated pairwise partial Pearson correlation coefficients between body-shape and body-composition indices with adjustment for the main factors potentially influencing body size, body shape and body composition listed below. We further used multivariable linear regression models to calculate SD differences in body-composition indices between body-shape phenotypes (“pear”-reference,” slim”,” wide”, “apple”) defined according to an ABSI-by-HI cross-classification, adjusting for self-reported weight change within the last year preceding the imaging visit, smoking status, alcohol consumption, physical activity and age at the imaging visit, as well as Townsend deprivation index (available only at enrolment), region of the imaging assessment centre (for the DXA dataset) and in women also a combined variable including menopausal status and, for post-menopausal women, use of hormonal replacement therapy evaluated at the imaging visit (the definition of covariates is described in detail in Supplementary Methods). For comparison, we additionally examined the body-composition profile of WHI quartiles. To examine heterogeneity by body size, we examined the body-composition profile of an BMI-by-ABSI-by-HI cross-classification, using body-composition indices scaled only for height. We tested heterogeneity by BMI with a likelihood ratio test, comparing a model including ABSI-by-HI and BMI as separate categorical variables with a model including a BMI-by-ABSI-by-HI cross-classification variable.

To examine the association of body-shape phenotypes with colon cancer risk, we estimated hazard ratios (HR) and 95% confidence intervals (CI) with delayed-entry Cox proportional hazards models, stratified by age at enrolment and region of the initial assessment centre. We used age as the underlying time scale, with the date of birth as origin, the date of attending an assessment centre at enrolment as the entry time, and the earliest of the date of diagnosis of the first incident colon cancer, or death, or date of censoring, as the exit time. All models were adjusted for height and potential confounders, as in the models examining body-composition indices as outcomes but evaluated at enrolment, and additionally for components of diet (consumption of vegetables and fresh fruit, fibre calculated according to [17], red meat, and processed meat), use of non-steroidal anti-inflammatory drugs, family history of cancer and, in women, age at the last live birth and use of oral contraceptives. Models for ABSI-by-HI were additionally adjusted for BMI. To explore possible reverse causality for cancer risk, we performed sensitivity analyses excluding participants with less than two years of follow-up. We additionally derived minimally adjusted HR estimates, with stratification by age and region and adjustment only for height and, for models with ABSI-by-HI, also for BMI, in order to explore the influence of covariates.

We used two-sided tests of statistical significance and considered *p* < 0.05 as a weaker evidence and *p* < 0.001 as a stronger evidence. Missing information for covariates was limited (Supplementary Table S3). To maximise sample size, we replaced missing values with the median category per sex. We used R version 4.0.5 for the linear regression models and STATA-13 for the Cox proportional hazards models [18, 19].

## Results

### Cohort characteristics

In total, 200,289 men and 230,326 women were included in the cancer risk dataset, 15,520 men and 16,548 women in the DXA body-composition dataset, and 3997 men and 4402 women in the MRI dataset. Mean BMI differed little between ABSI-by-HI body-shape phenotypes (up to 1.3 kg/m^2^) (Table 1), compared to a difference of up to 8.1 kg/m^2^ for body-shape phenotypes defined using WC-by-HC, which classified most participants with normal weight BMI as “slim” and most with obese BMI as “wide” phenotype (Supplementary Table S4). WHI was incremented between phenotypes in the order “pear”-“slim”-“wide”-“apple” (Table 1), with the first and fourth WHI quartiles overlapping substantially with “pear” and “apple” phenotypes but with all WHI quartiles contributing to “slim” and “wide” phenotypes (Supplementary Table S5).

**Table 1:** Demographic and anthropometric characteristics of study participants according to ABSI-by-HI body-shape phenotypes

	MEN						WOMEN					
	Overall	Pear	Slim	Wide	Apple	P	Overall	Pear	Slim	Wide	Apple	P
**Cancer risk dataset**												
Cohort size: n (%)	200,289	47,515 (23.7)	56,606 (28.3)	58,621 (29.3)	37,547 (18.7)		230,326	55,366 (24.0)	49,149 (21.3)	70,934 (30.8)	54,877 (23.8)	
Colon cancer cases: n (%)	1029	171 (16.6)	238 (23.1)	362 (35.2)	258 (25.1)		889	174 (19.6)	164 (18.4)	303 (34.1)	248 (27.9)	
Follow-up time (years)	6.9 (1.4)	7.0 (1.3)	6.9 (1.3)	6.9 (1.5)	6.8 (1.5)	< 0.001	7.0 (1.3)	7.1 (1.2)	7.0 (1.2)	7.0 (1.3)	6.9 (1.3)	< 0.001
Age at enrolment (years)	57.2 (8.1)	55.6 (8.4)	55.5 (8.1)	59.2 (7.6)	58.8 (7.5)	< 0.001	56.8 (8.0)	55.5 (8.1)	55.2 (8.0)	58.2 (7.7)	57.8 (7.7)	< 0.001
A Body Shape Index (ABSI)	79.8 (4.1)	77.1 (2.3)	76.3 (2.7)	83.3 (2.5)	83.0 (2.4)	< 0.001	73.8 (5.0)	69.7 (2.4)	69.3 (2.8)	77.4 (3.5)	77.3 (3.3)	< 0.001
Hip Index (HI)	49.1 (1.7)	50.2 (1.0)	47.6 (1.1)	50.4 (1.2)	47.9 (1.0)	< 0.001	64.2 (2.5)	65.9 (1.5)	62.1 (1.7)	66.0 (1.6)	62.2 (1.5)	< 0.001
Waist-to-Hip Index (WHI)	4.08 (0.22)	3.85 (0.14)	4.02 (0.16)	4.15 (0.15)	4.35 (0.16)	< 0.001	3.59 (0.27)	3.31 (0.14)	3.50 (0.16)	3.66 (0.18)	3.87 (0.20)	< 0.001
Body Mass Index (BMI) (kg/m^2^)	27.8 (4.0)	27.4 (4.2)	27.7 (3.7)	27.9 (4.4)	28.3 (3.9)	< 0.001	26.9 (4.8)	26.2 (4.8)	26.3 (4.4)	27.5 (5.2)	27.5 (4.4)	< 0.001
NW: ≥18.5 to < 25 kg/m^2^: n (%)	49,992 (25.0)	14,286 (30.1)	13,219 (23.4)	15,222 (26.0)	7265 (19.3)	< 0.001	92,272 (40.1)	26,765 (48.3)	22,218 (45.2)	26,258 (37.0)	17,031 (31.0)	< 0.001
OW: ≥25 to < 30 kg/m^2^: n (%)	99,591 (49.7)	22,667 (47.7)	29,911 (52.8)	27,650 (47.2)	19,363 (51.6)		85,748 (37.2)	18,461 (33.3)	18,380 (37.4)	24,930 (35.1)	23,977 (43.7)	
OB: ≥30 to < 45 kg/m^2^: n (%)	50,706 (25.3)	10,562 (22.2)	13,476 (23.8)	15,749 (26.9)	10,919 (29.1)		52,306 (22.7)	10,140 (18.3)	8551 (17.4)	19,746 (27.8)	13,869 (25.3)	
**DXA dataset**												
Cohort size: n (%)	15,520	2515 (16.2)	6951 (44.8)	3060 (19.7)	2994 (19.3)		16,548	3175 (19.2)	4408 (26.6)	4590 (27.7)	4375 (26.4)	
Time from enrolment (years)	8.7 (1.8)	8.4 (1.8)	8.6 (1.8)	8.8 (1.7)	9.2 (1.6)	< 0.001	8.7 (1.7)	8.4 (1.8)	8.6 (1.8)	8.9 (1.6)	8.8 (1.8)	< 0.001
Age at imaging (years)	63.9 (7.1)	63.1 (7.3)	62.6 (7.1)	66.1 (6.5)	65.4 (6.7)	< 0.001	62.9 (7.0)	62.3 (7.0)	61.5 (7.1)	64.3 (6.8)	63.4 (6.8)	< 0.001
A Body Shape Index (ABSI)	78.9 (4.2)	76.9 (2.4)	76.0 (2.8)	83.3 (2.6)	82.7 (2.3)	< 0.001	73.8 (5.2)	69.6 (2.6)	69.1 (2.8)	77.9 (3.9)	77.3 (3.3)	< 0.001
Hip Index (HI)	48.3 (1.9)	50.1 (0.9)	47.1 (1.3)	50.4 (1.3)	47.6 (1.1)	< 0.001	63.8 (2.6)	65.8 (1.5)	61.7 (1.7)	66.1 (1.6)	62.0 (1.6)	< 0.001
Waist-to-Hip Index (WHI)	4.09 (0.22)	3.85 (0.14)	4.04 (0.17)	4.14 (0.15)	4.36 (0.16)	< 0.001	3.62 (0.27)	3.31 (0.15)	3.51 (0.16)	3.69 (0.20)	3.90 (0.20)	< 0.001
Body Mass Index (BMI) (kg/m^2^)	27.1 (3.9)	27.0 (4.2)	27.0 (3.6)	27.2 (4.3)	27.4 (3.9)	< 0.001	26.2 (4.5)	25.9 (4.7)	25.5 (4.1)	26.6 (4.9)	26.5 (4.2)	< 0.001
NW: ≥18.5 to < 25 kg/m^2^: n (%)	4958 (31.9)	872 (34.7)	2206 (31.7)	1031 (33.7)	849 (28.4)	< 0.001	7759 (46.9)	1662 (52.3)	2311 (52.4)	2024 (44.1)	1762 (40.3)	< 0.001
OW: ≥25 to < 30 kg/m^2^: n (%)	7501 (48.3)	1163 (46.2)	3467 (49.9)	1369 (44.7)	1502 (50.2)		5824 (35.2)	956 (30.1)	1495 (33.9)	1549 (33.7)	1824 (41.7)	
OB: ≥30 to < 45 kg/m^2^: n (%)	3061 (19.7)	480 (19.1)	1278 (18.4)	660 (21.6)	643 (21.5)		2965 (17.9)	557 (17.5)	602 (13.7)	1017 (22.2)	789 (18.0)	
**MRI dataset**												
Cohort size: n (%)	3997	886 (22.2)	1857 (46.5)	780 (19.5)	474 (11.9)		4402	1077 (24.5)	1253 (28.5)	1007 (22.9)	1065 (24.2)	
Time from enrolment (years)	6.6 (1.0)	6.6 (1.1)	6.6 (1.0)	6.7 (1.0)	6.6 (1.0)	0.058	6.7 (1.0)	6.7 (1.0)	6.6 (1.0)	6.7 (1.0)	6.7 (1.1)	0.012
Age at imaging (years)	62.8 (7.4)	62.2 (7.5)	61.4 (7.5)	65.7 (6.5)	64.9 (6.3)	< 0.001	61.7 (7.2)	61.3 (7.1)	60.4 (7.4)	63.3 (6.9)	62.1 (7.0)	< 0.001
A Body Shape Index (ABSI)	78.1 (3.9)	76.8 (2.4)	75.8 (2.8)	82.6 (2.2)	82.2 (2.1)	< 0.001	72.9 (4.6)	69.5 (2.6)	69.5 (2.5)	76.7 (3.0)	76.7 (3.1)	< 0.001
Hip Index (HI)	48.6 (1.7)	50.1 (1.0)	47.4 (1.1)	50.4 (1.1)	47.8 (1.0)	< 0.001	63.8 (2.4)	65.9 (1.5)	61.9 (1.6)	65.8 (1.4)	62.1 (1.5)	< 0.001
Waist-to-Hip Index (WHI)	4.03 (0.2)	3.85 (0.14)	4.01 (0.16)	4.11 (0.12)	4.31 (0.14)	< 0.001	3.58 (0.26)	3.31 (0.15)	3.51 (0.15)	3.64 (0.16)	3.86 (0.18)	< 0.001
Body Mass Index (BMI) (kg/m^2^)	27.1 (3.8)	27.1 (4.0)	27.3 (3.6)	26.8 (4.1)	27.0 (3.6)	0.013	26.2 (4.5)	25.7 (4.6)	25.8 (4.2)	26.8 (5.0)	26.5 (4.0)	< 0.001
NW: ≥18.5 to < 25 kg/m^2^: n (%)	1235 (30.9)	297 (33.5)	503 (27.1)	289 (37.1)	146 (30.8)	< 0.001	2055 (46.7)	563 (52.3)	635 (50.7)	422 (41.9)	435 (40.8)	< 0.001
OW: ≥25 to < 30 kg/m^2^: n (%)	1975 (49.4)	405 (45.7)	986 (53.1)	341 (43.7)	243 (51.3)		1555 (35.3)	335 (31.1)	427 (34.1)	343 (34.1)	450 (42.3)	
OB: ≥30 to < 45 kg/m^2^: n (%)	787 (19.7)	184 (20.8)	368 (19.8)	150 (19.2)	85 (17.9)		792 (18.0)	179 (16.6)	191 (15.2)	242 (24.0)	180 (16.9)	

*ABSI* a body shape index (cut-offs: ≥80 for men; ≥73 for women); *Apple* large-ABSI-small-HI; *DXA* dual-emission X-ray absorptiometry; *HI* hip index (cut-offs: ≥49 for men; ≥64 for women); *MRI* magnetic resonance imaging; *n (%)* number of participants (percentage from the total per column or, for cohort size and cancer cases, percentage from total per sex); *NW* normal weight; *OB* obese; *OW* overweight; *P p*-values from one-way ANOVA (continuous variables) or chi-squared test (categorical variables) comparing the four body-shape phenotypes per sex; *Pear* small-ABSI-large-HI; *Slim* small-ABSI-small-HI; *Wide* large-ABSI-large-HI. Continuous variables are summarised with mean (standard deviation)

Participants with “apple” phenotype were more likely to be current or former regular smokers, with higher Townsend deprivation index, using non-steroidal anti-inflammatory drugs, consuming more red and processed meat but less fruit, vegetables and fibre, and less likely to be physically active, with women also being more likely to have children at an earlier age, compared to participants with “pear” phenotype (Supplementary Table S3). Participants with “wide” phenotype were the oldest and least physically active, with women also being less likely ever users of oral contraceptives or current HRT users. Participants with “apple” and “wide” phenotypes were also more likely to have a family history of cancer and to consume alcohol daily. Participants with “slim” phenotype were the most physically active.

In the imaging datasets, BMI, ABSI and HI were lower compared to the cancer risk dataset and participants were less likely to have gained weight during the last year preceding the visit, to be smokers, daily alcohol consumers, physically inactive or, in women, ever HRT users, compared to the cancer risk dataset (Table 1, Supplementary Table S3).

### Correlation between anthropometry and body composition

**Fig. 1:**
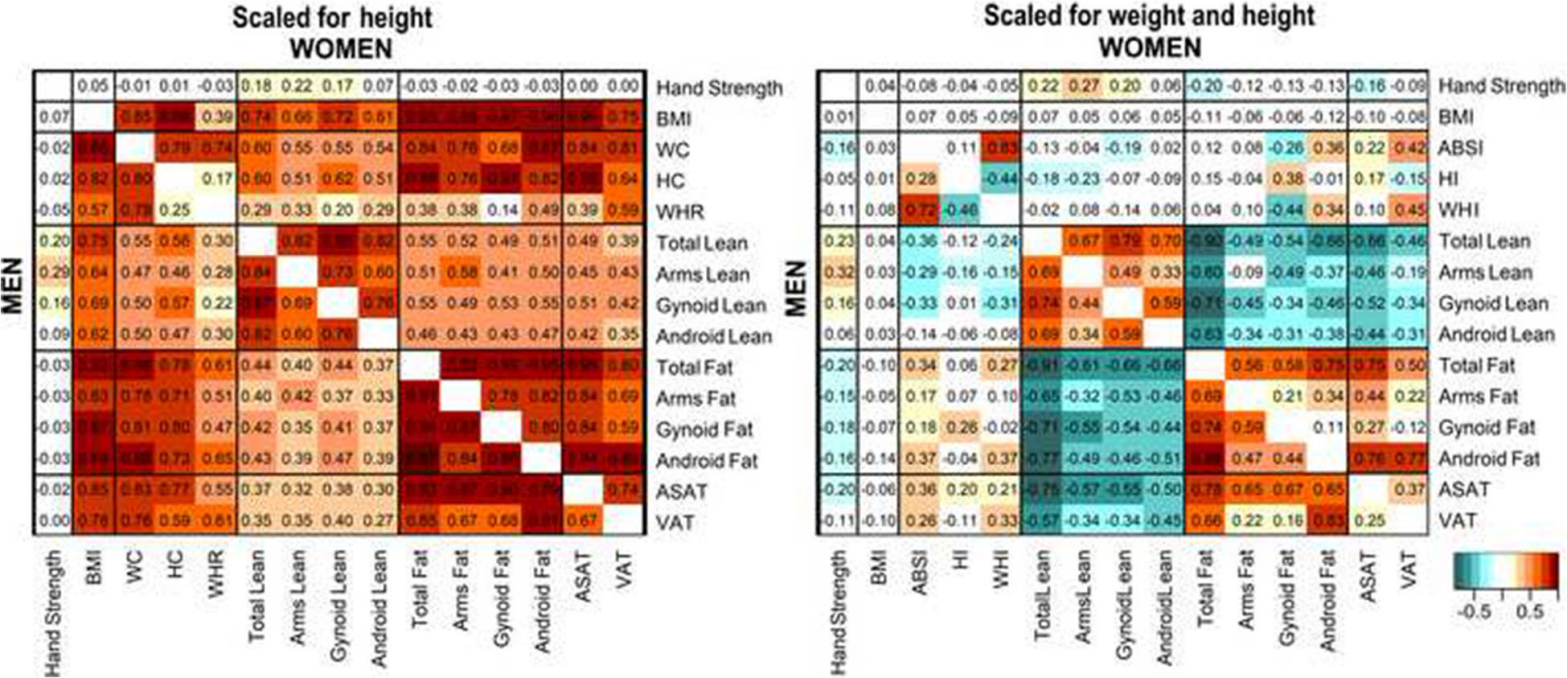
Correlation between anthropometry and body composition. ABSI – a body shape index; ASAT – abdominal subcutaneous adipose tissue; BMI – body mass index; HC – hip circumference; HI – hip index; VAT – visceral adipose tissue; WC – waist circumference; WHI – waist-to-hip index; WHR – waist-to-hip ratio. Men – bottom-left half of each panel. Women – top-right half of each panel. Cells – show partial Pearson correlation coefficients, with adjustment for age, weight change during the last year preceding the visit, smoking status, alcohol consumption, physical activity, Townsend deprivation index, region (except for VAT and ASAT) and, for women, menopausal status and use of hormonal replacement therapy (see definition of covariates in Supplementary Methods). Lean and fat mass correspond to dual-emission X-ray absorptiometry (DXA) measurements. VAT and ASAT correspond to magnetic resonance imaging (MRI) measurements. Anthropometry, hand grip strength and DXA measurements were obtained from the DXA dataset and MRI measurements from the overlap of the DXA and MRI datasets (see Supplementary Fig. S1 for the definition of datasets). Body-composition measurements were converted to allometric indices with scaling for height (left panel) or height and weight (right panel) (see scaling coefficients and formulas in Supplementary Table S2) and then to sex-specific z-scores (value minus mean, divided by the standard deviation). Complete correlation heatmaps, including all body-composition measurements, are shown in Supplementary Fig. S2 (for scaling with height) and Supplementary Fig. S3 (for scaling with height and weight)

Among participants with the same height, all body-shape and body-composition indices were positively correlated with each other and with BMI (Fig. 1, Supplementary Fig. S2). This included positive correlations between lean and fat mass in all regions and between VAT and gynoid fat mass, which are expected to be functionally different. Both WC and HC were positively correlated with lean and fat mass in the android and the gynoid regions.

The correlation patterns, however, differed considerably when participants were aligned by weight, in addition to height (Fig. 1, Supplementary Fig. S3). Lean mass was inversely correlated with fat mass in all regions. The correlations between VAT and gynoid fat mass were weak. In women, ABSI was correlated positively with android fat mass but not with android lean mass and was correlated inversely with gynoid lean and fat mass. In men, ABSI was correlated positively with fat mass (more strongly with android than with gynoid fat mass) and inversely with lean mass (more strongly with gynoid than with android lean mass). In both sexes, ABSI was correlated positively with VAT and ASAT, HI was correlated positively with gynoid fat mass and not with android fat mass overall, or with gynoid or android lean mass, but HI was correlated weakly positively with ASAT and inversely with VAT. Hand grip strength was weakly positively correlated with lean mass, most strongly for the arms.

### Body-composition profile of body-shape phenotypes

In the text below, we describe the patterns of lean and fat mass based on DXA and the patterns of VAT and ASAT based on MRI.

**Fig. 2:**
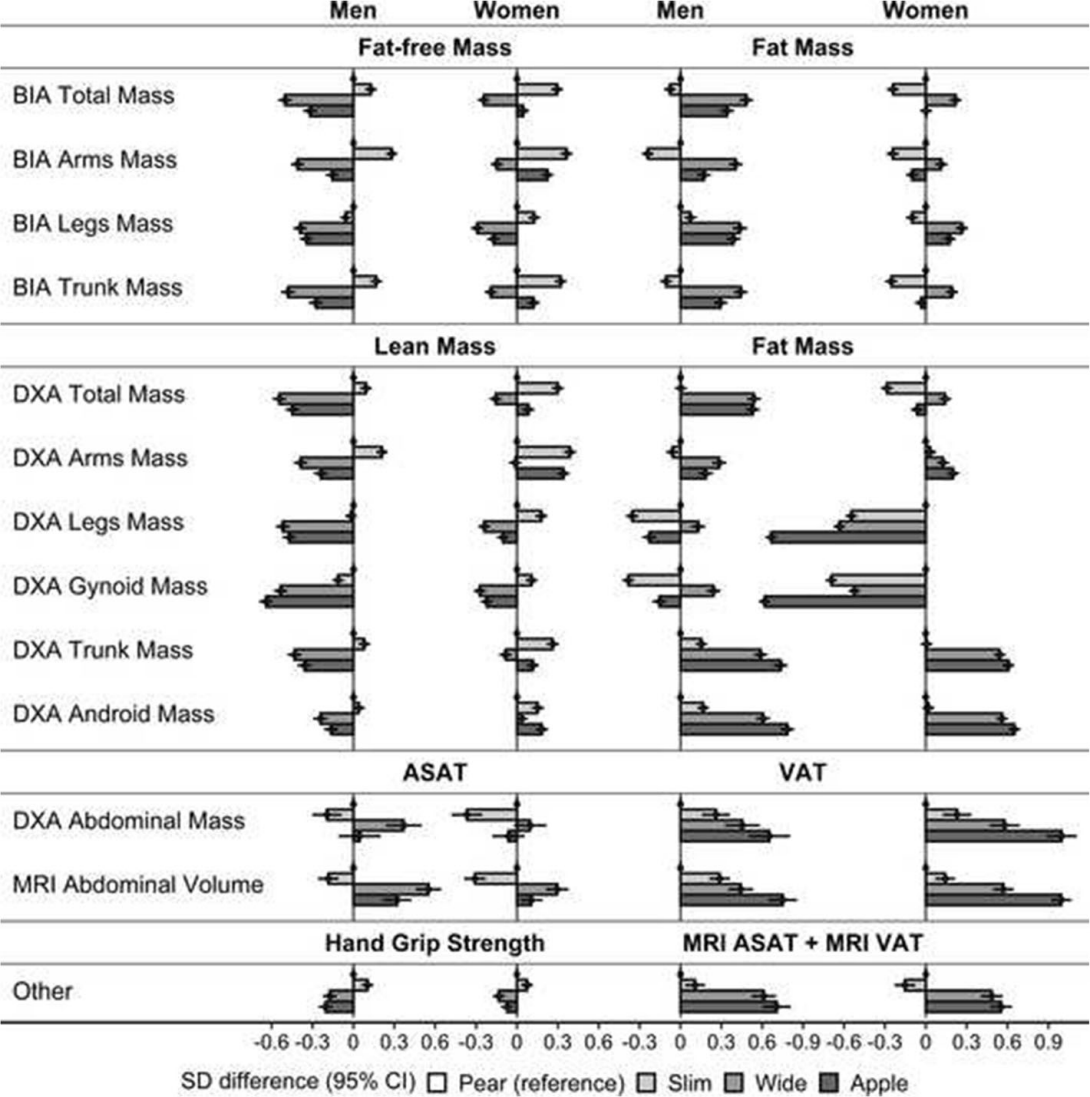
Body composition profiles of body-shape phenotypes. ABSI – a body shape index (cut-offs: ≥80 in men; ≥73 in women); Apple – large-ABSI-small-HI; ASAT – abdominal subcutaneous adipose tissue; BIA – bioelectrical impedance analysis measurements; CI – confidence interval; DXA – dual-emission X-ray absorptiometry measurements; HI – hip index (cut-offs: ≥49 in men; ≥64 in women); MRI – magnetic resonance imaging measurements; Pear – small-ABSI-large-HI; SD – standard deviation; Slim – small-ABSI-small-HI; VAT – visceral adipose tissue; Wide – large-ABSI-large-HI; SD difference (95% CI) – derived from linear regression models with adjustment for age, self-reported weight change within the year preceding the visit, smoking status, alcohol consumption, physical activity, Townsend deprivation index, region (except for VAT, ASAT and MRI) and, in women, menopausal status and use of hormonal replacement therapy (see definition of covariates in Supplementary Methods and numerical values in Supplementary Table S6). Body-composition measurements were converted to allometric indices with scaling for height and weight (see scaling coefficients in Supplementary Table S2) and then to sex-specific z-scores (value minus mean, divided by the standard deviation). Note that DXA lean mass does not include bone mass, which is included in BIA fat-free mass. See Supplementary Methods for the calculation of DXA lean and fat mass. The patterns in the total DXA dataset were consistent with the patterns in the restricted DXA VAT subset, which contained measurements for regional DXA lean and fat mass provided by UK Biobank (Supplementary Fig. S4, see Supplementary Fig. S1 for the definition of datasets). The adjustment for co-variates contributed only to a minor reduction in the SD differences (see Supplementary Fig. S5 for models adjusted only for age and region, except for a single region for VAT, ASAT and MRI)

As hypothesised, total lean and fat mass differed between phenotypes with concordant waist and hip size in both sexes (Fig. 2). In women, differences in total fat and lean mass were maximised between “slim” and “wide” phenotypes, with the highest total lean mass for “slim” and the highest total fat mass for “wide” phenotype, but arms and android lean mass were inversely associated with hip size (highest for “slim” and “apple” phenotypes). In men, total and regional lean mass were inversely associated with waist size (lowest for “wide” and “apple” phenotypes), while total fat mass was associated positively with waist size (highest for “wide” and “apple” phenotypes). In both sexes, android fat mass overall was associated positively with waist size, but VAT was lowest for “pear” and highest for “apple” phenotype, while ASAT was lowest for “slim” and highest for “wide” phenotype, as hypothesised. The pattern of gynoid fat mass, however, differed substantially between men and women. In women, gynoid fat mass was lowest for “apple” and highest for “pear” phenotype while, in men, was lowest for “slim” and highest for “wide” phenotype. Consequently, the gynoid regions of “apple” and “pear” phenotypes differed mainly with respect to fat mass in women but with respect to lean mass in men. Associations of hand grip strength with body-shape phenotypes resembled the pattern of arms lean mass in men but of gynoid lean mass in women.

The patterns of VAT and ASAT based on DXA and MRI were in agreement (Fig. 2). Associations of body-shape phenotypes with BIA indices, however, resembled DXA indices only for total lean and fat mass but differed substantially for individual regions, especially for fat mass (Fig. 2). For WHI quartiles, the VAT, ASAT, lean and fat mass gradients were overlapping, with the largest differences for all body-composition indices being between the lowest and the highest WHI quartile (Supplementary Fig. S6).

**Fig. 3:**
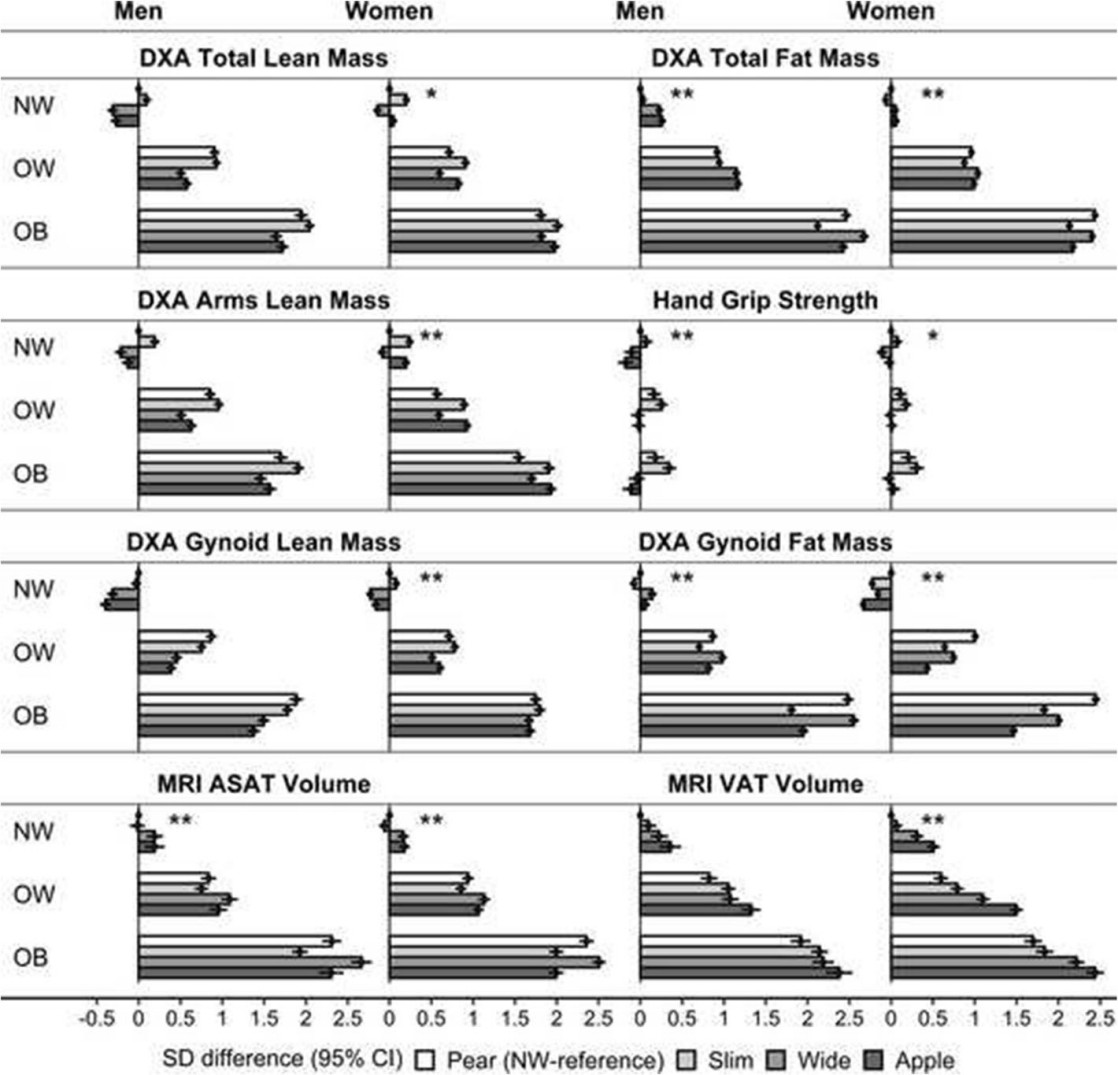
Body composition profiles of categories by body-shape phenotype and BMI. ABSI – a body shape index (cut-offs: ≥80 in men; ≥73 in women); Apple – large-ABSI-small-HI; ASAT – abdominal subcutaneous adipose tissue; BMI – body mass index; CI – confidence interval; DXA – dual-emission X-ray absorptiometry measurements; HI – hip index (cut-offs: ≥49 in men; ≥64 in women); MRI – magnetic resonance imaging measurements; NW – normal weight BMI ≥ 18.5 to < 25.0 kg/m^2^; OB – obese BMI ≥ 30.0 to < 45.0 kg/m^2^; OW – overweight BMI ≥ 25.0 to < 30.0 kg/m^2^; Pear – small-ABSI-large-HI; SD – standard deviation; Slim – small-ABSI-small-HI; VAT – visceral adipose tissue; Wide – large-ABSI-large-HI. SD difference (95% CI) – derived from linear regression models with adjustment for age, self-reported weight change within the year preceding the visit, smoking status, alcohol consumption, physical activity, Townsend deprivation index, region (except for VAT, ASAT and MRI) and, in women, menopausal status and use of hormonal replacement therapy (see definition of covariates in Supplementary Methods and numerical values in Supplementary Table S7). Body-composition measurements were converted to allometric indices with scaling for height (see scaling coefficients in Supplementary Table S2) and then to sex-specific z-scores (value minus mean, divided by the standard deviation). * *p* < 0.05; ** *p* < 0.001 *p*-values from a likelihood ratio test comparing a model with separate ABSI-by-HI and BMI categorical variables with a model with BMI-by-ABSI-by-HI cross-classification. Plots for legs, trunk and android lean and fat mass are shown in Supplementary Fig. S7

Combining BMI categories and body-shape phenotypes showed that lean as well as fat mass in all regions were higher for higher BMI, but higher lean mass was not paralleled by higher hand grip strength (Fig. 3). The patterns of gynoid lean mass and VAT were comparable among BMI categories. In women, gynoid fat mass was consistently lowest for “apple” and highest for “pear” phenotype in all BMI categories. In men, gynoid fat mass resembled the pattern of ASAT in women, with a positive association with hip size in the obese BMI category (highest for “pear” and “wide” phenotypes). ASAT in men, however, showed a different pattern, with the lowest levels for “slim” and highest for “wide” phenotype in the obese BMI category.

### Associations of body-shape phenotypes with colon cancer risk

During a mean follow up of seven years, 1029 colon cancers were ascertained in men and 889 in women.

**Fig. 4:**
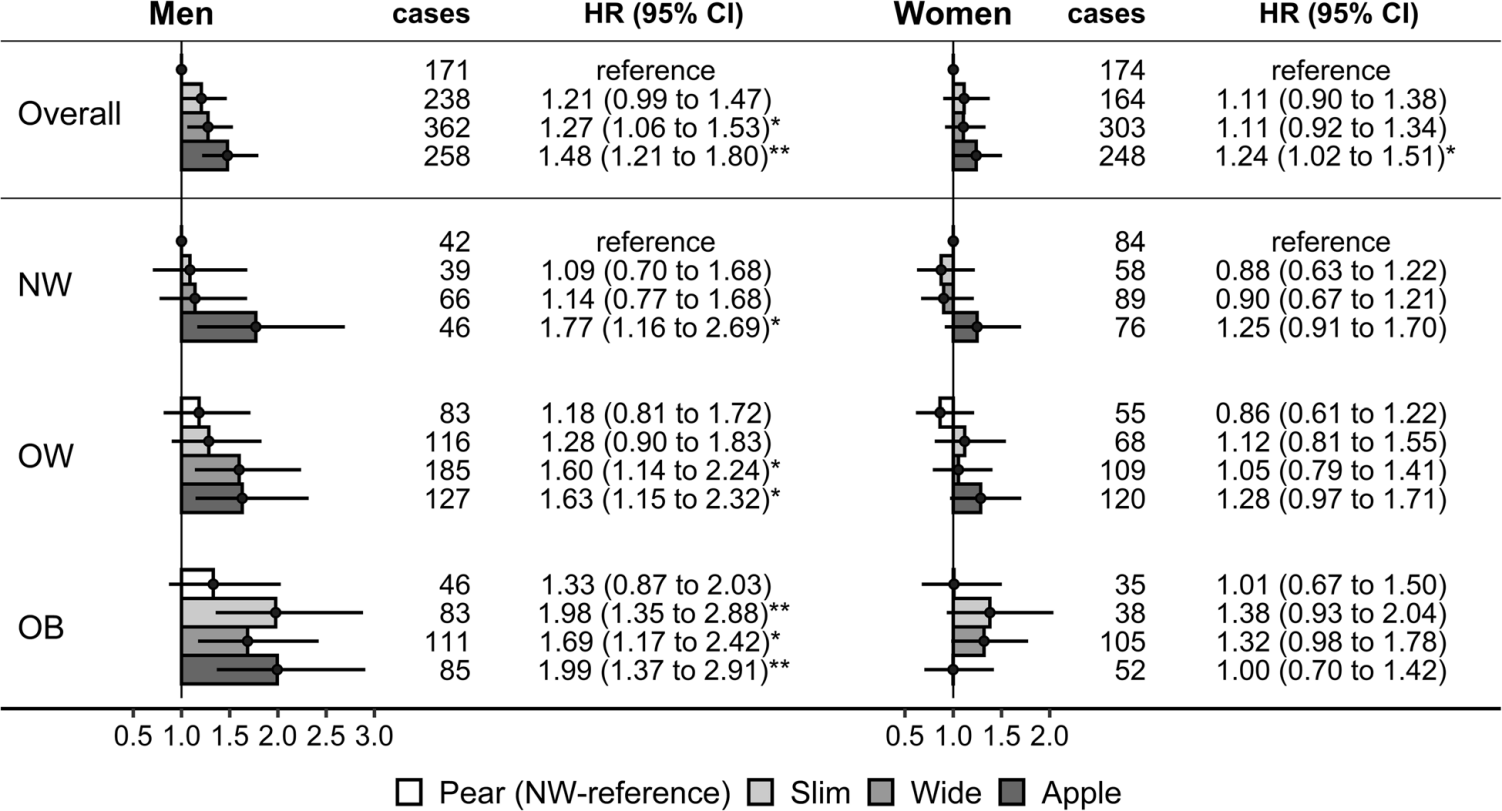
Body-shape phenotypes in relation to colon cancer risk. ABSI – a body shape index (cut-offs: ≥80 in men; ≥73 in women); Apple – large-ABSI-small-HI; BMI – body mass index; CI – confidence interval; HI – hip index (cut-offs: ≥49 in men; ≥64 in women); HR – hazard ratio; NW – normal weight BMI ≥ 18.5 to < 25.0 kg/m^2^; OB – obese BMI ≥ 30.0 to < 45.0 kg/m^2^; OW – overweight BMI ≥ 25.0 to < 30.0 kg/m^2^; Pear – small-ABSI-large-HI; Slim – small-ABSI-small-HI; Wide – large-ABSI-large-HI; HR (95% CI) – derived from cox proportional hazards models, stratified by age and region and adjusted for BMI (for overall), height, self-reported weight change within the year preceding the visit, smoking status, alcohol consumption, physical activity, Townsend deprivation index, diet (consumption of fresh fruit and vegetables, red meat, processed meat, fibre), use of non-steroidal anti-inflammatory drugs, family history of cancer and, in women, menopausal status, use of oral contraceptives and hormonal replacement therapy and age at the last live birth (see definition of covariates in Supplementary Methods). Pairwise comparisons between “apple” and “pear” and between “wide” and “slim” phenotypes for each BMI category are included in Supplementary Table S8

In men, compared to “pear” phenotype, the risk was highest for “apple” (HR = 1.48, 1.21 to 1.80) and intermediate for “slim” (HR = 1.21, 0.99 to 1.47) and “wide” phenotypes (HR = 1.27, 1.06 to 1.53) (Fig. 4). Compared to normal weight BMI, the risk was incrementally higher for overweight (HR = 1.18, 1.00 to 1.40) and obese BMI (HR = 1.43, 1.19 to 1.73) (Supplementary Table S8). In subsets by BMI and body-shape phenotype, however, the risk was incrementally higher according to BMI only for “slim” and “wide” phenotypes. Using the normal weight “pear” phenotype as reference, the risk was higher in men with normal weight BMI only for “apple” phenotype (HR = 1.77, 1.16 to 2.69), additionally higher for “wide” phenotype in men with overweight BMI (HR = 1.60, 1.14 to 2.24), and further additionally higher for “slim” phenotype in men with obese BMI (HR = 1.98, 1.35 to 2.88) (Fig. 4). The risk for “apple” phenotype remained higher compared to “pear” phenotype for all BMI categories but without a marked increment for higher BMI categories (Supplementary Table S8).

In women, associations were weaker compared to men. The risk was higher for “apple” compared to “pear” phenotype overall (HR = 1.24, 1.02 to 1.51), and most clearly in women with overweight BMI (HR = 1.49, 1.08 to 2.05) but not in women with obese BMI, while for “slim” and “wide” phenotypes, there was a suggestion for a positive association only in women with obese BMI (Fig. 4, Supplementary Table S8).

In sensitivity analyses, associations with colon cancer were stronger when omitting the adjustment with covariates but the patterns remained consistent after removing participants with less than two years of follow-up, albeit with a major loss of power (Supplementary Fig. S8).

## Discussion

Our study has shown that, among individuals with the same height and weight, VAT differences were maximised between body-shape phenotypes with discordant waist and hip size (lowest for “pear” and highest for “apple” phenotype), while ASAT differences were maximised between body-shape phenotypes with concordant waist and hip size (lowest for “slim” and highest for “wide” phenotype). In the gynoid region, “apple” and “pear” phenotypes differed mainly by fat mass in women but by lean mass in men, with fat mass contributing in men only at obese BMI. Lean similarly to fat mass was higher for higher BMI but was not paralleled by higher hand grip strength. In men, lean mass was inversely associated with waist size. Compared to normal weight “pear” phenotype, the risk of colon cancer in men was comparably higher for “apple” phenotype in all BMI categories, for “wide” phenotype only for overweight and obese BMI and for “slim” phenotype only for obese BMI. Associations with colon cancer in women were weaker.

Our study is the first to define body-shape phenotypes combining ABSI and HI, to examine their association with body composition using high-quality imaging measurements and to explore heterogeneity by BMI. Several small-scale studies have previously reported, in agreement with our findings, a positive association of ABSI with VAT assessed with BIA [20] or computer tomography [21] and inverse associations of ABSI with hand grip strength [22] and with total fat-free mass assessed with BIA [23, 24] or DXA [21, 25]. Our study, however, has shown that ABSI-by-HI body-shape phenotypes are more informative for body composition than ABSI and HI used individually. Using ABSI alone would not discriminate VAT from ASAT, as ABSI was correlated positively with both, but combining ABSI with HI would enable a discrimination between VAT and ASAT, as VAT and ASAT differences were maximised between non-overlapping pairs of body-shape phenotypes. Further, our study indicates that the higher lean mass corresponding to higher BMI reflects mainly the energy storage capacity of the muscles, as higher lean mass was not matched by correspondingly higher functionality measured with hand grip strength. In agreement, muscles represent the main glycogen storage depot [26] and intervention studies have reported an increase of both lean and fat mass after overfeeding [27]. In our study, we have also compared DXA and BIA measurements of regional fat and lean mass and DXA with MRI measurements of VAT and ASAT with respect to their associations with body-shape phenotypes. DXA has previously shown good agreement with MRI and computer tomography, including for VAT measurement [28], although DXA lean mass is quantified indirectly and could be influenced by body hydration and overestimated in obesity [29, 30]. BIA, however, additionally overestimates fat-free mass in obesity compared to DXA and, most importantly, is disproportionately sensitive to limb and trunk water content [31–33]. Accordingly, our study has demonstrated that BIA does not agree with DXA for regional body composition, which is more relevant to the detrimental consequences of obesity.

Positive associations of the traditional indices of abdominal obesity WC and WHR, as well as BMI, with colon cancer risk, stronger in men than in women, have previously been reported [34], but hip size has not been considered. Large prospective studies examining associations between regional body composition and colon cancer risk are also lacking, although small-scale studies, mainly with case-control design, have reported positive associations with VAT [35]. Recent studies in UK Biobank have also reported for men positive associations with BIA total fat and BIA fat-free mass [36, 37], when examined individually, but only with BIA fat mass when mutually adjusted [36]. The opposition of lean and fat mass and their mutual adjustment, however, do not account for their relatedness as excess energy depots and we have previously demonstrated that combining correlated obesity indices results in biased risk estimates [3, 7]. When we examined BMI, waist and hip circumference were individually, all three were associated positively with colon cancer risk in men [7]. Mutually adjusting all three in the same model converted the positive association with hip circumference to inverse, in agreement with the inverse association with HI, but also abolished the positive association with BMI, which remained independent of adjustment for ABSI and HI [7]. We have also previously demonstrated that using a waist circumference index strongly correlated with BMI does not permit risk stratification by waist size independent of body size, as this classifies as high risk only a very small group of individuals with low BMI and most individuals with high BMI [3]. In the current study, we have similarly shown that it is not possible to use waist and hip circumference to define body-shape phenotypes independent of body size. We would, therefore, advocate replacing waist and hip circumference with ABSI and HI in studies examining associations with body shape. 

The body-composition profiles of body-shape phenotypes would likely be explained, to a great extent, by glucocorticoids and sex-steroids, which are interrelated [15, 38–40]. Cortisol contributes to lean mass reduction by reducing insulin sensitivity and inhibition of glycogen accumulation in skeletal muscles [41, 42]. Correspondingly, high cortisol in patients with Cushing’s syndrome leads to muscle mass reduction and VAT increase, which are ameliorated after adrenalectomy [43, 44]. In men, testosterone has opposite effects to cortisol and glucocorticoid excess suppresses testosterone production [45, 46]. Low testosterone levels in men are associated with higher waist circumference and sarcopenia [47, 48], while testosterone supplementation in hypogonadal men increases muscle mass and reduces VAT [49, 50] and ABSI [21]. VAT reduction, however, requires aromatisation of testosterone to oestradiol [51]. In women, androgens have a different relationship with obesity and VAT. Serum levels of free testosterone are higher in women with abdominal obesity [52] and testosterone administration in female-to-male transsexual conversion increases VAT [53]. In both sexes, morbid obesity contributes to a dysfunction of the hypothalamus-pituitary-gonadal axis, which is alleviated by bariatric surgery, with a resulting increase of testosterone in men and a decrease in women [54].

Oestrogen effects on body composition are also sexually dimorphic and related to glucocorticoids. Thus in women, abdominal obesity and liver steatosis develop after the menopause following a decrease of blood oestrogen levels and oestrogen receptor (ERα) expression in adipose tissue [55–57]. Mouse models have shown that VAT mass gain after ERα reduction in adipocytes is female-specific [58] and the development of liver steatosis after oestrogen reduction is dependent on hypersensitisation of the glucocorticoid receptor [59]. Further, administration of oestrogens combined with the antiandrogen cyproterone acetate in post-menopausal women increases subcutaneous fat specifically in the legs [60], while in male-to-female transsexual conversion the same combination increases subcutaneous fat in both the abdominal and hip areas [53].

Associations of body-shape phenotypes with a given outcome may either be mediated via body composition or may be determined directly by the mechanistic pathways regulating regional body size and composition. If regional fat depots are involved, the outcome would be associated positively with BMI, as fat mass in all regions is higher for higher BMI. In our study, colon cancer risk was positively associated with BMI mainly in men with concordant waist and hip size (“slim” and “wide”). There was also an apparent dependence on critical mass accumulation and a saturation, as compared to normal weight “pear” phenotype, the risk for “wide” phenotype was similarly higher for overweight and obese BMI. This would be compatible with a receptor-mediated mechanism, dependent on higher ligand supply with depot expansion but limited by receptor saturation. Although ASAT was higher for higher BMI, ASAT was consistently lowest for “slim” and highest for “wide” phenotype at any BMI. ASAT would thus reach the critical mass at lower BMI for “wide” phenotype but at higher BMI for “slim” phenotype, thus explaining the higher risk for “slim” phenotype only for obese BMI. A critical mass effect, requiring lowering of BMI below a critical point, may also explain why weight reduction examined on a continuous scale was not associated with a reduction in colon cancer risk [61]. Oestrogens derived from androgen aromatisation in ASAT would be one potential candidate for a ligand, as colon cancer cells appear to favour oestradiol, synthesising it locally via their own aromatase activity [62] and expressing higher levels of ERα, which promotes cell proliferation [63]. Other ligands originating from ASAT such as adipokines, however, may also contribute to the observed associations.

In men with discordant waist and hip size, colon cancer risk appeared related more to the factors determining body shape and less to mass quantity, as the risk was similarly higher for “apple” compared to “pear” phenotype in all BMI categories. Given that “apple” and “pear” phenotypes differed mainly with respect to VAT, we propose that in men with discordant waist and hip size colon cancer is associated primarily with factors determining VAT quantity. Cortisol could be one such factor, as colon cancer cells can synthesise it locally and use it to mediate tumour immune escape via suppression of T-cell activation [64]. Cortisol also stimulates colon cancer cell growth in vitro [65]. The mechanism, however, is likely to be more complicated, involving other interrelated factors such as sex-steroids, insulin resistance, and chronic inflammation and this would need to be clarified in future studies. In any case, our study suggests that a weight reduction without body-shape alteration is unlikely to achieve colon cancer risk reduction for “apple” phenotype in men. Although at present there is no simple answer how to modify body shape, it would be important to ensure that men with “apple” phenotype adhere rigorously to colon cancer screening programs. It would also be important to clarify in future studies how interventions such as modification of diet, hormonal replacement therapy, or physical activity can alter beneficially body shape, although in the case of hormonal therapy, any benefits would have to be balanced against potential risks of hormone-related cancers.

A strength of our study is the sizeable number of participants with body composition measurements and incident colon cancers, which provided statistical power and permitted examining men and women separately. Body composition measurements were obtained with high-quality imaging techniques. Anthropometric measurements were obtained by trained personnel, avoiding bias from self-reported values. Models were adjusted for major lifestyle factors, minimising confounding. However, due to lack of adequate data, we could not examine directly associations between body composition and cancer risk, or ethnic variations in body composition, which are known to be large [13, 66], or heterogeneity by menopausal status (there were no younger pre-menopausal women in the imaging datasets), or heterogeneity by obesity grade, or extreme obesity, or longitudinal changes in time. Furthermore, UK Biobank participants are not representative of the overall population [67]. This discrepancy was even more prominent for participants in the imaging datasets, which were less obese and with healthier lifestyles than the cohort at enrolment.

## Conclusion

While BMI provides information for the quantity of fat and lean mass overall, ABSI-by-HI body-shape phenotypes provide information for body composition, reflecting differences between VAT and ASAT and between gynoid lean and fat mass among individuals with the same weight and height. Our results are compatible with a leading contribution of gynoid fat to differences in hip size in women but suggest a different role and regulation of gynoid fat in men, possibly similar to ASAT in women. Body-shape phenotypes show differences in body composition at all BMI levels and convey information for a separate aspect of obesity, independent from energy balance. Colon cancer risk in men is lowest in “pear” phenotype and appears related to ASAT quantity for “slim” and “wide” phenotypes but to factors determining VAT accumulation for “apple” phenotype. Examining associations with body-shape phenotypes in addition to associations with BMI would enhance studies of obesity related outcomes. We would recommend using the ABSI-by-HI body-shape phenotypes and the BMI-by-ABSI-by-HI cross-classification in studies examining obesity and cancer risk to obtain risk stratification by body shape independent of body size, as well as to provide insights for associations with body composition and hypotheses for future investigation.

## Supplementary Information

**Additional file 1.**

## Data Availability

The data supporting the findings of the study are available *to* bona fide researchers upon approval of an application to the UK Biobank (https://www.ukbiobank.ac.uk/researchers/) and a material transfer agreement.

## Abbreviations

ABSI: A body shape index
BIA: Bioelectric impedance analysis
BMI: Body mass index
CI: Confidence interval
DXA: Dual-emission X-ray absorptiometry
HC: Hip circumference
HI: Hip index
HR: Hazard ratio
MRI: Magnetic resonance imaging
NHANES: National Health and Nutrition Examination Survey
SD: Standard deviation
UK: United Kingdom
WC: Waist circumference
WHI: Waist-to-hip index
WHR: Waist-to-hip ratio
