# Supplementary material for: Association of body-shape phenotypes with imaging measures of body composition in the UK Biobank cohort: relevance to colon cancer risk
Source: BMC Cancer. 2021 Oct 15;21:1106. doi: 10.1186/s12885-021-08820-6 (PMC8518225; doi:10.1186/s12885-021-08820-6)
Supplement: Supplementary file 1 — Additional file 1. [file 12885_2021_8820_MOESM1_ESM.pdf]

Association of body-shape phenotypes with imaging measures of body composition in the UK Biobank cohort: relevance to colon cancer risk

Sofia Christakoudi, Konstantinos K. Tsilidis, Evangelos Evangelou, Elio Riboli

Supplementary Methods

Ascertainment of cancer cases 3

Definition of body composition variables 4

Definition of covariates ……. 6

Supplementary Tables

Supplementary Table S1 Overlap between BMI-by-ABS-by-HI phenotypes defined with HI calculated with coefficients from UK Biobank or NHANES in men ... 13

Supplementary Table S2 Scaling coefficients for allometric body composition indices 14

Supplementary Table S3 Characteristics of study participants according to ABSI-by-HI body-shape phenotype 16

Supplementary Table S4 Anthropometric characteristics of study participants according to waist-by-hip circumference phenotype 20

Supplementary Table S5 Overlap between ABSI-by-HI phenotypes and WHI quartiles 22

Supplementary Table S6 Body-composition of body-shape phenotypes 23

Supplementary Table S7 Body-composition of body-shape phenotypes by BMI category 25

Supplementary Table S8 Body-shape phenotypes in relation to colon cancer risk 28

Supplementary Figures

Supplementary Figure S1 Flow diagram of UK Biobank participants included in the study 29

Supplementary Figure S2 Correlation between anthropometry and body composition among individuals with the same height 31

Supplementary Figure S3 Correlation between anthropometry and body composition among individuals with the same height and weight 32

Supplementary Figure S4 Body composition profiles of body-shape phenotypes (DXA VAT subset) 33

Supplementary Figure S5 Body composition profiles of body-shape phenotypes (minimally adjusted models) 35

Supplementary Figure S6 Body composition profiles of waist-to-hip index quartiles 36

Supplementary Figure S7 Body composition profiles of categories by body-shape phenotype and BMI category (extension) 38

Supplementary Figure S8 Body-shape phenotypes in relation to colon cancer risk (sensitivity analyses) 39

References 40

Supplementary Methods

Ascertainment of cancer cases

Prevalent cancer cases were defined as cancer diagnosed up to the date of the visit (i.e. the date of attending an assessment centre at enrolment or the date of the imaging visit). Information for prevalent cancers was obtained from the cancer registry and from self-reported cancer. Information for cancer diagnosis in the cancer registry was included in Fields [40006-0.0/16] "*Type of cancer: ICD10*" (codes starting with "C") or Fields [40013-0.0/14] "*Type of cancer: ICD9*" (codes 140 to 209). Excluded from cancer cases were tumours with behavioural code 0 "*Benign*", 1 "*Uncertain whether benign or malignant*" or 2 "*Carcinoma in situ*" (obtained from Fields [40012-0.0/16] "*Behaviour of cancer tumour*"), as well as non-melanoma skin cancers (defined with code 173 for ICD9 or C44 for ICD10), except skin cancers with squamous cell morphology (codes 8070, 8071, 8072 or 8083 from Fields [40011-0.0/16] "*Histology of cancer tumour*"). Further details on the data management of cancer registry fields are given in reference [7]. Information on self-reported prevalent cancer was obtained from Fields [20001-0.0/5] (at enrolment) or Fields [20001-2.0/5] (at the imaging visit) "*Cancer code, self-reported*" (excluding codes: 1003 "*skin cancer*", 1060 "*non-melanoma skin cancer*", 1061 "*basal cell carcinoma*", 1073 "*rodent ulcer*", 1072 "*cin/pre-cancer cells cervix*"). Prevalent cancer was considered present, if there was a cancer registry entry up to the date of visit, OR a self-reported prevalent cancer at the date of the visit.

Incident cancer cases were considered entries in the cancer registry Fields [40006-0.0/31] "*Type of cancer: ICD10*" with code C18, behavioural code from Fields [40012-0.0/31] "*Behaviour of cancer tumour*" defined as 3 "*malignant, primary site*" or 5 *"Malignant, microinvasive"* and date of diagnosis after the date of attending an assessment centre at enrolment (for associations with colon cancer risk) or after the date of the imaging visit (for associations with body composition, used only as an exclusion criterion). Non-melanoma skin cancers (code C44) were excluded, except those with squamous cell morphology (codes 8070, 8071, 8072 or 8083 from Fields [40011-0.0/31] "*Histology of cancer tumour*"). Date of diagnosis corresponded to the first incident cancer. Participants with first incident cancer with topological code other than C18 or with behavioural code 6 "*Malignant, metastatic*", 9 "*Malignant, uncertain whether primary or metastatic site*" or missing were censored at the date of cancer diagnosis.

Date of death was obtained from Field [40000-0.0].

Definition of body composition variables

Dual-emission X-ray absorptiometry (DXA)

DXA Visceral Adipose Tissue (VAT) Mass was provided in Field [23288-2.0] and was based on an algorithm subtracting ASAT from the android region, as described in reference [28]. We calculated DXA Abdominal Subcutaneous Adipose Tissue (ASAT) Mass as:

DXA ASAT Mass = DXA Android Fat Mass – DXA VAT Mass

DXA regions were defined as follows: "**arms**" included the arms and shoulders areas; "**trunk**" included the neck, chest, abdominal and pelvic areas; "**legs**" included all remaining areas below the trunk; "**android**" overlapped the trunk region between the ribs and the pelvis; "**gynoid**" overlapped the leg and trunk regions, including the hips and upper thighs, as described in reference [13].

Only measurements for total **Regional Mass** and **Regional Tissue Fat Percentage** were provided for the complete DXA dataset. Individual measurements for Regional Fat Mass and Regional Lean Mass were provided only for the DXA VAT subset (see Supplementary Figure S1 for definition of datasets). The units of measurement were grams. The corresponding Field numbers are listed below:

Region	Regional Fat Mass (g)	Regional Lean Mass (g)	Regional Mass (g)	Tissue Fat Percentage
Arms	23257-2.0	23258-2.0	23260-2.0	23259-2.0
Legs	23274-2.0	23275-2.0	23277-2.0	23276-2.0
Gynoid	23262-2.0	23263-2.0	23265-2.0	23264-2.0
Trunk	23284-2.0	23285-2.0	23287-2.0	23286-2.0
Android	23245-2.0	23246-2.0	23248-2.0	23247-2.0
Total	23278-2.0	23279-2.0	23283-2.0	23281-2.0
Available	DXA VAT subset	DXA VAT subset	DXA dataset	DXA dataset

We calculated **Regional Fat Mass** and **Regional Lean Mass** values for the complete DXA dataset based on the DXA VAT subset as follows:

1. Using the DXA VAT subset, we calculated **Regional Tissue Mass** as:

   Regional Tissue Mass = Regional Fat Mass + Regional Lean Mass

2. We then calculated for each region **Regional Tissue Percentage** as the proportion from total Regional Mass (equivalent to the sum of Fat, Lean and Bone Mass) as:

   Regional Tissue Percentage = Regional Tissue Mass / Regional Mass

3. To calculate **Regional Tissue Mass** for the complete DXA dataset, we used Regional Mass and the median Regional Tissue Percentage per sex and strata of age at the imaging visit (dichotomised at ≥65 years), body mass index (BMI, dichotomised at ≥25 kg/m^2) and ABSI-by-HI body-shape phenotype (a body shape index by hip index, see the definition of body-shape phenotypes in the main Methods section Anthropometric indices):

   *Regional Tissue Mass = Regional Mass * Regional Tissue Percentage*

The stratum-specific Regional Tissue Percentages based on the DXA VAT subset are summarised below:

Age	BMI	Shape	N	Total	Arms	Legs	Gynoid	Trunk	Android
**WOMEN**									
< 65 years	< 25 kg/m²	Pear	153	0.9638 (0.005)	0.9552 (0.007)	0.9622 (0.006)	0.9784 (0.004)	0.9787 (0.004)	0.9899 (0.003)
		Slim	228	0.9622 (0.007)	0.9543 (0.009)	0.9604 (0.007)	0.9768 (0.005)	0.9778 (0.006)	0.9896 (0.003)
		Wide	124	0.9647 (0.007)	0.9538 (0.010)	0.9610 (0.008)	0.9777 (0.004)	0.9799 (0.006)	0.9901 (0.004)
		Apple	121	0.9637 (0.005)	0.9555 (0.007)	0.9594 (0.007)	0.9767 (0.006)	0.9795 (0.004)	0.9909 (0.003)
	≥ 25 kg/m²	Pear	156	0.9695 (0.005)	0.9633 (0.008)	0.9685 (0.006)	0.9810 (0.004)	0.9812 (0.005)	0.9918 (0.003)
		Slim	213	0.9685 (0.005)	0.9627 (0.008)	0.9672 (0.006)	0.9799 (0.004)	0.9812 (0.004)	0.9920 (0.002)
		Wide	163	0.9703 (0.006)	0.9629 (0.009)	0.9681 (0.006)	0.9806 (0.004)	0.9825 (0.004)	0.9928 (0.003)
		Apple	202	0.9692 (0.005)	0.9629 (0.007)	0.9658 (0.005)	0.9792 (0.003)	0.9817 (0.004)	0.9929 (0.002)
≥ 65 years	< 25 kg/m²	Pear	97	0.9662 (0.005)	0.9584 (0.007)	0.9641 (0.006)	0.9792 (0.004)	0.9809 (0.005)	0.9907 (0.003)
		Slim	109	0.9659 (0.006)	0.9584 (0.009)	0.9628 (0.007)	0.9781 (0.004)	0.9808 (0.004)	0.9903 (0.003)
		Wide	93	0.9655 (0.006)	0.9584 (0.009)	0.9624 (0.008)	0.9775 (0.004)	0.9811 (0.005)	0.9909 (0.004)
		Apple	70	0.9649 (0.006)	0.9576 (0.011)	0.9601 (0.006)	0.9775 (0.004)	0.9807 (0.004)	0.9912 (0.003)
	≥ 25 kg/m²	Pear	87	0.9718 (0.006)	0.9636 (0.010)	0.9706 (0.005)	0.9821 (0.003)	0.9830 (0.003)	0.9927 (0.003)
		Slim	111	0.9697 (0.006)	0.9638 (0.008)	0.9673 (0.007)	0.9806 (0.003)	0.9818 (0.004)	0.9920 (0.003)
		Wide	144	0.9706 (0.005)	0.9643 (0.008)	0.9679 (0.008)	0.9812 (0.004)	0.9829 (0.004)	0.9927 (0.002)
		Apple	130	0.9690 (0.005)	0.9641 (0.007)	0.9654 (0.006)	0.9791 (0.004)	0.9822 (0.004)	0.9922 (0.003)
**MEN**									
< 65 years	< 25 kg/m²	Pear	87	0.9598 (0.006)	0.9470 (0.008)	0.9519 (0.007)	0.9716 (0.006)	0.9772 (0.005)	0.9899 (0.004)
		Slim	150	0.9593 (0.004)	0.9487 (0.007)	0.9508 (0.007)	0.9715 (0.005)	0.9768 (0.004)	0.9900 (0.003)
		Wide	51	0.9599 (0.004)	0.9475 (0.008)	0.9518 (0.006)	0.9723 (0.005)	0.9769 (0.004)	0.9906 (0.004)
		Apple	30	0.9603 (0.004)	0.9473 (0.008)	0.9526 (0.007)	0.9723 (0.004)	0.9778 (0.005)	0.9904 (0.003)
	≥ 25 kg/m²	Pear	211	0.9639 (0.006)	0.9545 (0.007)	0.9567 (0.007)	0.9748 (0.005)	0.9786 (0.004)	0.9921 (0.003)
		Slim	444	0.9640 (0.006)	0.9548 (0.007)	0.9565 (0.007)	0.9744 (0.005)	0.9788 (0.005)	0.9920 (0.003)
		Wide	99	0.9649 (0.006)	0.9546 (0.007)	0.9572 (0.007)	0.9754 (0.005)	0.9797 (0.004)	0.9927 (0.003)
		Apple	71	0.9655 (0.005)	0.9553 (0.008)	0.9578 (0.007)	0.9747 (0.004)	0.9796 (0.005)	0.9926 (0.003)
≥ 65 years	< 25 kg/m²	Pear	77	0.9586 (0.006)	0.9473 (0.006)	0.9501 (0.006)	0.9705 (0.005)	0.9770 (0.004)	0.9897 (0.004)
		Slim	80	0.9581 (0.005)	0.9468 (0.007)	0.9493 (0.006)	0.9706 (0.005)	0.9769 (0.006)	0.9900 (0.004)
		Wide	94	0.9603 (0.006)	0.9477 (0.008)	0.9505 (0.007)	0.9724 (0.006)	0.9781 (0.005)	0.9909 (0.003)
		Apple	32	0.9594 (0.005)	0.9475 (0.008)	0.9495 (0.007)	0.9714 (0.005)	0.9775 (0.005)	0.9909 (0.003)
	≥ 25 kg/m²	Pear	114	0.9638 (0.006)	0.9541 (0.008)	0.9567 (0.008)	0.9746 (0.007)	0.9790 (0.005)	0.9920 (0.003)
		Slim	254	0.9635 (0.006)	0.9526 (0.008)	0.9552 (0.008)	0.9738 (0.005)	0.9788 (0.005)	0.9918 (0.003)
		Wide	147	0.9641 (0.006)	0.9532 (0.008)	0.9559 (0.007)	0.9742 (0.005)	0.9792 (0.005)	0.9920 (0.003)
		Apple	89	0.9633 (0.006)	0.9525 (0.008)	0.9540 (0.009)	0.9736 (0.006)	0.9788 (0.005)	0.9922 (0.003)

Values in the cells represent median (interquartile range); **N** – number of participants; **Apple** – large-ABSI-small-HI; **Pear** – small-ABSI-large-HI; **Slim** – small-ABSI-small-HI; **Wide** – large-ABSI-large-HI.

4. Finally, we calculated **Regional Fat Mass** and **Regional Lean Mass** for the complete DXA dataset using Regional Tissue Mass and Regional Tissue Fat Percentage (note that the latter was provided as a proportion):

*Regional Fat Mass = Regional Tissue Mass * Regional Tissue Fat Percentage*

Regional Lean Mass = Regional Tissue Mass – Regional Fat Mass

For the DXA VAT subset, Pearson correlations coefficients between the original and the predicted Regional Fat and Lean Mass values were >0.99.

Magnetic Resonance Imaging (MRI)

MRI VAT was defined as the adipose tissue within the abdominal cavity, excluding adipose tissue outside the abdominal skeletal muscles and adipose tissue and lipids within and posterior of the spine and posterior of the back muscles, as described in reference [14]. MRI ASAT was defined as subcutaneous adipose tissue in the abdomen from the top of the femoral head to the top of the thoracic vertebrae T9. MRI VAT Volume was obtained from Field [22407-2.0] and MRI ASAT Volume was obtained from Field [22408-2.0]. MRI measurements were provided in litres. Values were considered missing if there was any flag in Fields [22414-2.0/1] *Image quality indicator* OR Field [22411-2.0] *VAT/ASAT error indicator*. Total Abdominal Adipose Tissue Volume, which is analogous to DXA Android Fat Mass, was calculated as:

MRI Abdominal Adipose Tissue Volume = MRI VAT Volume + MRI ASAT Volume

Bioelectrical Impedance Analysis (BIA)

BIA measurements were obtained from Fields:

	Total (kg)	Arms (kg)	Legs (kg)	Trunk (kg)
Fat Mass	23100-2.0	23120-2.0 (right), 23124-2.0 (left)	23112-2.0 (right), 23116-2.0 (left)	23128-2.0
Fat-free Mass	23101-2.0	23121-2.0 (right), 23125-2.0 (left)	23113-2.0 (right), 23117-2.0 (left)	23129-2.0

Values for Arms and Legs were calculated as the sum of the right and left variable. Fat-free Mass includes lean and bone mass. Fat Mass and Fat-free Mass add up to the total mass of the region. Note that this differs from DXA measurements, where lean and bone mass are measured separately and Regional Mass comprises the sum of Regional Fat, Lean and Bone Mass.

Hand Grip Strength

We considered hand grip strength in the context of body composition as an indicator of muscle mass functionality. This was calculated as the maximum of Field [46-2.0] *(left)* and Field [47-2.0] *(right)* OR, when values in one of the two fields were missing, as the available value. Measurements were considered missing when values were missing in both Fields.

Definition of covariates

Extensions Field-0.X correspond to the initial assessment visit at enrolment and extensions Field-2.X correspond to the imaging visit. Fields for variables evaluated both at enrolment and at the imaging visit are marked as Field-0(2).X.

Date of birth was constructed from Field [34-0.0] "*Year of birth*" and Field [52-0.0] "*Month of birth*", using 15 as the day of birth for all participants.

Age at enrolment or at the imaging visit was calculated in years as the difference between Field [53-0(2).0] "*Date of attending assessment centre*" and the date of birth (as defined above), divided by 365.25 and rounded to an integer. Six five-year categories for age at enrolment were

used for stratification of Cox proportional hazards models when examining associations with colon cancer risk (40 to <45 years, 45 to <50 years, 50 to <55, 55 to <60 years, 60 to <65 years and 65 to 70 years). Age at the imaging visit was used as a continuous adjustment variable when examining associations with body composition.

Region of the assessment centre at enrolment or at the imaging visit was based on Field [54-0(2).0] "*UK Biobank assessment centre*". Assessment centres at **enrolment** were grouped in 10 geographic regions as follows: London, including Barts (centre ID number 11012), Hounslow (11018) and Croydon (11020); North-West, including Bury (11008), Liverpool (11016), Manchester (11001) and Stockport (pilot, 10003); North-East, including Middlesbrough (11017) and Newcastle (11009); Yorkshire and Humber, including Leeds (11010) and Sheffield (11014); West Midlands, including Birmingham (11021) and Stoke (11006); East Midlands, including Nottingham (11013); South-East, including Oxford (11002) and Reading (11007); South-West, including Bristol (11011); Wales, including Cardiff (11003), Swansea (11022) and Wrexham (11023); Scotland, including Edinburgh (11005) and Glasgow (11004). Assessment centres at the **imaging visit** were grouped in 3 geographic regions as follows: North-West, including Cheadle (11025); North-East, including Newcastle (11027) and South, including Reading (11026) and Bristol (11028).

Weight change during last year preceding the initial assessment at enrolment or the imaging visit was self-reported in Field [2306-0(2).0] "*Weight change compared with 1 year ago*"; Question: "*Compared with one year ago, has your weight changed?*" with three valid answers: 0 *"No - weigh about the same"* (category Stable weight), 2 *"Yes - gained weight"* (category Weight gain), 3 *"Yes - lost weight"* (category Weight loss). Participants with missing information were assigned to the median sex-specific category (Stable weight).

Smoking status at enrolment or at the imaging visit was defined as follows: Current smoker – was based on Field [1239-0(2).0] "*Current tobacco smoking*"; Question: *"Do you smoke tobacco now?"*; Answer 1: "*Yes, on most or all days*" or 2: "*Only occasionally*"; Former regular smoker – was based on Field [1249-0(2).0] "*Past tobacco smoking*"; Question: "*In the past, how often have you smoked tobacco?*"; Answer 1: "*Smoked on most or all days*", when the answer to Filed [1239-0.0] was not 1 or 2; Former occasional smoker – was based on Field [1249-0(2).0] Answer 2: "*Smoked occasionally*" or 3: "*Just tried once or twice*" when the answer to Filed [1239-0(2).0] was not 1 or 2; Never smoked – was based on Field [1249-0(2).0] Answer 4: "*I have never smoked*" when the answer to [Filed 1239-0(2).0] was not 1 or 2. Participants with missing information were assigned to the median sex-specific category (Former occasional smoker).

Alcohol consumption at enrolment or at the imaging visit was based on Field [1558-0(2).0] "*Alcohol intake frequency*"; Question: "*About how often do you drink alcohol?*" as follows: Up to 3 times a month – Answer 4: "*One to three times a month*"; or 5: "*Special occasions only*"; or 6: "*Never*"; Up to four times a week – Answer 2: "*Three or four times a week*"; or 3: "*Once or twice a week*"; Daily or almost daily – Answer 1: "*Daily or almost daily*". Participants with missing information were assigned to the median sex-specific category (Up to four times a week).

Physical activity at enrolment or at the imaging visit was defined as follows: Very active – was based on Field [816-0(2).0] "*Job involves heavy manual or physical work*"; Question: "*Does your work involve heavy manual or physical work?*"; Answer 3: "*Usually*" or 4: "*Always*"; OR Field [904-0(2).0] "*Number of days/week of vigorous physical activity 10+ minutes*"; Question: "*In a typical WEEK, how many days did you do 10 minutes or more of vigorous physical activity? (These are activities that make you sweat or breathe hard such as fast cycling, aerobics, heavy lifting)*"; Answer (numerical) 3-7; Moderately active – was based Field [904-0(2).0] Answer 1-2 OR Field [884-0(2).0] "*Number of days/week of moderate physical activity 10+ minutes*"; Question: "*In a typical WEEK, on how many days did you do 10 minutes or more of moderate physical activities like carrying light loads, cycling at normal pace? (Do not include walking)*"; Answer (numerical) 3-7; OR Field [864-0(2).0] "*Number of days/week walked 10+ minutes*"; Question: "*In a typical WEEK, on how many days did you walk for at least 10 minutes at a time? (Include walking that you do at work, travelling to and from work, and for sport or leisure)*"; Answer (numerical) 7, when participants were not already included in category very active; Less active - was based on Field [904-0(2).0] Answer (numerical) 0 OR Field [884-0(2).0] Answer (numerical) 0-2 OR Field [864-0(2).0] Answer (numerical) 0-6 or -2: "*Unable to walk*", when participants were not already included in category moderately or very active. Participants with missing information were assigned to the median sex-specific category (Moderately active).

Townsend deprivation index (available only at enrolment) was used as an indicator of socioeconomic status and was based on Field [189-0.0] "*Townsend deprivation index at recruitment*", which was calculated by UK Biobank as a score corresponding to the output area in which the participant's postcode was located immediately prior to joining UK Biobank, based on the preceding national census output areas. A greater score implies a greater degree of material deprivation. This variable was categorised in sex-specific tertiles. The tertile boundaries were: -3.229 and -0.797 for men and -3.220 and -0.880 for women in the cancer risk dataset; -3.526 and -1.547 for men and -3.451 and -1.410 for women in the DXA dataset; -3.512 and -1.489 for men and -3.451 and -1.470 for women in the DXA VAT subset; -3.494 and -1.506 for men and -3.464 and -1.434 for women in the MRI dataset (see Supplementary Figure S1 for definition of datasets). Participants with missing information were assigned to the median sex-specific category (Middle tertile).

Family history of cancer at enrolment was based on three variables: Fields [20107-0.0/9] "*Illness of father*", Question: "*Has/did your father ever suffer from? (You can select more than one answer)"*, Fields [20110-0.0/10] "*Illness of mother*", Question: *"Has/did your mother ever suffer from? (You can select more than one answer)"* and Field [20111-0.0/11] "*Illness of siblings*", Question: "*Have any of your brothers or sisters suffered from any of the following diseases? (You can select more than one answer)".* Category Yes (bowel) was based on Answer: 4 "*Bowel cancer*" to any of the three sets of fields; Category Yes (lung, breast, prostate) was based on Answer: 3

"*Lung cancer*", 5 "*Breast cancer*" or 13 "*Prostate cancer*" to any of the three sets of fields and category No included the remaining participants.

Fresh fruit and vegetable intake at enrolment was based on the sum of two fields: Field [1309-0.0] "*Fresh fruit intake*" (continuous), Question: "*About how many pieces of FRESH fruit would you eat per DAY? (Count one apple, one banana, 10 grapes etc as one piece; put '0' if you do not eat any)*" and Field [1299-0.0] "*Salad / raw vegetable intake*" (continuous), Question: "*On average how many heaped tablespoons of SALAD or RAW vegetables would you eat per DAY? (Include lettuce, tomato in sandwiches; put '0' if you do not eat any)*". Answers: -10 *"Less than one"* were re-coded to 0.5. Answers: -1 "*Do not know*" and -3 "*Prefer not to answer*" were consider missing. The total was dichotomised as Less than five portions a day or Five or more portions a day and were used as an indication of a healthy lifestyle. Participants with missing information were assigned to the median sex-specific category (Less than five portions a day).

Processed meat intake at enrolment was based on Field [1349-0.0] "*Processed meat intake*", Question: *"How often do you eat processed meats (such as bacon, ham, sausages, meat pies, kebabs, burgers, chicken nuggets)"*. Category Less than twice a week included answers: 0 "Never", 1 "Less than once a week" and 2 "Once a week". Category Twice or more a week included answers: 3 "*2-4 times a week*", 4 "*5-6 times a week*" 5 "*Once or more daily*". Answers: -1 "*Do not know*" and -3 "*Prefer not to answer*" were consider missing. Participants with missing information were assigned to the median sex-specific category (Less than twice a week).

Red meat intake at enrolment was based on the sum of three fields: Field [1369-0.0] "*Beef intake*", Question: "*How often do you eat beef? (Do not count processed meats)*", Field [1379-0.0] "*Lamb/mutton intake*", Question: "*How often do you eat lamb/mutton? (Do not count processed meats)*" and Field [1389-0.0] "*Pork intake*", Question: *"How often do you eat pork? (Do not count processed meats such as bacon or ham)"*. The categorical answers were converted to a continuous scale as follows: Answer 0 "Never" remained 0; Answer 1 "Less than once a week" was coded as 0.5; Answer 2 "Once a week" was coded as 1; Answer 3 "2-4 times a week" was coded as 3; Answer 4 "5-6 times a week" was coded as 5.5; Answer 5 "Once or more daily" was coded as 7. Answers: -1 "*Do not know*" and -3 "*Prefer not to answer*" were consider missing. Categories Less than twice a week and Twice or more a week were derived with respect to the total of the three variables. Participants with missing information were assigned to the median sex-specific category (Less than twice a week for women and Twice or more a week for men).

Fibre intake at enrolment was based on the sum of fibre allocated to the consumption of fruit, vegetables, bread and cereal, using estimated quantities of fibre per food type and quantity according to reference [17] as follows: Field [1309-0.0] "*Fresh fruit intake*" (continuous) Question: *"About how many pieces of FRESH fruit would you eat per DAY? (Count one apple, one banana, 10 grapes etc as one piece; put '0' if you do not eat any)"* allocating 2g of fibre per piece; Field [1319-0.0] "*Dried fruit intake*" (continuous) Question: *"About how many pieces of DRIED fruit would you eat per DAY? (Count one prune, one dried apricot, 10 raisins as one piece; put '0' if you do not*

eat any)" allocating 0.5g of fibre per piece; Field [1299-0.0] "*Salad / raw vegetable intake*" (continuous) Question: *"On average how many heaped tablespoons of SALAD or RAW vegetables would you eat per DAY? (Include lettuce, tomato in sandwiches; put '0' if you do not eat any)"* allocating 1g of fibre per tablespoon; Field [1289-0.0] "*Cooked vegetable intake*" (continuous) Question: *"On average how many heaped tablespoons of COOKED vegetables would you eat per DAY? (Do not include potatoes; put '0' if you do not eat any)"* allocating 1g of fibre per tablespoon; Field [1438-0.0] "*Bread intake*" (continuous) Question: *"How many slices of bread do you eat each WEEK?"* (divided by 7 to obtain the slices of bread per day) and Field [1448-0.0] "*Bread type*" (categorical) Question: *"What type of bread do you mainly eat?"* allocating 0.68g per slice of "White" bread (code 1), 1.26g per slice of "Brown" bread (code 2), 1.80g per slice of "Wholegrain or wholemeal" bread (code 3), 1.25g per slice of "Other type of bread" (code 4) or missing bread type; Field [1458-0.0] "*Cereal intake*" (continuous) Question: *"How many bowls of cereal do you eat a WEEK?"* (divided by 7 to obtain the cereal bowls per day) and Field [1468-0.0] "*Cereal type*" (categorical) Question: *"What type of cereal do you mainly eat?"* allocating 7.16g per bowl of "*Bran cereal (e.g. All Bran, Branflakes)*" (code 1), 2.92g per bowl of "*Biscuit cereal (e.g. Weetabix)"* (code 2), 1.92g per bowl of "*Oat cereal (e.g. Ready Brek, porridge)*" (code 3), 4.18g per bowl of "Muesli" (code 4), 0.54g per bowl of "*Other (e.g. Cornflakes, Frosties)*" (code 5), or 3.34g per bowl if missing cereal type. For quantitative variables, answer -10 "*Less than one*" was re-coded to 0.5 and answers -1 "*Do not know*" and -3 "*Prefer not to answer*" were considered missing. This variable was categorised in sex-specific tertiles. The tertile boundaries were: 11.92 g and 17.13 g for men and 12.52 g and 17.11 g for women. Information for fibre intake was missing when information for all variables used to calculate the quantity was missing. Participants with missing information were assigned to the median category (Middle tertile).

Nonsteroidal Anti-inflammatory Drugs (Aspirin & Ibuprofen) use at enrolment was based on Fields [6154-0.1/5] "*Medication for pain relief, constipation, heartburn*"; Question: "*Do you regularly take any of the following? (You can select more than one answer)*"; Answer 1: "*Aspirin*" OR Answer 2: "*Ibuprofen*" (for category Yes). The guidance included the message: "*Some over the counter medicines are known by other names. Please enter the corresponding name if you take any of the following REGULARLY (that is, most days of the week for the last 4 weeks): Aspirin (Alka Rapid Crystals, Alka-Seltzer XS, Anadin Extra, Anadin, Original, Askit powders, Aspro Clear, Codis 500, Disprin, Disprin Extra) OR Ibuprofen (Anadin Ultra, Anadin Ibuprofen, Cuprofen Plus, Nurofen, Solpaflex, Ibuleve)".* Category No was defined as any of the following Answers: 3 "*Paracetamol*", 4 "*Ranitidine*", 5 "*Omeprazole*", 6 "*Laxatives*" OR -7 "*None of the above*". Values were considered missing if there was no answer to this question or Answer -1 "*Do not know*" or Answer -3 "*Prefer not to answer*" were given. Missing values were replaced with the sex-specific median (No).

Hormone replacement therapy (HRT) use at enrolment or at the imaging visit was determined for women by Field [2814-0(2).0] "*Ever used hormone-replacement therapy (HRT)*"; Question: "*Have you ever used hormone replacement therapy (HRT)?*"; Answer 0: "*No*" (for Never user) or Answer 1: "Yes" and Field [3546-0(2).0] "*Age last used hormone-replacement therapy (HRT)*" Question: *"How old were you when you last used HRT?"* Answer -11: "*Still taking HRT"* (for Current user) or else Answer 1: "Yes" to Field [2814-0(2).0] (for Former user). Further information was derived from Fields [6153-0(2).0/3] "*Medication for cholesterol, blood pressure, diabetes, or take exogenous hormones*", Question: "*Do you regularly take any of the following medications? (You can select more than one answer)"*. Women providing Answer 4 "*Hormone replacement therapy*" were considered Current user. Women with missing information for Field [2814-0(2).0] were assigned to the median category (Never user).

Menopausal status (MP) at enrolment was determined as follows: Post-menopausal (Post-MP) – were classified women with age at enrolment ≥58 years OR with bilateral oophorectomy from Field: [2834-0.0] "*Bilateral oophorectomy (both ovaries removed)*"; Question: "*Have you had BOTH ovaries removed?*"; Answer 1: "*Yes*" OR Field [20004-0.0/31] "*Operation code (self-reported operation)*" code: 1355 "*bilateral oophorectomy*"; OR with self-reported post-menopausal status from Field [2724-0.0] "*Had menopause*"; Question: "*Have you had your menopause (periods stopped)?*"; Answer 1: "Yes" OR with age at enrolment ≥ 55 years when menopausal status was unknown, i.e. they had not answered 0: "No" to Field [2724-0.0]; Pre-menopausal (Pre-MP) – were classified women who had not been defined as post-menopausal above and had reported pre-menopausal status with Answer 0: "No" to Field [2724-0.0] OR had age at enrolment < 55 years when menopausal status was unknown, i.e. not defined as post- or pre-menopausal according to the above criteria.

Menopausal status (MP) at the imaging visit was synchronised with menopausal status at enrolment and was determined as follows: Post-menopausal (Post-MP) – were classified women with age at the imaging visit ≥58 years OR with bilateral oophorectomy at enrolment or at the imaging visit from Fields: [2834-0(2).0] "*Bilateral oophorectomy (both ovaries removed)*" Answer 1: "*Yes*" OR Fields [20004-0(2).0/31] "*Operation code (self-reported operation)*" code: 1355; OR with self-reported post-menopausal status at enrolment or at the imaging visit from Fields [2724-0(2).0] "*Had menopause*" Answer 1: "Yes" OR with age at the imaging visit ≥ 55 years when menopausal status was unknown at the imaging visit, i.e. they had not answered 0: "No" to Field [2724-2.0]; Pre-menopausal (Pre-MP) – were classified women who had not been defined as post-menopausal above and had reported pre-menopausal status at the imaging visit with Answer 0: "No" to Field [2724-2.0] OR had age at the imaging visit < 55 years when menopausal status at the imaging visit was unknown, i.e. not defined as post- or pre-menopausal according to the above criteria.

Menopausal status-HRT at enrolment or at the imaging visit – as the number of pre-menopausal women HRT users was limited, a combined variable was used in all analyses, separating only post-menopausal women by HRT use. Women were assigned to four categories: Pre-MP, Post-MP – Never HRT user, Post-MP – Former HRT user and Post-MP – Current HRT user.

Use of oral contraceptives at enrolment was determined for women by Field [2784-0.0] "*Ever taken oral contraceptive pill*"; Question: "*Have you ever taken the contraceptive pill? (include the 'mini-pill')*"; Answer 0: "*No*" (for Never user) or Answer 1: "Yes" (for Ever user). Further information was derived from Fields [6153-0.0/3]. Women providing Answer 5 "*Oral contraceptive pill or minipill*" were considered Ever user. Women with missing information for Field [2784-0.0] were assigned to the median category (Ever user).

Age at last live birth at enrolment was defined as follows: category No live births - was based on Field [2734-0.0] "*Number of live births*"; Question: "*How many children have you given birth to? (Please include live births only)*"; Answer (numerical) 0; categories < 30 years or ≥ 30 years – were based on Field [2764-0.0] Age at last live birth; Question: "*How old were you when you had your LAST child?*" or Field [3872-0.0] "*Age of primiparous women at birth of child*"; Question: "*How old were you when you had your child?*" (UK Biobank note: "Current Field was collected from women who indicated they had given birth to only one child, as defined by their answers to Field 2734"). Women with missing information for Field [2734-0.0] were assigned to the median category (<30 years).

Supplementary Table S1 Overlap between BMI-by-ABS-by-HI phenotypes defined with HI calculated with coefficients from UK Biobank or NHANES in men

Hip Index (UK Biobank)	Overlap: n (%)				BMI: mean (SD) **			
	Hip Index (NHANES)							
	Pear	Slim	Wide	Apple	Pear	Slim	Wide	Apple
**Normal weight BMI**								
**Pear**	12,958 (100)	-	-	-	23.1 (1.4)	-	-	-
**Slim**	4507 (33.0)	9159 (67.0)	-	-	22.9 (1.5)	23.5 (1.2)	-	-
**Wide**	-	-	15,092 (100)	-	-	-	23.1 (1.4)	-
**Apple**	-	-	3314 (40.0)	4962 (60.0)	-	-	22.9 (1.5)	23.6 (1.2)
**Overweight BMI**								
**Pear**	19,016 (94.7)	1055 (5.3)	-	-	27.2 (1.4)	28.9 (0.7)	-	-
**Slim**	1437 (4.7)	29,036 (95.3)	-	-	25.9 (0.7)	27.5 (1.4)	-	-
**Wide**	-	-	25,885 (95.4)	1246 (4.6)	-	-	27.3 (1.4)	28.9 (0.8)
**Apple**	-	-	1323 (6.0)	20,593 (94.0)	-	-	26.0 (0.7)	27.6 (1.4)
**Obese BMI**								
**Pear**	5817 (62.1)	3550 (37.9)	-	-	33.2 (3.1)	33.9 (3.1)	-	-
**Slim**	-	13,609 (100)	-	-	-	32.7 (2.5)	-	-
**Wide**	-	-	10,798 (69.5)	4728 (30.5)	-	-	33.4 (3.2)	34.1 (3.2)
**Apple**	-	-	-	12,204 (100)	-	-	-	33.0 (2.7)

ABSI – a body shape index (cut-off: ≥79.7998 exact median); **Apple** – large-ABSI-small-HI; **BMI** – body mass index; **HI** – hip index (cut-off: ≥49.11381 exact median for HI calculated with coefficients from UK Biobank (used for men in the current paper); ≥60.26223 exact median for HI calculated with coefficients from NHANES [Ref. 5] (the NHANES coefficients were used only for women in the current paper)); **n (%)** – number of participants (percent from the total per row); **NHANES** – National Health and Nutrition Examination Survey; **Pear** – small-ABSI-large-HI; **SD** – standard deviation; **Slim** – small-ABSI-small-HI; **Wide** – large-ABSI-large-HI; ** – BMI for correctly classified and for misclassified men was compared with t-test (per row), $p<10^{-16}$ for all comparisons.

Supplementary Table S2 Scaling coefficients for allometric body composition indices

		Scaling for weight and height #				Scaling for height ##		
**Set**	**Measurement**	**Mean (SD)**	**β Weight (kg)**	**γ Height (m)**	**R^2**	**Mean (SD)**	**δ Height (m)**	**R^2**
				**MEN**				
	Waist Circumference	78.9 (4.23)	2/3	-5/6		74.7 (8.37)	0.4112 (0.0236)	0.02
	Hip Circumference	48.3 (1.89)	2/5	-1/5		75.7 (5.26)	0.5079 (0.0146)	0.07
	Waist-to-Hip Ratio	4.09 (0.22)	1/4	-1/2		0.99 (0.07)	-0.0966 (0.0145)	<0.01
BIA	Total Fat-free Mass	2.92 (0.13)	0.5726 (0.0026)	0.9246 (0.0108)	0.85	20.7 (1.92)	1.9438 (0.0196)	0.39
BIA	Arms Fat-free Mass	1.25 (0.08)[a]	0.8066 (0.0039)	0.8456 (0.0162)	0.81	1.97 (0.26)	2.2813 (0.0281)	0.30
BIA	Legs Fat-free Mass	5.63 (0.28)[a]	0.7464 (0.0028)	0.4908 (0.0116)	0.87	7.24 (0.86)	1.8194 (0.0245)	0.26
BIA	Trunk Fat-free Mass	2.74 (0.14)	0.4205 (0.0029)	1.2000 (0.0118)	0.78	11.5 (0.88)	1.9484 (0.0164)	0.48
BIA	Total Fat Mass	4.31 (0.63)[c]	2.2763 (0.0089)	-2.7904 (0.0366)	0.81	10.7 (3.74)	1.2615 (0.0754)	0.02
BIA	Arms Fat Mass	1.51 (0.15)[d]	2.4811 (0.0056)	-2.6644 (0.0230)	0.93	0.77 (0.30)	1.7520 (0.0767)	0.03
BIA	Legs Fat Mass	1.09 (0.16)[c]	2.3169 (0.0083)	-2.9478 (0.0342)	0.84	3.14 (1.19)	1.1764 (0.0755)	0.02
BIA	Trunk Fat Mass	3.23 (0.57)[c]	2.2330 (0.0114)	-2.7582 (0.0469)	0.72	6.92 (2.36)	1.2167 (0.0788)	0.02
DXA	Total Lean Mass	3.88 (0.23)	0.4676 (0.0035)	1.0459 (0.0144)	0.71	19.2 (1.71)	1.8783 (0.0190)	0.39
DXA	Arms Lean Mass	3.05 (0.31)[a]	0.6007 (0.0059)	0.7856 (0.0243)	0.53	2.38 (0.32)	1.8548 (0.0282)	0.22
DXA	Legs Lean Mass	6.81 (0.50)[a]	0.6139 (0.0043)	1.0524 (0.0176)	0.72	5.56 (0.63)	2.1451 (0.0242)	0.34
DXA	Gynoid Lean Mass	5.19 (0.38)[a]	0.4893 (0.0044)	1.1381 (0.0180)	0.64	2.77 (0.28)	2.0091 (0.0218)	0.35
DXA	Trunk Lean Mass	2.72 (0.20)	0.3623 (0.0042)	1.1896 (0.0175)	0.57	9.40 (0.83)	1.8345 (0.0191)	0.37
DXA	Android Lean Mass	3.00 (0.26)[a]	0.4704 (0.0051)	0.9468 (0.0210)	0.53	1.50 (0.16)	1.7841 (0.0236)	0.27
DXA	Total Fat Mass	4.35 (0.64)[c]	2.2689 (0.0090)	-2.5364 (0.0370)	0.81	10.6 (3.65)	1.5023 (0.0753)	0.02
DXA	Arms Fat Mass	1.39 (0.24)[c]	1.9432 (0.0104)	-2.1162 (0.0427)	0.70	1.10 (0.37)	1.3428 (0.0695)	0.02
DXA	Legs Fat Mass	3.96 (0.73)[c]	1.8265 (0.0108)	-1.2989 (0.0443)	0.67	2.09 (0.70)	1.9524 (0.0674)	0.05
DXA	Gynoid Fat Mass	1.10 (0.18)[c]	2.0396 (0.0098)	-1.7454 (0.0403)	0.75	1.22 (0.41)	1.8852 (0.0708)	0.04
DXA	Trunk Fat Mass	6.79 (1.32)[d]	2.6863 (0.0123)	-3.4117 (0.0506)	0.76	6.96 (2.76)	1.3699 (0.0921)	0.01
DXA	Android Fat Mass	2.99 (0.74)[e]	3.1183 (0.0158)	-4.3694 (0.0652)	0.72	1.37 (0.62)	1.1811 (0.1098)	0.01
DXA	ASAT Mass	2.14 (0.71)[d]	2.1552 (0.0686)	-2.0038 (0.2832)	0.34	0.29 (0.13)	2.1964 (0.3044)	0.03
DXA	VAT Mass	2.15 (0.81)[f]	3.8241 (0.0700)	-6.1126 (0.2892)	0.60	0.79 (0.44)	1.3401 (0.4007)	0.01
MRI	ASAT+VAT Volume	3.63 (0.74)[d]	2.8046 (0.0259)	-3.8337 (0.1071)	0.75	5.50 (2.23)	1.1896 (0.1915)	0.01
MRI	ASAT Volume	3.61 (0.74)[d]	2.5834 (0.0247)	-3.1548 (0.1020)	0.74	2.56 (1.07)	1.4725 (0.1780)	0.02
MRI	VAT Volume	6.89 (2.23)[e]	3.1115 (0.0406)	-4.7065 (0.1680)	0.60	2.99 (1.40)	0.8667 (0.2379)	<0.01
	Hand Grip Strength	11.0 (2.21)	0.0860 (0.0123)	1.6180 (0.0504)	0.09	14.8 (2.97)	1.7711 (0.0455)	0.09
				**WOMEN**				
	Waist Circumference	73.8 (5.25)	2/3	-5/6		77.0 (10.7)	0.1482 (0.0278)	<0.01
	Hip Circumference	63.8 (2.60)	0.482	-0.310		86.5 (8.03)	0.3207 (0.0185)	0.02
	Waist-to-Hip Ratio	3.62 (0.27)	1/4	-1/2		0.89 (0.07)	-0.1725 (0.0170)	0.01
BIA	Total Fat-free Mass	4.42 (0.22)	0.4573 (0.0023)	0.7235 (0.0106)	0.78	22.1 (2.03)	1.3910 (0.0183)	0.26
BIA	Arms Fat-free Mass	2.56 (0.15)[a]	0.5945 (0.0028)	0.7472 (0.0129)	0.79	2.08 (0.24)	1.6152 (0.0234)	0.22
BIA	Legs Fat-free Mass	1.02 (0.05)	0.5498 (0.0025)	0.6348 (0.0112)	0.81	7.08 (0.76)	1.4375 (0.0213)	0.22
BIA	Trunk Fat-free Mass	3.45 (0.19)	0.3772 (0.0026)	0.7725 (0.0119)	0.68	13.0 (1.09)	1.3231 (0.0170)	0.27
BIA	Total Fat Mass	1.14 (0.11)[b]	1.9592 (0.0049)	-1.2442 (0.0222)	0.91	11.7 (4.00)	1.6161 (0.0690)	0.03
BIA	Arms Fat Mass	9.17 (0.99)[e]	2.8150 (0.0056)	-3.6112 (0.0256)	0.94	2.01 (1.01)	0.4983 (0.0975)	<0.01
BIA	Legs Fat Mass	2.13 (0.11)[b]	1.6654 (0.0026)	-1.8597 (0.0119)	0.96	7.70 (2.27)	0.5715 (0.0570)	0.01
BIA	Trunk Fat Mass	2.51 (0.38)[c]	2.0444 (0.0078)	-0.2801 (0.0354)	0.82	3.48 (1.24)	2.7044 (0.0763)	0.07
DXA	Total Lean Mass	4.04 (0.25)	0.4022 (0.0030)	1.1957 (0.0138)	0.69	16.6 (1.54)	1.7828 (0.0188)	0.35
DXA	Arms Lean Mass	2.67 (0.27)[a]	0.5347 (0.0049)	0.9817 (0.0223)	0.53	1.75 (0.24)	1.7623 (0.0277)	0.20
DXA	Legs Lean Mass	6.11 (0.47)[a]	0.5837 (0.0037)	1.2315 (0.0169)	0.72	4.77 (0.60)	2.0836 (0.0253)	0.29
DXA	Gynoid Lean Mass	5.35 (0.38)[a]	0.4282 (0.0035)	1.2902 (0.0161)	0.65	2.41 (0.25)	1.9154 (0.0209)	0.34
DXA	Trunk Lean Mass	3.02 (0.23)	0.2881 (0.0036)	1.3242 (0.0166)	0.53	8.32 (0.75)	1.7449 (0.0184)	0.35
DXA	Android Lean Mass	2.72 (0.25)[a]	0.4111 (0.0044)	1.3036 (0.0200)	0.53	1.15 (0.13)	1.9037 (0.0234)	0.28
DXA	Total Fat Mass	1.79 (0.19)[b]	1.9361 (0.0054)	-1.8969 (0.0248)	0.89	17.0 (5.75)	0.9296 (0.0691)	0.01
DXA	Arms Fat Mass	3.85 (0.61)[c]	1.7583 (0.0077)	-1.7167 (0.0352)	0.76	1.94 (0.66)	0.8502 (0.0678)	0.01
DXA	Legs Fat Mass	1.91 (0.32)[b]	1.5486 (0.0082)	-0.8302 (0.0374)	0.69	4.54 (1.45)	1.4306 (0.0629)	0.03
DXA	Gynoid Fat Mass	7.99 (1.13)[c]	1.6067 (0.0070)	-0.9431 (0.0320)	0.77	2.34 (0.72)	1.4025 (0.0618)	0.03
DXA	Trunk Fat Mass	2.32 (0.41)[c]	2.3695 (0.0092)	-2.8509 (0.0418)	0.80	10.3 (4.27)	0.6082 (0.0888)	<0.01
DXA	Android Fat Mass	5.26 (1.28)[e]	2.8976 (0.0127)	-3.4911 (0.0578)	0.76	1.53 (0.78)	0.7390 (0.1115)	<0.01
DXA	ASAT Mass	8.75 (2.04)[e]	2.5002 (0.0361)	-1.9287 (0.1598)	0.69	0.67 (0.30)	1.5570 (0.2706)	0.01
DXA	VAT Mass	1.21 (0.65)[f]	3.9525 (0.0823)	-7.2106 (0.3644)	0.51	1.76 (1.29)	-1.7000 (0.4950)	0.01
MRI	ASAT+VAT Volume	1.63 (0.31)[c]	2.4455 (0.0190)	-3.3274 (0.0858)	0.79	9.93 (4.21)	0.1470 (0.1779)	<0.01
MRI	ASAT Volume	1.71 (0.31)[c]	2.3334 (0.0179)	-3.0151 (0.0808)	0.80	6.96 (2.86)	0.3001 (0.1693)	<0.01
MRI	VAT Volume	1.28 (0.48)[d]	2.8320 (0.0365)	-4.3925 (0.1651)	0.58	3.13 (1.75)	-0.3690 (0.2410)	<0.01
	Hand Grip Strength	8.26 (1.88)	0.0137 (0.0121)	2.1227 (0.0553)	0.09	8.67 (1.97)	2.1428 (0.0523)	0.09

ASAT – abdominal subcutaneous adipose tissue; **BIA** – bioelectrical impedance analysis; **DXA** – dual-emission X-ray absorptiometry; **MRI** – magnetic resonance imaging; $\mathbf{R^2}$ – proportion explained variability; **SD** – standard deviation; **VAT** – visceral adipose tissue.

[a] – values multiplied by 10; [b] – values multiplied by 100, [c] – values multiplied by 1,000; [d] – values multiplied by 10,000; [e] – values multiplied by 100,000; [f] – values multiplied by 1,000,000.

[#] – a sex-specific model regressing:

$$\log(\text{Measurement}) \sim \beta^*\log(\text{Weight}) + \gamma^*\log(\text{Height})$$

the index was derived as:

$$\text{Index} = \text{Measurement} * \text{Weight}^{-\beta} * \text{Height}^{-\gamma}$$

Note that waist circumference, hip circumference and waist-to-hip ratio scaled for weight and height correspond to A Body Shape Index (ABSI), hip index (HI) and waist-to-hip index (WHI).

[##] – a sex-specific model regressing:

$$\log(\text{Measurement}) \sim \delta^*\log(\text{Height})$$

the index was derived as:

$$\text{Index} = \text{Measurement} * \text{Height}^{-\delta}$$

BIA measurements were provided in kg, DXA measurements were provided in g and were divided by 1000 to convert to kg; MRI measurements were provided in L. Models for anthropometric, BIA and DXA measurements and hand grip strength were based on the DXA dataset (except for VAT and ASAT, which were based on the DXA VAT subset) and for MRI on the MRI dataset (see Supplementary Figure S1 for the definition of datasets).

Weight was obtained in kg from Field [21002-0.0] for the cancer risk dataset and Field [21002-2.0] for the imaging datasets and was used in kg. Height was obtained in cm from Fields [50-0(2).0] “Standing height” and was used in cm for HI and WHI but was divided by 100 and used in m for body composition indices and ABSI. Waist circumference was obtained in cm from Fields [48-0(2).0] and was used in cm for WHI and indices scaled only for height but was multiplied by 10 and used in mm for ABSI. Hip circumference was obtained in cm from Fields [49-0(2).0] and was used in cm.

Note that the regression coefficients β, γ and δ provided in the Table include the corresponding sign, as derived from the log-linear models, and they were multiplied by -1 in the Index formula. This change of sign is required because Weight and Height are part of the denominator in the Index equation, while the Index formula is set up only with multiplication. The multiplication by -1 is incorporated in the formulae for ABSI, HI and WHI in the main document.

Supplementary Table S3 Characteristics of study participants according to ABSI-by-HI body-shape phenotype

	MEN						WOMEN					
**Cancer risk dataset**	**Overall**	**Pear**	**Slim**	**Wide**	**Apple**	**P**	**Overall**	**Pear**	**Slim**	**Wide**	**Apple**	**P**
Cohort size: n (%)	200,289	47,515 (23.7)	56,606 (28.3)	58,621 (29.3)	37,547 (18.7)		230,326	55,366 (24.0)	49,149 (21.3)	70,934 (30.8)	54,877 (23.8)	
Anthropometry: mean (SD)												
Weight (kg)	86.1 (13.8)	84.8 (13.9)	85.4 (12.8)	86.6 (14.5)	87.7 (13.6)	<0.001	71.2 (13.1)	69.4 (13.0)	69.5 (12.1)	72.5 (14.1)	72.9 (12.4)	<0.001
Height (cm)	175.9 (6.8)	175.9 (6.7)	175.5 (6.6)	176.0 (6.9)	176.0 (6.7)	<0.001	162.6 (6.2)	162.6 (6.2)	162.7 (6.2)	162.4 (6.3)	162.9 (6.2)	<0.001
Waist circumference (cm)	96.9 (11)	92.7 (10.0)	92.4 (9.1)	101.5 (10.8)	101.9 (9.9)	<0.001	84.4 (12)	78.2 (9.6)	77.9 (9.1)	89.6 (12.1)	89.6 (10.4)	<0.001
Hip circumference (cm)	103.4 (7.2)	105.1 (7.0)	100.1 (6.0)	106.5 (7.5)	101.6 (6.1)	<0.001	103.2 (9.7)	104.5 (9.5)	98.5 (8.0)	107.0 (10.5)	101.0 (7.8)	<0.001
Waist-to-hip ratio	0.94 (0.06)	0.88 (0.05)	0.92 (0.05)	0.95 (0.05)	1.00 (0.05)	<0.001	0.82 (0.07)	0.75 (0.04)	0.79 (0.04)	0.84 (0.05)	0.88 (0.05)	<0.001
Weight change: n (%)												
Weight loss	29,050 (14.5)	7103 (14.9)	9094 (16.1)	7545 (12.9)	5308 (14.1)	<0.001	34,959 (15.2)	7515 (13.6)	8100 (16.5)	10,241 (14.4)	9103 (16.6)	<0.001
Stable weight	123,137 (61.5)	30,339 (63.9)	35,148 (62.1)	35,671 (60.9)	21,979 (58.5)		117,071 (50.8)	29,473 (53.2)	25,817 (52.5)	35,073 (49.4)	26,708 (48.7)	
Weight gain	44,687 (22.3)	9,352 (19.7)	11,498 (20.3)	14,284 (24.4)	9553 (25.4)		74,689 (32.4)	17,574 (31.7)	14,530 (29.6)	24,391 (34.4)	18,194 (33.2)	
Missing	3415 (1.7)	721 (1.5)	866 (1.5)	1121 (1.9)	707 (1.9)		3607 (1.6)	804 (1.5)	702 (1.4)	1229 (1.7)	872 (1.6)	
Smoking status: n (%)												
Never smoked	68,545 (34.2)	18,300 (38.5)	20,614 (36.4)	18,672 (31.9)	10,959 (29.2)	<0.001	100,586 (43.7)	26,716 (48.3)	21,840 (44.4)	30,451 (42.9)	21,579 (39.3)	<0.001
Former occasional smoker	50,609 (25.3)	13,416 (28.2)	14,543 (25.7)	14,373 (24.5)	8277 (22.0)		63,068 (27.4)	15,846 (28.6)	13,641 (27.8)	19,357 (27.3)	14,224 (25.9)	
Former regular smoker	55,968 (27.9)	11,058 (23.3)	14,379 (25.4)	18,165 (31.0)	12,366 (32.9)		45,621 (19.8)	9248 (16.7)	9172 (18.7)	14,671 (20.7)	12,530 (22.8)	
Current smoker	24,504 (12.2)	4626 (9.7)	6896 (12.2)	7177 (12.2)	5805 (15.5)		20,317 (8.8)	3407 (6.2)	4369 (8.9)	6219 (8.8)	6322 (11.5)	
Missing	663 (0.3)	115 (0.2)	174 (0.3)	234 (0.4)	140 (0.4)		734 (0.3)	149 (0.3)	127 (0.3)	236 (0.3)	222 (0.4)	
Alcohol intake: n (%)												
Up to three times a month	40,795 (20.4)	9246 (19.5)	11,247 (19.9)	12,232 (20.9)	8070 (21.5)	<0.001	80,744 (35.1)	18,756 (33.9)	16,478 (33.5)	26,032 (36.7)	19,478 (35.5)	<0.001
Up to four times a week	106,892 (53.4)	26,479 (55.7)	32,031 (56.6)	29,663 (50.6)	18,719 (49.9)		110,897 (48.1)	27,843 (50.3)	24,720 (50.3)	32,799 (46.2)	25,535 (46.5)	
Daily	52,445 (26.2)	11,755 (24.7)	13,299 (23.5)	16,675 (28.4)	10,716 (28.5)		38,540 (16.7)	8746 (15.8)	7926 (16.1)	12,049 (17.0)	9819 (17.9)	
Missing	157 (0.1)	35 (0.1)	29 (0.1)	51 (0.1)	42 (0.1)		145 (0.1)	21 (0.0)	25 (0.1)	54 (0.1)	45 (0.1)	
Physical activity: n (%)												
Inactive	30,530 (15.2)	5810 (12.2)	6594 (11.6)	11,196 (19.1)	6930 (18.5)	<0.001	38,492 (16.7)	8729 (15.8)	6868 (14.0)	13,430 (18.9)	9465 (17.2)	<0.001
Moderately active	89,789 (44.8)	20,385 (42.9)	23,037 (40.7)	28,620 (48.8)	17,747 (47.3)		120,059 (52.1)	28,604 (51.7)	24,258 (49.4)	38,256 (53.9)	28,941 (52.7)	
Active	79,323 (39.6)	21,220 (44.7)	26,828 (47.4)	18,567 (31.7)	12,708 (33.8)		70,936 (30.8)	17,889 (32.3)	17,908 (36.4)	18,906 (26.7)	16,233 (29.6)	
Missing	647 (0.3)	100 (0.2)	147 (0.3)	238 (0.4)	162 (0.4)		839 (0.4)	144 (0.3)	115 (0.2)	342 (0.5)	238 (0.4)	
Townsend index: n (%)												
Lowest tertile	66,682 (33.3)	16,850 (35.5)	19,014 (33.6)	19,302 (32.9)	11,516 (30.7)	<0.001	76,762 (33.3)	19,731 (35.6)	17,125 (34.8)	22,791 (32.1)	17,115 (31.2)	<0.001
Middle tertile	66,683 (33.3)	15,993 (33.7)	18,902 (33.4)	19,384 (33.1)	12,404 (33.0)		76,623 (33.3)	18,774 (33.9)	16,449 (33.5)	23,319 (32.9)	18,081 (32.9)	
Highest tertile	66,681 (33.3)	14,609 (30.7)	18,613 (32.9)	19,874 (33.9)	13,585 (36.2)		76,686 (33.3)	16,793 (30.3)	15,515 (31.6)	24,754 (34.9)	19,624 (35.8)	
Missing	243 (0.1)	63 (0.1)	77 (0.1)	61 (0.1)	42 (0.1)		255 (0.1)	68 (0.1)	60 (0.1)	70 (0.1)	57 (0.1)	
Family history: n (%)												
No cancer	130,121 (65.0)	31,324 (65.9)	37,191 (65.7)	37,537 (64.0)	24,069 (64.1)	<0.001	147,988 (64.3)	36,070 (65.1)	31,916 (64.9)	45,092 (63.6)	34,910 (63.6)	<0.001
Lung, breast, prostate cancer	54,232 (27.1)	12,534 (26.4)	15,029 (26.6)	16,280 (27.8)	10,389 (27.7)		64,979 (28.2)	15,255 (27.6)	13,581 (27.6)	20,393 (28.7)	15,750 (28.7)	
Bowel cancer	15,936 (8.0)	3657 (7.7)	4386 (7.7)	4804 (8.2)	3089 (8.2)		17,359 (7.5)	4041 (7.3)	3652 (7.4)	5449 (7.7)	4217 (7.7)	
Fruit & vegetable intake: n (%)												
< five portions a day	133,561 (66.7)	30,999 (65.2)	36,664 (64.8)	40,160 (68.5)	25,738 (68.5)	<0.001	122,894 (53.4)	29,265 (52.9)	24,994 (50.9)	39,034 (55.0)	29,601 (53.9)	<0.001
≥ five portions a day	63,329 (31.6)	15,910 (33.5)	19,032 (33.6)	17,357 (29.6)	11,030 (29.4)		105,014 (45.6)	25,603 (46.2)	23,716 (48.3)	31,065 (43.8)	24,630 (44.9)	
Missing	3399 (1.7)	606 (1.3)	910 (1.6)	1104 (1.9)	779 (2.1)		2418 (1.0)	498 (0.9)	439 (0.9)	835 (1.2)	646 (1.2)	

	MEN						WOMEN					
**Cancer risk dataset**	**Overall**	**Pear**	**Slim**	**Wide**	**Apple**	**P**	**Overall**	**Pear**	**Slim**	**Wide**	**Apple**	**P**
Processed meat intake: n (%)												
< two times a week	111,678 (55.8)	27,911 (58.7)	32,492 (57.4)	31,771 (54.2)	19,504 (51.9)	<0.001	181,903 (79.0)	44,460 (80.3)	39,819 (81.0)	54,953 (77.5)	42,671 (77.8)	<0.001
≥ two times a week	88,300 (44.1)	19,551 (41.1)	24,027 (42.4)	26,751 (45.6)	17,971 (47.9)		48,114 (20.9)	10,846 (19.6)	9276 (18.9)	15,875 (22.4)	12,117 (22.1)	
Missing	311 (0.2)	53 (0.1)	87 (0.2)	99 (0.2)	72 (0.2)		309 (0.1)	60 (0.1)	54 (0.1)	106 (0.1)	89 (0.2)	
Red meat intake: n (%)												
< two times a week	88,953 (44.4)	22,022 (46.3)	25,641 (45.3)	25,427 (43.4)	15,863 (42.2)	<0.001	121,203 (52.6)	30,177 (54.5)	26,640 (54.2)	36,401 (51.3)	27,985 (51.0)	<0.001
≥ two times a week	109,271 (54.6)	25,133 (52.9)	30,421 (53.7)	32,539 (55.5)	21,178 (56.4)		107,142 (46.5)	24,805 (44.8)	22,152 (45.1)	33,818 (47.7)	26,367 (48.0)	
Missing	2,065 (1.0)	360 (0.8)	544 (1.0)	655 (1.1)	506 (1.3)		1981 (0.9)	384 (0.7)	357 (0.7)	715 (1.0)	525 (1.0)	
Fibre intake: n (%)												
Lowest tertile	63,432 (31.7)	14,075 (29.6)	17,654 (31.2)	18,930 (32.3)	12,773 (34.0)	<0.001	73,665 (32.0)	17,250 (31.2)	15,306 (31.1)	23,078 (32.5)	18,031 (32.9)	<0.001
Middle tertile	63,444 (31.7)	15,472 (32.6)	17,938 (31.7)	18,421 (31.4)	11,613 (30.9)		73,420 (31.9)	18,032 (32.6)	15,753 (32.1)	22,232 (31.3)	17,403 (31.7)	
Highest tertile	63,418 (31.7)	15,898 (33.5)	18,543 (32.8)	17,919 (30.6)	11,058 (29.5)		73,522 (31.9)	17,922 (32.4)	16,403 (33.4)	22,082 (31.1)	17,115 (31.2)	
Missing	9995 (5.0)	2070 (4.4)	2471 (4.4)	3351 (5.7)	2103 (5.6)		9719 (4.2)	2162 (3.9)	1687 (3.4)	3542 (5.0)	2328 (4.2)	
Aspirin or Ibuprofen: n (%)												
No	139,676 (69.7)	34,796 (73.2)	40,611 (71.7)	39,628 (67.6)	24,641 (65.6)	<0.001	168,097 (73.0)	41,141 (74.3)	36,491 (74.2)	51,048 (72.0)	39,417 (71.8)	<0.001
Yes	56,844 (28.4)	11,861 (25.0)	15,226 (26.9)	17,570 (30.0)	12,187 (32.5)		58,507 (25.4)	13,326 (24.1)	12,089 (24.6)	18,384 (25.9)	14,708 (26.8)	
Missing	3,769 (1.9)	858 (1.8)	769 (1.4)	1423 (2.4)	719 (1.9)		3722 (1.6)	899 (1.6)	569 (1.2)	1502 (2.1)	752 (1.4)	
Assessment region: n (%)												
London	22,815 (11.4)	5259 (11.1)	6253 (11.0)	6594 (11.2)	4709 (12.5)	<0.001	27,287 (11.8)	6324 (11.4)	5642 (11.5)	8297 (11.7)	7024 (12.8)	<0.001
North-West	32,609 (16.3)	7386 (15.5)	11,306 (20.0)	7646 (13.0)	6271 (16.7)		35,893 (15.6)	7928 (14.3)	9441 (19.2)	9459 (13.3)	9065 (16.5)	
North-East	24,410 (12.2)	5546 (11.7)	8484 (15.0)	6219 (10.6)	4161 (11.1)		28,097 (12.2)	6737 (12.2)	6433 (13.1)	7906 (11.1)	7021 (12.8)	
Yorkshire and Humber	30,467 (15.2)	6839 (14.4)	7743 (13.7)	9516 (16.2)	6369 (17.0)		35,144 (15.3)	9103 (16.4)	6634 (13.5)	11,135 (15.7)	8272 (15.1)	
West Midlands	18,633 (9.3)	3449 (7.3)	5768 (10.2)	5736 (9.8)	3680 (9.8)		18,914 (8.2)	3588 (6.5)	5022 (10.2)	5872 (8.3)	4432 (8.1)	
East Midlands	13,856 (6.9)	3667 (7.7)	2719 (4.8)	4795 (8.2)	2675 (7.1)		15,917 (6.9)	4276 (7.7)	1839 (3.7)	5724 (8.1)	4078 (7.4)	
South-East	17,220 (8.6)	4865 (10.2)	2345 (4.1)	7110 (12.1)	2900 (7.7)		20,740 (9.0)	5631 (10.2)	2183 (4.4)	8732 (12.3)	4194 (7.6)	
South-West	17,249 (8.6)	4668 (9.8)	4567 (8.1)	4860 (8.3)	3154 (8.4)		20,955 (9.1)	5349 (9.7)	4743 (9.7)	6063 (8.5)	4800 (8.7)	
Wales	8513 (4.3)	2033 (4.3)	2245 (4.0)	2787 (4.8)	1448 (3.9)		9764 (4.2)	2146 (3.9)	2017 (4.1)	3397 (4.8)	2204 (4.0)	
Scotland	14,517 (7.2)	3803 (8.0)	5176 (9.1)	3358 (5.7)	2180 (5.8)		17,615 (7.6)	4284 (7.7)	5195 (10.6)	4349 (6.1)	3787 (6.9)	
MP status & HRT use: n (%)												
Pre-MP							67,106 (29.1)	19,460 (35.1)	18,132 (36.9)	15,924 (22.4)	13,590 (24.8)	<0.001
Post-MP Never user							77,841 (33.8)	17,612 (31.8)	15,200 (30.9)	25,766 (36.3)	19,263 (35.1)	
Post-MP Former user							69,624 (30.2)	14,456 (26.1)	12,340 (25.1)	24,587 (34.7)	18,241 (33.2)	
Post-MP Current user							15,394 (6.7)	3782 (6.8)	3419 (7.0)	4512 (6.4)	3681 (6.7)	
Post-MP Missing							361 (0.2)	56 (0.1)	58 (0.1)	145 (0.2)	102 (0.2)	
Oral contraceptives: n (%)												
Never user							40,251 (17.5)	8907 (16.1)	7505 (15.3)	13,903 (19.6)	9936 (18.1)	<0.001
Ever user							189,631 (82.3)	46,347 (83.7)	41,573 (84.6)	56,868 (80.2)	44,843 (81.7)	
Missing							444 (0.2)	112 (0.2)	71 (0.1)	163 (0.2)	98 (0.2)	
Age at last pregnancy: n (%)												
No live births							42,629 (18.5)	11,611 (21.0)	9880 (20.1)	12,173 (17.2)	8965 (16.3)	<0.001
< 30 years							89,363 (38.8)	20,373 (36.8)	18,421 (37.5)	28,341 (40.0)	22,228 (40.5)	
≥ 30 years							97,781 (42.5)	23,292 (42.1)	20,768 (42.3)	30,194 (42.6)	23,527 (42.9)	
Missing							553 (0.2)	90 (0.2)	80 (0.2)	226 (0.3)	157 (0.3)	

	MEN						WOMEN					
**DXA dataset**	**Overall**	**Pear**	**Slim**	**Wide**	**Apple**	**P**	**Overall**	**Pear**	**Slim**	**Wide**	**Apple**	**P**
Cohort size: n (%)	15,520	2515 (16.2)	6951 (44.8)	3060 (19.7)	2994 (19.3)		16,548	3175 (19.2)	4408 (26.6)	4590 (27.7)	4375 (26.4)	
Anthropometry: mean (SD)												
Weight (kg)	84.4 (13.4)	83.8 (14.2)	83.9 (12.7)	84.9 (14.5)	85.6 (13.2)	<0.001	69.4 (12.5)	68.5 (12.8)	67.8 (11.6)	70.1 (13.4)	70.9 (12.1)	<0.001
Height (cm)	176.4 (6.6)	176.2 (6.7)	176.2 (6.5)	176.5 (6.8)	176.7 (6.6)	0.003	162.9 (6.2)	162.6 (6.1)	162.9 (6.1)	162.4 (6.1)	163.4 (6.3)	<0.001
Waist circumference (cm)	94.3 (10.7)	91.6 (10.0)	90.5 (9.0)	99.8 (10.8)	99.7 (9.8)	<0.001	82.7 (11.5)	77.3 (9.5)	76.3 (8.8)	88.0 (11.4)	87.6 (10.1)	<0.001
Hip circumference (cm)	101 (7.3)	104.3 (7.1)	98.3 (6.2)	105.5 (7.6)	99.9 (6.1)	<0.001	101.1 (9.5)	103.8 (9.5)	96.8 (8.0)	105.3 (9.8)	99.2 (8.0)	<0.001
Waist-to-hip ratio	0.93 (0.06)	0.88 (0.05)	0.92 (0.05)	0.94 (0.05)	1.00 (0.05)	<0.001	0.82 (0.07)	0.74 (0.04)	0.79 (0.04)	0.83 (0.05)	0.88 (0.05)	<0.001
Weight change: n (%)												
Weight loss	2976 (19.2)	488 (19.4)	1405 (20.2)	520 (17.0)	563 (18.8)	<0.001	2869 (17.3)	490 (15.4)	786 (17.8)	772 (16.8)	821 (18.8)	<0.001
Stable weight	9777 (63.0)	1599 (63.6)	4384 (63.1)	1940 (63.4)	1854 (61.9)		9047 (54.7)	1804 (56.8)	2507 (56.9)	2507 (54.6)	2229 (50.9)	
Weight gain	2541 (16.4)	399 (15.9)	1060 (15.2)	549 (17.9)	533 (17.8)		4344 (26.3)	822 (25.9)	1043 (23.7)	1236 (26.9)	1243 (28.4)	
Missing	226 (1.5)	29 (1.2)	102 (1.5)	51 (1.7)	44 (1.5)		288 (1.7)	59 (1.9)	72 (1.6)	75 (1.6)	82 (1.9)	
Smoking status: n (%)												
Never smoked	6602 (42.5)	1111 (44.2)	3044 (43.8)	1278 (41.8)	1169 (39.0)	<0.001	8176 (49.4)	1660 (52.3)	2204 (50.0)	2261 (49.3)	2051 (46.9)	<0.001
Former occasional smoker	4167 (26.8)	721 (28.7)	1896 (27.3)	783 (25.6)	767 (25.6)		4636 (28.0)	937 (29.5)	1220 (27.7)	1277 (27.8)	1202 (27.5)	
Former regular smoker	3992 (25.7)	570 (22.7)	1674 (24.1)	858 (28.0)	890 (29.7)		3088 (18.7)	502 (15.8)	800 (18.1)	875 (19.1)	911 (20.8)	
Current smoker	668 (4.3)	102 (4.1)	302 (4.3)	117 (3.8)	147 (4.9)		508 (3.1)	58 (1.8)	147 (3.3)	135 (2.9)	168 (3.8)	
Missing	91 (0.6)	11 (0.4)	35 (0.5)	24 (0.8)	21 (0.7)		140 (0.8)	18 (0.6)	37 (0.8)	42 (0.9)	43 (1.0)	
Alcohol intake: n (%)												
Up to three times a month	3155 (20.3)	498 (19.8)	1388 (20.0)	647 (21.1)	622 (20.8)	<0.001	5442 (32.9)	1110 (35.0)	1393 (31.6)	1547 (33.7)	1392 (31.8)	<0.001
Up to four times a week	9108 (58.7)	1530 (60.8)	4182 (60.2)	1705 (55.7)	1691 (56.5)		8808 (53.2)	1699 (53.5)	2417 (54.8)	2400 (52.3)	2292 (52.4)	
Daily	3190 (20.6)	478 (19.0)	1357 (19.5)	689 (22.5)	666 (22.2)		2207 (13.3)	350 (11.0)	572 (13.0)	617 (13.4)	668 (15.3)	
Missing	67 (0.4)	9 (0.4)	24 (0.3)	19 (0.6)	15 (0.5)		91 (0.5)	16 (0.5)	26 (0.6)	26 (0.6)	23 (0.5)	
Physical activity: n (%)												
Inactive	1619 (10.4)	223 (8.9)	584 (8.4)	416 (13.6)	396 (13.2)	<0.001	1830 (11.1)	354 (11.1)	387 (8.8)	577 (12.6)	512 (11.7)	<0.001
Moderately active	7380 (47.6)	1148 (45.6)	3106 (44.7)	1585 (51.8)	1541 (51.5)		9143 (55.3)	1739 (54.8)	2331 (52.9)	2647 (57.7)	2426 (55.5)	
Active	6453 (41.6)	1133 (45.0)	3239 (46.6)	1040 (34.0)	1041 (34.8)		5472 (33.1)	1065 (33.5)	1662 (37.7)	1333 (29.0)	1412 (32.3)	
Missing	68 (0.4)	11 (0.4)	22 (0.3)	19 (0.6)	16 (0.5)		103 (0.6)	17 (0.5)	28 (0.6)	33 (0.7)	25 (0.6)	
Townsend index: n (%)												
Lowest tertile	5169 (33.3)	875 (34.8)	2317 (33.3)	1049 (34.3)	928 (31.0)	0.066	5,513 (33.3)	1094 (34.5)	1506 (34.2)	1496 (32.6)	1417 (32.4)	0.033
Middle tertile	5167 (33.3)	839 (33.4)	2279 (32.8)	1037 (33.9)	1012 (33.8)		5,508 (33.3)	1101 (34.7)	1427 (32.4)	1549 (33.7)	1431 (32.7)	
Highest tertile	5168 (33.3)	799 (31.8)	2347 (33.8)	971 (31.7)	1051 (35.1)		5,510 (33.3)	976 (30.7)	1472 (33.4)	1539 (33.5)	1523 (34.8)	
Missing	16 (0.1)	2 (0.1)	8 (0.1)	3 (0.1)	3 (0.1)		17 (0.1)	4 (0.1)	3 (0.1)	6 (0.1)	4 (0.1)	
Assessment Region: n (%)												
North-West	10,931 (70.4)	1927 (76.6)	5369 (77.2)	1784 (58.3)	1851 (61.8)	<0.001	11,386 (68.8)	2488 (78.4)	3,282 (74.5)	2652 (57.8)	2,964 (67.7)	<0.001
North-East	3035 (19.6)	421 (16.7)	641 (9.2)	1124 (36.7)	849 (28.4)		3401 (20.6)	496 (15.6)	357 (8.1)	1649 (35.9)	899 (20.5)	
South	1554 (10.0)	167 (6.6)	941 (13.5)	152 (5.0)	294 (9.8)		1761 (10.6)	191 (6.0)	769 (17.4)	289 (6.3)	512 (11.7)	
MP status & HRT use: n (%)												
Pre-MP							1472 (8.9)	311 (9.8)	563 (12.8)	265 (5.8)	333 (7.6)	<0.001
Post-MP Never user							9050 (54.7)	1755 (55.3)	2379 (54.0)	2498 (54.4)	2418 (55.3)	
Post-MP Former user							4760 (28.8)	848 (26.7)	1088 (24.7)	1529 (33.3)	1295 (29.6)	
Post-MP Current user							1145 (6.9)	240 (7.6)	342 (7.8)	265 (5.8)	298 (6.8)	
Post-MP Missing							121 (0.7)	21 (0.7)	36 (0.8)	33 (0.7)	31 (0.7)	

	MEN						WOMEN					
**MRI dataset**	**Overall**	**Pear**	**Slim**	**Wide**	**Apple**	**P**	**Overall**	**Pear**	**Slim**	**Wide**	**Apple**	**P**
Cohort size: n (%)	3997	886 (22.2)	1857 (46.5)	780 (19.5)	474 (11.9)		4402	1077 (24.5)	1253 (28.5)	1007 (22.9)	1065 (24.2)	
Anthropometry: mean (SD)												
Weight (kg)	84.1 (13.1)	83.7 (13.6)	84.5 (12.7)	84.0 (13.8)	83.8 (12.1)	0.4	69.4 (12.4)	68.0 (12.7)	68.4 (11.9)	70.9 (13.4)	70.6 (11.3)	<0.001
Height (cm)	176.1 (6.4)	175.8 (6.7)	175.8 (6.3)	176.9 (6.6)	176.3 (6.2)	<0.001	162.8 (6.2)	162.6 (6.2)	162.8 (6.0)	162.7 (6.0)	163.2 (6.4)	0.065
Waist circumference (cm)	93.3 (9.8)	91.6 (9.6)	91.0 (8.8)	98.1 (10.1)	98.0 (9.1)	<0.001	81.8 (10.9)	76.9 (9.4)	77.2 (8.8)	87.3 (11.3)	86.9 (9.3)	<0.001
Hip circumference (cm)	101.5 (6.9)	104.3 (6.7)	99.2 (6.0)	105.0 (7.3)	99.7 (5.6)	<0.001	101.2 (9.3)	103.4 (9.3)	97.5 (8.0)	105.5 (9.9)	99.2 (7.5)	<0.001
Waist-to-hip ratio	0.92 (0.06)	0.88 (0.05)	0.92 (0.05)	0.93 (0.04)	0.98 (0.05)	<0.001	0.81 (0.06)	0.74 (0.04)	0.79 (0.04)	0.83 (0.04)	0.87 (0.05)	<0.001
Weight change: n (%)												
Weight loss	729 (18.2)	159 (17.9)	364 (19.6)	141 (18.1)	65 (13.7)	0.07	760 (17.3)	170 (15.8)	237 (18.9)	158 (15.7)	195 (18.3)	0.192
Stable weight	2525 (63.2)	566 (63.9)	1152 (62.0)	494 (63.3)	313 (66.0)		2372 (53.9)	607 (56.4)	675 (53.9)	550 (54.6)	540 (50.7)	
Weight gain	680 (17.0)	154 (17.4)	308 (16.6)	128 (16.4)	90 (19.0)		1194 (27.1)	283 (26.3)	321 (25.6)	280 (27.8)	310 (29.1)	
Missing	63 (1.6)	7 (0.8)	33 (1.8)	17 (2.2)	6 (1.3)		76 (1.7)	17 (1.6)	20 (1.6)	19 (1.9)	20 (1.9)	
Smoking status: n (%)												
Never smoked	1654 (41.4)	374 (42.2)	804 (43.3)	297 (38.1)	179 (37.8)	<0.001	2174 (49.4)	544 (50.5)	636 (50.8)	491 (48.8)	503 (47.2)	0.082
Former occasional smoker	1016 (25.4)	254 (28.7)	468 (25.2)	198 (25.4)	96 (20.3)		1188 (27.0)	313 (29.1)	326 (26.0)	274 (27.2)	275 (25.8)	
Former regular smoker	1094 (27.4)	209 (23.6)	482 (26.0)	241 (30.9)	162 (34.2)		849 (19.3)	190 (17.6)	228 (18.2)	197 (19.6)	234 (22.0)	
Current smoker	208 (5.2)	46 (5.2)	91 (4.9)	38 (4.9)	33 (7.0)		155 (3.5)	24 (2.2)	51 (4.1)	36 (3.6)	44 (4.1)	
Missing	25 (0.6)	3 (0.3)	12 (0.6)	6 (0.8)	4 (0.8)		36 (0.8)	6 (0.6)	12 (1.0)	9 (0.9)	9 (0.8)	
Alcohol intake: n (%)												
Up to three times a month	818 (20.5)	165 (18.6)	386 (20.8)	161 (20.6)	106 (22.4)	0.129	1460 (33.2)	394 (36.6)	394 (31.4)	340 (33.8)	332 (31.2)	0.006
Up to four times a week	2278 (57.0)	533 (60.2)	1074 (57.8)	420 (53.8)	251 (53.0)		2287 (52.0)	564 (52.4)	665 (53.1)	502 (49.9)	556 (52.2)	
Daily	885 (22.1)	186 (21.0)	390 (21.0)	195 (25.0)	114 (24.1)		628 (14.3)	115 (10.7)	184 (14.7)	158 (15.7)	171 (16.1)	
Missing	16 (0.4)	2 (0.2)	7 (0.4)	4 (0.5)	3 (0.6)		27 (0.6)	4 (0.4)	10 (0.8)	7 (0.7)	6 (0.6)	
Physical activity: n (%)												
Inactive	458 (11.5)	85 (9.6)	193 (10.4)	117 (15.0)	63 (13.3)	<0.001	568 (12.9)	134 (12.4)	137 (10.9)	145 (14.4)	152 (14.3)	<0.001
Moderately active	1896 (47.4)	430 (48.5)	805 (43.3)	414 (53.1)	247 (52.1)		2438 (55.4)	573 (53.2)	660 (52.7)	604 (60.0)	601 (56.4)	
Active	,623 (40.6)	368 (41.5)	852 (45.9)	244 (31.3)	159 (33.5)		1365 (31.0)	365 (33.9)	444 (35.4)	250 (24.8)	306 (28.7)	
Missing	20 (0.5)	3 (0.3)	7 (0.4)	5 (0.6)	5 (1.1)		31 (0.7)	5 (0.5)	12 (1.0)	8 (0.8)	6 (0.6)	
Townsend index: n (%)												
Lowest tertile	1331 (33.3)	303 (34.2)	605 (32.6)	266 (34.1)	157 (33.1)	0.867	1466 (33.3)	371 (34.4)	436 (34.8)	321 (31.9)	338 (31.7)	0.48
Middle tertile	1332 (33.3)	297 (33.5)	616 (33.2)	265 (34.0)	154 (32.5)		1467 (33.3)	377 (35.0)	393 (31.4)	337 (33.5)	360 (33.8)	
Highest tertile	1331 (33.3)	286 (32.3)	634 (34.1)	249 (31.9)	162 (34.2)		1465 (33.3)	328 (30.5)	423 (33.8)	348 (34.6)	366 (34.4)	
Missing	3 (0.1)	0	2 (0.1)	0	1 (0.2)		4 (0.1)	1 (0.1)	1 (0.1)	1 (0.1)	1 (0.1)	
MP status & HRT use: n (%)												
Pre-MP							585 (13.3)	145 (13.5)	234 (18.7)	81 (8.0)	125 (11.7)	<0.001
Post-MP Never user							2169 (49.3)	540 (50.1)	601 (48.0)	489 (48.6)	539 (50.6)	
Post-MP Former user							1354 (30.8)	314 (29.2)	330 (26.3)	381 (37.8)	329 (30.9)	
Post-MP Current user							268 (6.1)	73 (6.8)	79 (6.3)	50 (5.0)	66 (6.2)	
Post-MP Missing							26 (0.6)	5 (0.5)	9 (0.7)	6 (0.6)	6 (0.6)	

ABSI – a body shape index (cut-offs: ≥80 in men; ≥73 in women); **Apple** – large-ABSI-small-HI; **DXA** – dual-emission X-ray absorptiometry; **HI** – hip index (cut-offs: ≥49 in men; ≥64 in women); **HRT** – hormone replacement therapy; **MP** – menopausal; **MRI** – magnetic resonance imaging; **n (%)** – number of participants (percent from the total per column or, for cohort size, percent from column overall); **P** – p-values from one-way ANOVA (continuous variables) or chi-squared test (categorical variables) comparing body-shape phenotypes per sex; **Pear** – small-ABSI-large-HI; **SD** – standard deviation; **Slim** – small-ABSI-small-HI; **Wide** – large-ABSI-large-HI.

Supplementary Table S4 Anthropometric characteristics of study participants according to waist-by-hip circumference phenotype

	Overall	Small Waist Large Hip	Small Waist Small Hip	Large Waist Large Hip	Large Waist Small Hip
**Cancer risk dataset (MEN)**					
Cohort size: n (%)	200,289	20,031 (10.0)	76,398 (38.1)	84,061 (42.0)	19,799 (9.9)
Colon cancer cases: n	1029	77 (7.5)	283 (27.5)	525 (51.0)	144 (14.0)
Age at enrolment (years)	57.2 (8.1)	54.9 (8.5)	56.7 (8.2)	57.7 (7.9)	59.6 (7.4)
Waist circumference (cm)	96.9 (11.0)	91.6 (3.3)	87.1 (5.5)	106.4 (8.3)	99.7 (3.7)
Hip circumference (cm)	103.4 (7.2)	105.0 (2.1)	97.2 (3.6)	109.6 (5.8)	99.9 (2.2)
Waist-to-hip ratio	0.94 (0.06)	0.87 (0.03)	0.90 (0.05)	0.97 (0.05)	1.00 (0.04)
A Body Shape Index (ABSI)	79.8 (4.1)	77.2 (3.3)	78.2 (3.9)	81.2 (3.6)	82.7 (3.6)
Hip Index (HI)	49.1 (1.7)	50.2 (1.3)	48.7 (1.5)	49.5 (1.6)	47.8 (1.4)
Waist-to-Hip Index (WHI)	4.08 (0.22)	3.85 (0.15)	4.03 (0.20)	4.12 (0.19)	4.35 (0.17)
Weight (kg)	86.1 (13.8)	84.6 (6.1)	74.7 (7.3)	97.3 (11.7)	83.6 (6.8)
Height (cm)	175.9 (6.8)	178.5 (6.4)	174.2 (6.5)	177.3 (6.7)	173.5 (6.3)
Body Mass Index (BMI) (kg/m^2)	27.8 (4.0)	26.6 (1.9)	24.6 (2.2)	31.0 (3.6)	27.8 (2.1)
Cohort size by BMI category					
NW: ≥18.5 to <25 kg/m^2: n (%)	49,992 (25.0)	4058 (20.3)	43,390 (56.8)	991 (1.2)	1553 (7.8)
OW: ≥25 to <30 kg/m^2: n (%)	99,591 (49.7)	15,133 (75.5)	32,389 (42.4)	36,643 (43.6)	15,426 (77.9)
OB: ≥30 to <45 kg/m^2: n (%)	50,706 (25.3)	840 (4.2)	619 (0.8)	46,427 (55.2)	2820 (14.2)
Colon cancer cases by BMI					
NW colon cancer cases: n (%)	193 (18.8)	13 (16.9)	160 (56.5)	3 (0.6)	17 (11.8)
OW colon cancer cases: n (%)	511 (49.7)	61 (79.2)	119 (42.0)	221 (42.1)	110 (76.4)
OB colon cancer cases: n (%)	325 (31.6)	3 (3.9)	4 (1.4)	301 (57.3)	17 (11.8)
**Cancer risk dataset (WOMEN)**					
Cohort size: n (%)	230,326	23,835 (10.3)	89,617 (38.9)	93,781 (40.7)	23,093 (10.0)
Colon cancer cases: n (%)	889	78 (8.8)	300 (33.7)	407 (45.8)	104 (11.7)
Age at enrolment (years)	56.8 (8.0)	55.5 (8.1)	55.9 (8.1)	57.6 (7.7)	58.8 (7.5)
Waist circumference (cm)	84.4 (12.0)	78.1 (3.3)	73.8 (5.0)	95.3 (9.2)	87.4 (4.3)
Hip circumference (cm)	103.2 (9.7)	104.9 (3.1)	95.0 (4.1)	111.8 (8.1)	98.2 (2.7)
Waist-to-hip ratio	0.82 (0.07)	0.74 (0.04)	0.78 (0.05)	0.85 (0.06)	0.89 (0.05)
A Body Shape Index (ABSI)	73.8 (5.0)	69.6 (3.3)	71.8 (4.0)	75.7 (4.6)	78.4 (4.1)
Hip Index (HI)	64.2 (2.5)	65.8 (2.0)	63.8 (2.2)	64.7 (2.5)	62.3 (2.3)
Waist-to-Hip Index (WHI)	3.59 (0.27)	3.30 (0.16)	3.55 (0.21)	3.62 (0.25)	3.94 (0.21)
Weight (kg)	71.2 (13.1)	70.2 (5.4)	60.5 (5.8)	82.6 (11.5)	67.7 (5.9)
Height (cm)	162.6 (6.2)	164.6 (6.2)	162.0 (6.1)	163.0 (6.2)	161.2 (6.1)
Body Mass Index (BMI) (kg/m^2)	26.9 (4.8)	26.0 (2.0)	23.0 (2.1)	31.1 (4.2)	26.1 (2.2)
Cohort size by BMI category					
NW: ≥18.5 to <25 kg/m^2: n (%)	92,272 (40.1)	7900 (33.1)	74,293 (82.9)	2786 (3.0)	7293 (31.6)
OW: ≥25 to <30 kg/m^2: n (%)	85,748 (37.2)	15,137 (63.5)	15,213 (17.0)	40,703 (43.4)	14,695 (63.6)
OB: ≥30 to <45 kg/m^2: n (%)	52,306 (22.7)	798 (3.3)	111 (0.1)	50,292 (53.6)	1105 (4.8)
Colon cancer cases by BMI					
NW colon cancer cases: n (%)	307 (34.5)	25 (32.1)	242 (80.7)	8 (2.0)	32 (30.8)
OW colon cancer cases: n (%)	352 (39.6)	48 (61.5)	58 (19.3)	178 (43.7)	68 (65.4)
OB colon cancer cases: n (%)	230 (25.9)	5 (6.4)	0	221 (54.3)	4 (3.8)

n (%) – number of participants (percentage from the total per column or, for cohort size and colon cancer cases, percentage from row overall); **NW** – normal weight BMI (86.8% of NW men and 80.5% of NW women were classified as “slim” body-shape phenotype); **OB** – obese BMI (91.6% of OB men and 96.1% of OB women were classified as “wide” body-shape phenotype); **OW** – overweight BMI.

Waist and hip circumference were dichotomised at the sex-specific medians (≥96 cm for men and ≥83 cm for women for large waist circumference; ≥103 cm for men and ≥102 cm for women for large hip circumference). Continuous variables are summarised with mean (standard deviation). The four body-shape phenotypes per sex were compared with one-way ANOVA for continuous variables and chi-squared test for categorical variables. All comparisons were significant at $p<0.001$.

Supplementary Table S5 Overlap between ABSI-by-HI phenotypes and WHI quartiles

	Cancer risk dataset					DXA dataset					MRI dataset			
	Pear	Slim	Wide	Apple		Pear	Slim	Wide	Apple		Pear	Slim	Wide	Apple
**Men**														
**Tot**	23.7	28.3	29.3	18.7	**Tot**	16.2	44.8	19.7	19.3	**Tot**	22.2	46.5	19.5	11.9
**Q1**	16.2	7.2	1.5	0	**Q1**	11.8	11.8	1.4	0	**Q1**	13.5	10.5	1.0	0
**Q2**	7.2	10.1	7.6	0	**Q2**	4.3	14.9	5.8	0	**Q2**	7.3	14.0	3.7	0
**Q3**	0.2	8.6	12.2	3.9	**Q3**	0.1	13.0	7.8	4.2	**Q3**	1.3	14.2	8.4	1.2
**Q4**	0	2.3	7.9	14.8	**Q4**	0	5.1	4.8	15.1	**Q4**	0	7.7	6.6	10.7
**Women**														
**Tot**	24.0	21.3	30.8	23.8	**Tot**	19.2	26.6	27.7	26.4	**Tot**	24.5	28.5	22.9	24.2
**Q1**	17.5	5.7	1.8	0	**Q1**	15.2	8.0	1.8	0	**Q1**	17.5	6.6	1.0	0
**Q2**	6.4	9.3	8.9	0.3	**Q2**	4.0	11.7	8.8	0.6	**Q2**	6.8	11.3	6.8	0.2
**Q3**	0.1	5.5	12.2	7.2	**Q3**	0	6.2	10.3	8.6	**Q3**	0.2	9.0	9.6	6.2
**Q4**	0	0.8	7.9	16.3	**Q4**	0	0.8	6.9	17.3	**Q4**	0	1.6	5.6	17.8

ABSI – A Body Shape Index (cut-offs: ≥80 in men; ≥73 in women); **Apple** – large-ABSI-small-HI; **DXA** – dual-emission X-ray absorptiometry; **HI** – hip index (cut-offs: ≥49 in men; ≥64 in women); **MRI** – magnetic resonance imaging; **Pear** – small-ABSI-large-HI; **Q1-Q4** – WHI quartiles (quartile boundaries were: 3.933, 4.075, 4.221 for men and 3.410, 3.578, 3.760 for women in the cancer risk dataset; 3.946, 4.088, 4.235 for men and 3.436, 3.608, 3.797 for women in the DXA dataset; 3.905, 4.027, 4.156 for men and 3.404, 3.569, 3.737 for women in the MRI dataset); **Slim** – small-ABSI-small-HI; **Tot** – overall dataset; **WHI** – waist-to-hip index; **Wide** – large-ABSI-large-HI. **Cells** – show the percentage from the total per column (for rows Q1-Q4) or from the total per dataset and sex (for row Tot). The total number of participants per body-shape phenotype are shown in Table 1.

Supplementary Table S6 Body-composition of body-shape phenotypes

	Body shape	Men	Women	Men	Women
		Fat-free Mass		Fat Mass	
**BIA Total Mass**	Pear	reference	reference	reference	reference
	Slim	0.13 (0.09 to 0.17)	0.29 (0.25 to 0.34)	-0.08 (-0.12 to -0.03)	-0.24 (-0.28 to -0.20)
	Wide	-0.50 (-0.55 to -0.45)	-0.24 (-0.29 to -0.20)	0.48 (0.43 to 0.53)	0.22 (0.17 to 0.26)
	Apple	-0.32 (-0.37 to -0.27)	0.05 (0.00 to 0.09)	0.34 (0.29 to 0.39)	0.00 (-0.04 to 0.05)
**BIA Arms Mass**	Pear	reference	reference	reference	reference
	Slim	0.28 (0.24 to 0.32)	0.36 (0.32 to 0.41)	-0.24 (-0.28 to -0.20)	-0.24 (-0.29 to -0.20)
	Wide	-0.41 (-0.46 to -0.36)	-0.15 (-0.19 to -0.10)	0.41 (0.35 to 0.46)	0.11 (0.07 to 0.16)
	Apple	-0.16 (-0.20 to -0.11)	0.22 (0.18 to 0.27)	0.17 (0.12 to 0.22)	-0.10 (-0.15 to -0.06)
**BIA Legs Mass**	Pear	reference	reference	reference	reference
	Slim	-0.06 (-0.10 to -0.02)	0.13 (0.08 to 0.17)	0.07 (0.03 to 0.12)	-0.10 (-0.14 to -0.06)
	Wide	-0.39 (-0.44 to -0.34)	-0.29 (-0.34 to -0.25)	0.44 (0.38 to 0.49)	0.27 (0.22 to 0.31)
	Apple	-0.34 (-0.39 to -0.30)	-0.17 (-0.22 to -0.13)	0.39 (0.34 to 0.44)	0.18 (0.13 to 0.22)
**BIA Trunk Mass**	Pear	reference	reference	reference	reference
	Slim	0.17 (0.12 to 0.21)	0.32 (0.28 to 0.37)	-0.11 (-0.15 to -0.06)	-0.25 (-0.30 to -0.21)
	Wide	-0.48 (-0.53 to -0.43)	-0.19 (-0.24 to -0.15)	0.44 (0.39 to 0.49)	0.19 (0.15 to 0.24)
	Apple	-0.28 (-0.33 to -0.23)	0.12 (0.08 to 0.17)	0.29 (0.24 to 0.34)	-0.03 (-0.08 to 0.01)
		Lean Mass		Fat Mass	
**DXA Total Mass**	Pear	reference	reference	reference	reference
	Slim	0.09 (0.05 to 0.14)	0.30 (0.26 to 0.34)	0.00 (-0.04 to 0.05)	-0.28 (-0.33 to -0.24)
	Wide	-0.54 (-0.59 to -0.49)	-0.16 (-0.20 to -0.11)	0.54 (0.49 to 0.59)	0.14 (0.10 to 0.19)
	Apple	-0.45 (-0.50 to -0.40)	0.08 (0.04 to 0.13)	0.53 (0.48 to 0.58)	-0.07 (-0.11 to -0.02)
**DXA Arms Mass**	Pear	reference	reference	reference	reference
	Slim	0.21 (0.17 to 0.25)	0.39 (0.34 to 0.43)	-0.06 (-0.10 to -0.01)	0.03 (-0.01 to 0.08)
	Wide	-0.39 (-0.44 to -0.34)	-0.01 (-0.06 to 0.03)	0.29 (0.23 to 0.34)	0.13 (0.08 to 0.17)
	Apple	-0.24 (-0.29 to -0.19)	0.34 (0.30 to 0.39)	0.19 (0.13 to 0.24)	0.20 (0.16 to 0.25)
**DXA Legs Mass**	Pear	reference	reference	reference	reference
	Slim	-0.02 (-0.06 to 0.02)	0.18 (0.14 to 0.22)	-0.35 (-0.40 to -0.31)	-0.55 (-0.59 to -0.50)
	Wide	-0.52 (-0.57 to -0.47)	-0.24 (-0.28 to -0.20)	0.13 (0.08 to 0.18)	-0.63 (-0.68 to -0.59)
	Apple	-0.47 (-0.52 to -0.42)	-0.10 (-0.15 to -0.06)	-0.23 (-0.28 to -0.18)	-1.13 (-1.18 to -1.09)
**DXA Gynoid Mass**	Pear	reference	reference	reference	reference
	Slim	-0.11 (-0.15 to -0.07)	0.11 (0.07 to 0.15)	-0.38 (-0.43 to -0.34)	-0.69 (-0.73 to -0.65)
	Wide	-0.53 (-0.58 to -0.48)	-0.27 (-0.32 to -0.23)	0.24 (0.19 to 0.30)	-0.52 (-0.57 to -0.48)
	Apple	-0.64 (-0.69 to -0.59)	-0.22 (-0.27 to -0.18)	-0.15 (-0.21 to -0.10)	-1.18 (-1.22 to -1.14)
**DXA Trunk Mass**	Pear	reference	reference	reference	reference
	Slim	0.08 (0.04 to 0.12)	0.26 (0.22 to 0.31)	0.15 (0.11 to 0.20)	0.01 (-0.04 to 0.05)
	Wide	-0.43 (-0.48 to -0.38)	-0.08 (-0.13 to -0.04)	0.59 (0.54 to 0.64)	0.54 (0.50 to 0.59)
	Apple	-0.36 (-0.41 to -0.31)	0.12 (0.07 to 0.16)	0.74 (0.69 to 0.79)	0.61 (0.56 to 0.65)
**DXA Android Mass**	Pear	reference	reference	reference	reference
	Slim	0.04 (-0.01 to 0.08)	0.15 (0.11 to 0.20)	0.17 (0.12 to 0.21)	0.02 (-0.02 to 0.06)
	Wide	-0.24 (-0.29 to -0.19)	0.04 (-0.01 to 0.08)	0.61 (0.56 to 0.66)	0.56 (0.52 to 0.60)
	Apple	-0.16 (-0.22 to -0.11)	0.18 (0.14 to 0.23)	0.78 (0.73 to 0.83)	0.65 (0.61 to 0.69)
		ASAT		VAT	
**DXA Abdominal Mass**	Pear	reference	reference	reference	reference
	Slim	-0.19 (-0.30 to -0.09)	-0.37 (-0.48 to -0.25)	0.26 (0.16 to 0.36)	0.23 (0.12 to 0.34)
	Wide	0.37 (0.24 to 0.50)	0.10 (-0.02 to 0.22)	0.45 (0.33 to 0.58)	0.58 (0.47 to 0.69)
	Apple	0.05 (-0.11 to 0.20)	-0.06 (-0.18 to 0.06)	0.65 (0.50 to 0.80)	1.00 (0.89 to 1.11)
**MRI Abdominal Volume**	Pear	reference	reference	reference	reference
	Slim	-0.18 (-0.26 to -0.11)	-0.31 (-0.39 to -0.23)	0.29 (0.21 to 0.36)	0.14 (0.07 to 0.22)
	Wide	0.55 (0.46 to 0.64)	0.30 (0.21 to 0.38)	0.44 (0.35 to 0.53)	0.57 (0.49 to 0.65)
	Apple	0.32 (0.21 to 0.43)	0.11 (0.02 to 0.19)	0.75 (0.64 to 0.86)	0.99 (0.92 to 1.07)

	Body shape	Men	Women	Men	Women
		Hand Grip Strength		MRI ASAT + MRI VAT	
**Other**	Pear	reference	reference	reference	reference
	Slim	0.10 (0.06 to 0.15)	0.07 (0.03 to 0.12)	0.11 (0.03 to 0.18)	-0.15 (-0.23 to -0.08)
	Wide	-0.17 (-0.22 to -0.12)	-0.13 (-0.18 to -0.09)	0.61 (0.52 to 0.70)	0.48 (0.40 to 0.56)
	Apple	-0.20 (-0.26 to -0.15)	-0.07 (-0.12 to -0.03)	0.71 (0.60 to 0.81)	0.55 (0.47 to 0.63)

ABSI – a body shape index (cut-offs: ≥80 in men; ≥73 in women); **Apple** – large-ABSI-small-HI; **ASAT** – abdominal subcutaneous adipose tissue; **BIA** – bioelectrical impedance analysis; **BMI** – body mass index; **CI** – confidence interval; **DXA** – dual-emission X-ray absorptiometry; **HI** – hip index (cut-offs: ≥49 in men; ≥64 in women); **MRI** – magnetic resonance imaging; **Pear** – small-ABSI-large-HI; **SD** – standard deviation; **Slim** – small-ABSI-small-HI; **VAT** – visceral adipose tissue; **Wide** – large-ABSI-large-HI.

SD difference (95% CI) – derived from linear regression models with adjustment for age, self-reported weight change within the year preceding the visit, smoking status, alcohol consumption, physical activity, Townsend deprivation index, region (except for VAT, ASAT and MRI) and, in women, menopausal status and use of hormonal replacement therapy (see definition of covariates in Supplementary Methods). Body-composition measurements were converted to allometric indices with scaling for height and weight (see scaling coefficients in Supplementary Table S1) and then to sex-specific z-scores (value minus mean, divided by the standard deviation). Note that DXA lean mass does not include bone mass, which is included in BIA fat-free mass. See Supplementary Methods for the calculation of DXA lean and fat mass.

$p<5*10^{-16}$ for all body-composition indices and hand grip strength – p-value obtained from a likelihood ratio test comparing models with and without an ABSI-by-HI variable, i.e. evaluating the significance of the association between each body composition index and body shape overall, accounting for all covariates.

Supplementary Table S7 Body-composition of body-shape phenotypes by BMI category

	Body shape	Men	Women	Men	Women
		DXA Total Lean Mass		DXA Total Fat Mass	
**NW**	Pear	reference	reference	reference	reference
	Slim	0.10 (0.04 to 0.15)	0.20 (0.15 to 0.25)	0.03 (-0.01 to 0.08)	-0.07 (-0.10 to -0.04)
	Wide	-0.31 (-0.37 to -0.25)	-0.15 (-0.19 to -0.10)	0.23 (0.18 to 0.28)	0.05 (0.02 to 0.08)
	Apple	-0.27 (-0.34 to -0.20)	0.05 (0.00 to 0.09)	0.26 (0.21 to 0.31)	0.06 (0.02 to 0.09)
**OW**	Pear	0.91 (0.84 to 0.97)	0.72 (0.66 to 0.77)	0.92 (0.87 to 0.97)	0.96 (0.92 to 1.00)
	Slim	0.93 (0.88 to 0.98)	0.91 (0.86 to 0.96)	0.95 (0.90 to 0.99)	0.88 (0.84 to 0.91)
	Wide	0.51 (0.45 to 0.57)	0.60 (0.55 to 0.65)	1.15 (1.10 to 1.20)	1.04 (1.00 to 1.07)
	Apple	0.58 (0.52 to 0.64)	0.83 (0.78 to 0.88)	1.17 (1.12 to 1.22)	0.99 (0.96 to 1.02)
**OB**	Pear	1.94 (1.86 to 2.02)	1.81 (1.74 to 1.88)	2.46 (2.40 to 2.52)	2.44 (2.39 to 2.48)
	Slim	2.04 (1.98 to 2.10)	2.01 (1.95 to 2.08)	2.12 (2.07 to 2.17)	2.13 (2.09 to 2.18)
	Wide	1.65 (1.57 to 1.72)	1.82 (1.76 to 1.88)	2.68 (2.62 to 2.73)	2.40 (2.37 to 2.44)
	Apple	1.72 (1.65 to 1.79)	1.98 (1.91 to 2.04)	2.43 (2.37 to 2.48)	2.18 (2.14 to 2.22)
$p_{interaction}$		0.219	0.0012	<0.0001	<0.0001
		DXA Arms Lean Mass		DXA Arms Fat Mass	
**NW**	Pear	reference	reference	reference	reference
	Slim	0.19 (0.13 to 0.25)	0.24 (0.19 to 0.29)	0.01 (-0.04 to 0.07)	-0.02 (-0.05 to 0.02)
	Wide	-0.21 (-0.28 to -0.15)	-0.09 (-0.14 to -0.04)	0.17 (0.11 to 0.23)	0.04 (0.00 to 0.08)
	Apple	-0.13 (-0.20 to -0.06)	0.20 (0.14 to 0.25)	0.16 (0.10 to 0.22)	0.09 (0.05 to 0.13)
**OW**	Pear	0.85 (0.79 to 0.92)	0.57 (0.51 to 0.63)	0.84 (0.78 to 0.89)	0.80 (0.76 to 0.85)
	Slim	0.96 (0.90 to 1.02)	0.89 (0.84 to 0.95)	0.83 (0.78 to 0.87)	0.86 (0.82 to 0.90)
	Wide	0.51 (0.44 to 0.57)	0.60 (0.54 to 0.65)	0.98 (0.92 to 1.03)	0.90 (0.85 to 0.94)
	Apple	0.63 (0.57 to 0.70)	0.93 (0.88 to 0.98)	0.94 (0.88 to 0.99)	0.98 (0.94 to 1.02)
**OB**	Pear	1.70 (1.61 to 1.78)	1.55 (1.47 to 1.62)	2.23 (2.16 to 2.31)	2.03 (1.97 to 2.09)
	Slim	1.91 (1.85 to 1.98)	1.91 (1.84 to 1.98)	1.91 (1.85 to 1.97)	2.03 (1.97 to 2.09)
	Wide	1.46 (1.38 to 1.54)	1.70 (1.64 to 1.76)	2.36 (2.30 to 2.43)	2.14 (2.09 to 2.19)
	Apple	1.57 (1.49 to 1.65)	1.93 (1.86 to 2.00)	2.15 (2.08 to 2.22)	2.14 (2.09 to 2.19)
$p_{interaction}$		0.097	<0.0001	<0.0001	0.030
		DXA Legs Lean Mass		DXA Legs Fat Mass	
**NW**	Pear	reference	reference	reference	reference
	Slim	0.03 (-0.02 to 0.09)	0.12 (0.08 to 0.16)	-0.07 (-0.13 to -0.02)	-0.21 (-0.24 to -0.17)
	Wide	-0.27 (-0.34 to -0.21)	-0.15 (-0.19 to -0.10)	0.09 (0.03 to 0.15)	-0.22 (-0.26 to -0.18)
	Apple	-0.25 (-0.31 to -0.18)	-0.02 (-0.06 to 0.03)	0.02 (-0.05 to 0.08)	-0.36 (-0.40 to -0.32)
**OW**	Pear	0.96 (0.90 to 1.02)	0.85 (0.79 to 0.90)	0.79 (0.73 to 0.85)	0.97 (0.92 to 1.02)
	Slim	0.92 (0.87 to 0.97)	0.93 (0.89 to 0.98)	0.63 (0.57 to 0.68)	0.64 (0.60 to 0.68)
	Wide	0.61 (0.55 to 0.67)	0.67 (0.62 to 0.72)	0.88 (0.82 to 0.93)	0.60 (0.55 to 0.64)
	Apple	0.63 (0.57 to 0.69)	0.82 (0.77 to 0.86)	0.69 (0.64 to 0.75)	0.34 (0.30 to 0.38)
**OB**	Pear	2.04 (1.97 to 2.12)	2.03 (1.96 to 2.09)	2.36 (2.29 to 2.44)	2.40 (2.34 to 2.46)
	Slim	2.00 (1.94 to 2.06)	2.10 (2.04 to 2.17)	1.63 (1.57 to 1.68)	1.88 (1.82 to 1.93)
	Wide	1.72 (1.65 to 1.79)	1.91 (1.85 to 1.96)	2.35 (2.28 to 2.42)	1.78 (1.73 to 1.83)
	Apple	1.76 (1.69 to 1.83)	1.92 (1.86 to 1.98)	1.78 (1.71 to 1.85)	1.29 (1.24 to 1.34)
$p_{interaction}$		0.363	0.028	<0.0001	<0.0001
		DXA Gynoid Lean Mass		DXA Gynoid Fat Mass	
**NW**	Pear	reference	reference	reference	reference
	Slim	-0.03 (-0.09 to 0.03)	0.08 (0.03 to 0.13)	-0.08 (-0.13 to -0.03)	-0.23 (-0.26 to -0.19)
	Wide	-0.31 (-0.38 to -0.25)	-0.23 (-0.28 to -0.18)	0.14 (0.09 to 0.20)	-0.16 (-0.20 to -0.12)
	Apple	-0.40 (-0.46 to -0.33)	-0.17 (-0.22 to -0.12)	0.06 (0.00 to 0.12)	-0.34 (-0.37 to -0.30)
**OW**	Pear	0.87 (0.80 to 0.93)	0.71 (0.65 to 0.77)	0.87 (0.81 to 0.92)	1.00 (0.96 to 1.05)
	Slim	0.75 (0.70 to 0.81)	0.78 (0.73 to 0.83)	0.71 (0.66 to 0.75)	0.64 (0.60 to 0.68)
	Wide	0.46 (0.39 to 0.52)	0.51 (0.46 to 0.56)	0.98 (0.93 to 1.04)	0.75 (0.71 to 0.79)
	Apple	0.39 (0.33 to 0.45)	0.61 (0.56 to 0.66)	0.82 (0.77 to 0.87)	0.43 (0.40 to 0.47)
**OB**	Pear	1.88 (1.80 to 1.97)	1.75 (1.68 to 1.82)	2.48 (2.42 to 2.55)	2.45 (2.39 to 2.50)
	Slim	1.78 (1.72 to 1.84)	1.80 (1.73 to 1.87)	1.81 (1.76 to 1.86)	1.83 (1.78 to 1.88)
	Wide	1.49 (1.42 to 1.57)	1.67 (1.61 to 1.73)	2.55 (2.49 to 2.61)	2.01 (1.96 to 2.05)
	Apple	1.38 (1.30 to 1.45)	1.68 (1.62 to 1.74)	1.95 (1.89 to 2.01)	1.46 (1.42 to 1.51)
$p_{interaction}$		0.244	0.0005	<0.0001	<0.0001

	Body shape	Men	Women	Men	Women
		DXA Trunk Lean Mass		DXA Trunk Fat Mass	
NW	Pear	reference	reference	reference	reference
	Slim	0.08 (0.02 to 0.15)	0.22 (0.17 to 0.27)	0.07 (0.03 to 0.11)	0.00 (-0.03 to 0.03)
	Wide	-0.33 (-0.40 to -0.25)	-0.13 (-0.19 to -0.08)	0.27 (0.22 to 0.32)	0.19 (0.15 to 0.22)
	Apple	-0.29 (-0.37 to -0.22)	0.04 (-0.02 to 0.10)	0.34 (0.29 to 0.39)	0.26 (0.22 to 0.29)
OW	Pear	0.65 (0.58 to 0.72)	0.45 (0.38 to 0.52)	0.91 (0.86 to 0.96)	0.87 (0.83 to 0.91)
	Slim	0.68 (0.61 to 0.74)	0.66 (0.60 to 0.72)	1.00 (0.96 to 1.05)	0.90 (0.86 to 0.93)
	Wide	0.26 (0.19 to 0.33)	0.38 (0.32 to 0.44)	1.19 (1.14 to 1.24)	1.17 (1.13 to 1.20)
	Apple	0.34 (0.27 to 0.41)	0.60 (0.54 to 0.66)	1.29 (1.24 to 1.33)	1.21 (1.18 to 1.24)
OB	Pear	1.49 (1.40 to 1.58)	1.28 (1.20 to 1.36)	2.37 (2.31 to 2.43)	2.25 (2.20 to 2.30)
	Slim	1.64 (1.57 to 1.71)	1.51 (1.43 to 1.59)	2.18 (2.13 to 2.23)	2.04 (1.99 to 2.09)
	Wide	1.25 (1.17 to 1.33)	1.37 (1.30 to 1.44)	2.66 (2.60 to 2.72)	2.50 (2.46 to 2.54)
	Apple	1.31 (1.23 to 1.40)	1.60 (1.53 to 1.67)	2.53 (2.48 to 2.59)	2.39 (2.34 to 2.43)
$p_{interaction}$		0.173	<0.0001	<0.0001	<0.0001
		DXA Android Lean Mass		DXA Android Fat Mass	
NW	Pear	reference	reference	reference	reference
	Slim	0.04 (-0.02 to 0.11)	0.12 (0.07 to 0.17)	0.07 (0.03 to 0.12)	0.01 (-0.02 to 0.04)
	Wide	-0.15 (-0.23 to -0.08)	-0.02 (-0.07 to 0.04)	0.29 (0.24 to 0.34)	0.21 (0.17 to 0.24)
	Apple	-0.11 (-0.19 to -0.04)	0.10 (0.04 to 0.15)	0.37 (0.32 to 0.42)	0.29 (0.25 to 0.32)
OW	Pear	0.58 (0.51 to 0.65)	0.49 (0.43 to 0.56)	0.87 (0.82 to 0.92)	0.84 (0.80 to 0.88)
	Slim	0.60 (0.54 to 0.66)	0.59 (0.53 to 0.65)	0.97 (0.93 to 1.01)	0.86 (0.82 to 0.89)
	Wide	0.35 (0.28 to 0.42)	0.50 (0.45 to 0.56)	1.17 (1.13 to 1.22)	1.15 (1.11 to 1.19)
	Apple	0.45 (0.38 to 0.52)	0.67 (0.62 to 0.73)	1.29 (1.24 to 1.34)	1.20 (1.17 to 1.24)
OB	Pear	1.58 (1.48 to 1.67)	1.39 (1.32 to 1.47)	2.33 (2.26 to 2.39)	2.22 (2.17 to 2.27)
	Slim	1.65 (1.58 to 1.72)	1.54 (1.46 to 1.62)	2.14 (2.09 to 2.19)	1.98 (1.93 to 2.03)
	Wide	1.49 (1.40 to 1.57)	1.60 (1.53 to 1.66)	2.65 (2.59 to 2.70)	2.51 (2.46 to 2.55)
	Apple	1.52 (1.44 to 1.61)	1.81 (1.74 to 1.88)	2.54 (2.48 to 2.60)	2.40 (2.36 to 2.45)
$p_{interaction}$		0.242	<0.0001	<0.0001	<0.0001
		DXA ASAT Mass		DXA VAT Mass	
NW	Pear	reference	reference	reference	reference
	Slim	-0.05 (-0.21 to 0.11)	-0.11 (-0.20 to -0.01)	0.09 (-0.04 to 0.22)	0.09 (-0.01 to 0.19)
	Wide	0.26 (0.08 to 0.44)	0.06 (-0.05 to 0.16)	0.18 (0.03 to 0.33)	0.28 (0.16 to 0.39)
	Apple	0.14 (-0.10 to 0.38)	0.15 (0.04 to 0.26)	0.23 (0.03 to 0.42)	0.48 (0.36 to 0.60)
OW	Pear	0.74 (0.58 to 0.91)	0.98 (0.86 to 1.09)	0.81 (0.68 to 0.95)	0.58 (0.46 to 0.71)
	Slim	0.60 (0.46 to 0.74)	0.75 (0.65 to 0.86)	0.96 (0.84 to 1.08)	0.75 (0.63 to 0.86)
	Wide	0.92 (0.74 to 1.09)	1.05 (0.93 to 1.16)	1.01 (0.87 to 1.15)	1.01 (0.89 to 1.14)
	Apple	0.64 (0.45 to 0.83)	0.88 (0.78 to 0.99)	1.20 (1.05 to 1.36)	1.39 (1.27 to 1.50)
OB	Pear	1.83 (1.62 to 2.03)	2.28 (2.14 to 2.42)	2.04 (1.87 to 2.20)	1.48 (1.32 to 1.63)
	Slim	1.21 (1.03 to 1.38)	1.83 (1.69 to 1.97)	2.20 (2.06 to 2.34)	1.84 (1.69 to 1.99)
	Wide	2.03 (1.80 to 2.26)	2.28 (2.16 to 2.41)	2.17 (1.99 to 2.36)	2.24 (2.11 to 2.38)
	Apple	1.64 (1.37 to 1.91)	1.76 (1.62 to 1.89)	2.25 (2.03 to 2.47)	2.56 (2.42 to 2.71)
$p_{interaction}$		<0.0001	<0.0001	0.673	<0.0001
		MRI ASAT Volume		MRI VAT Volume	
NW	Pear	reference	reference	reference	reference
	Slim	-0.01 (-0.10 to 0.07)	-0.06 (-0.12 to -0.01)	0.10 (0.00 to 0.19)	0.06 (-0.01 to 0.13)
	Wide	0.19 (0.09 to 0.29)	0.16 (0.10 to 0.23)	0.23 (0.12 to 0.33)	0.31 (0.23 to 0.39)
	Apple	0.19 (0.07 to 0.31)	0.18 (0.11 to 0.25)	0.36 (0.23 to 0.49)	0.50 (0.43 to 0.58)
OW	Pear	0.84 (0.74 to 0.93)	0.94 (0.87 to 1.01)	0.82 (0.72 to 0.92)	0.59 (0.51 to 0.68)
	Slim	0.75 (0.67 to 0.83)	0.86 (0.79 to 0.92)	1.05 (0.97 to 1.14)	0.79 (0.71 to 0.87)
	Wide	1.09 (0.99 to 1.19)	1.13 (1.06 to 1.20)	1.08 (0.97 to 1.18)	1.10 (1.01 to 1.18)
	Apple	0.96 (0.85 to 1.06)	1.06 (1.00 to 1.13)	1.33 (1.21 to 1.44)	1.50 (1.42 to 1.58)
OB	Pear	2.31 (2.19 to 2.42)	2.36 (2.27 to 2.45)	1.92 (1.80 to 2.04)	1.70 (1.59 to 1.80)
	Slim	1.93 (1.83 to 2.03)	2.00 (1.91 to 2.08)	2.14 (2.04 to 2.25)	1.84 (1.73 to 1.94)
	Wide	2.67 (2.54 to 2.79)	2.51 (2.43 to 2.59)	2.19 (2.05 to 2.32)	2.22 (2.12 to 2.31)
	Apple	2.30 (2.15 to 2.45)	1.99 (1.90 to 2.08)	2.38 (2.22 to 2.54)	2.44 (2.33 to 2.55)
$p_{interaction}$		<0.0001	<0.0001	0.325	<0.0001

	Body shape	Men	Women	Men	Women
		**Hand Grip Strength**		**MRI ASAT + MRI VAT Volume**	
**NW**	Pear	reference	reference	reference	reference
	Slim	0.07 (0.00 to 0.15)	0.07 (0.01 to 0.13)	0.04 (-0.04 to 0.12)	-0.03 (-0.08 to 0.03)
	Wide	-0.11 (-0.19 to -0.02)	-0.11 (-0.17 to -0.05)	0.23 (0.14 to 0.32)	0.22 (0.16 to 0.28)
	Apple	-0.18 (-0.27 to -0.09)	-0.02 (-0.09 to 0.04)	0.29 (0.18 to 0.41)	0.29 (0.23 to 0.36)
**OW**	Pear	0.16 (0.08 to 0.25)	0.11 (0.03 to 0.19)	0.90 (0.82 to 0.99)	0.88 (0.81 to 0.95)
	Slim	0.26 (0.19 to 0.33)	0.18 (0.11 to 0.25)	0.98 (0.90 to 1.05)	0.88 (0.82 to 0.95)
	Wide	-0.03 (-0.11 to 0.05)	-0.01 (-0.08 to 0.05)	1.18 (1.09 to 1.26)	1.18 (1.11 to 1.25)
	Apple	-0.02 (-0.10 to 0.06)	0.01 (-0.06 to 0.07)	1.23 (1.14 to 1.33)	1.26 (1.20 to 1.33)
**OB**	Pear	0.18 (0.07 to 0.29)	0.21 (0.12 to 0.30)	2.30 (2.20 to 2.41)	2.27 (2.19 to 2.36)
	Slim	0.35 (0.26 to 0.43)	0.31 (0.22 to 0.40)	2.21 (2.12 to 2.30)	2.05 (1.97 to 2.14)
	Wide	-0.04 (-0.14 to 0.05)	-0.03 (-0.11 to 0.05)	2.65 (2.53 to 2.76)	2.55 (2.47 to 2.63)
	Apple	-0.12 (-0.21 to -0.02)	0.03 (-0.05 to 0.11)	2.54 (2.41 to 2.68)	2.25 (2.16 to 2.33)
**$p_{interaction}$**		0.0008	0.014	0.0037	<0.0001

ABSI – a body shape index (cut-offs: ≥80 in men; ≥73 in women); **Apple** – large-ABSI-small-HI; **ASAT** – abdominal subcutaneous adipose tissue; **BMI** – body mass index; **CI** – confidence interval; **DXA** – dual-emission X-ray absorptiometry measurements; **HI** – hip index (cut-offs: ≥49 in men; ≥64 in women); **MRI** – magnetic resonance imaging measurements; **NW** – normal weight BMI≥18.5 to <25.0 kg/m^2; **OB** – obese BMI≥30.0 to <45 kg/m^2; **OW** – overweight BMI≥25.0 to <30.0 kg/m^2; **Pear** – small-ABSI-large-HI; **SD** – standard deviation; **Slim** – small-ABSI-small-HI; **VAT** – visceral adipose tissue; **Wide** – large-ABSI-large-HI.

SD difference (95% CI) – derived from linear regression models with adjustment for age, self-reported weight change within the year preceding the visit, smoking status, alcohol consumption, physical activity, Townsend deprivation index, region (except for VAT, ASAT and MRI) and, in women, menopausal status and use of hormonal replacement therapy (see definition of covariates in Supplementary Methods). Body-composition measurements were converted to allometric indices with scaling for height (see scaling coefficients in Supplementary Table S1) and then to sex-specific z-scores (value minus mean, divided by the standard deviation).

$p_{interaction}$ – derived from a likelihood ratio test comparing a model with separate ABSI-by-HI and BMI categorical variables with a model with BMI-by-ABSI-by-HI cross-classification.

Supplementary Table S8 Body-shape phenotypes in relation to colon cancer risk

	**MEN**		**WOMEN**	
**BMI category**	**BMI** $_{OW\ vs\ NW}$ [a]	**BMI** $_{OB\ vs\ NW}$ [a]	**BMI** $_{OW\ vs\ NW}$ [a]	**BMI** $_{OB\ vs\ NW}$ [a]
Overall [a]	1.18 (1.00 to 1.40)	1.43 (1.19 to 1.73)**	1.09 (0.93 to 1.27)	1.18 (0.98 to 1.42)
**ABSI and HI dichotomised**	**ABSI** $_{large\ vs\ small}$ [a]	**HI** $_{large\ vs\ small}$ [a]	**ABSI** $_{large\ vs\ small}$ [a]	**HI** $_{large\ vs\ small}$ [a]
Overall [a]	1.25 (1.10 to 1.42)**	0.85 (0.75 to 0.96)*	1.11 (0.96 to 1.28)	0.90 (0.78 to 1.02)
**Body-shape contrasts**	**Apple vs Pear** [b,c]	**Wide vs Slim** [b,c]	**Apple vs Pear** [b,c]	**Wide vs Slim** [b,c]
Overall [b]	1.48 (1.21 to 1.80)**	1.06 (0.89 to 1.25)	1.24 (1.02 to 1.51)*	0.99 (0.82 to 1.21)
NW: BMI ≥18.5 to <25.0 [c]	1.77 (1.16 to 2.69)*	1.05 (0.70 to 1.56)	1.25 (0.91 to 1.70)	1.03 (0.74 to 1.44)
OW: BMI ≥25.0 to <30.0 [c]	1.38 (1.04 to 1.82)*	1.24 (0.98 to 1.58)	1.49 (1.08 to 2.05)*	0.94 (0.69 to 1.28)
OB: BMI ≥30.0 to <45.0 [c]	1.50 (1.05 to 2.15)*	0.85 (0.64 to 1.14)	0.99 (0.65 to 1.52)	0.96 (0.66 to 1.39)

ABSI – A Body Shape Index; **Apple** – large-ABSI-small-HI; **BMI** – body mass index (kg/m^2); **HI** – hip index (cut-offs: ≥49 in men; ≥64 in women); **NW** – normal weight (BMI ≥18.5 to <25.0); **OB** – obese (BMI ≥25.0 to <30.0); **OW** – overweight (BMI ≥30.0 to <45.0); **Pear** – small-ABSI-large-HI; **Slim** – small-ABSI-small-HI; **Wide** – large-ABSI-large-HI.

[a] – hazard ratios (95% confidence intervals) were obtained from a Cox proportional hazards model including ABSI (dichotomised at ≥80 for men and ≥73 for women), HI (dichotomised at ≥49 for men and ≥64 for women) and categorical BMI (NW, OW, OB), with stratification for age at enrolment and region of the assessment centre and adjustment for height, smoking status, alcohol consumption, physical activity, Townsend deprivation index, diet (consumption of fresh fruit and vegetables, processed and red meat and fibre), family history of cancer, and, in women, menopausal status, use of oral contraceptives and hormone replacement therapy and age at last live birth;

[b] – as for ([a]) but including an ABSI-by-HI cross-classification variable and with BMI on a continuous scale;

[c] – as for ([a]) but including a BMI-by-ABSI-by-HI cross-classification variable and omitting the adjustment for BMI (pairwise comparisons between body-shape phenotypes are within the corresponding BMI category).

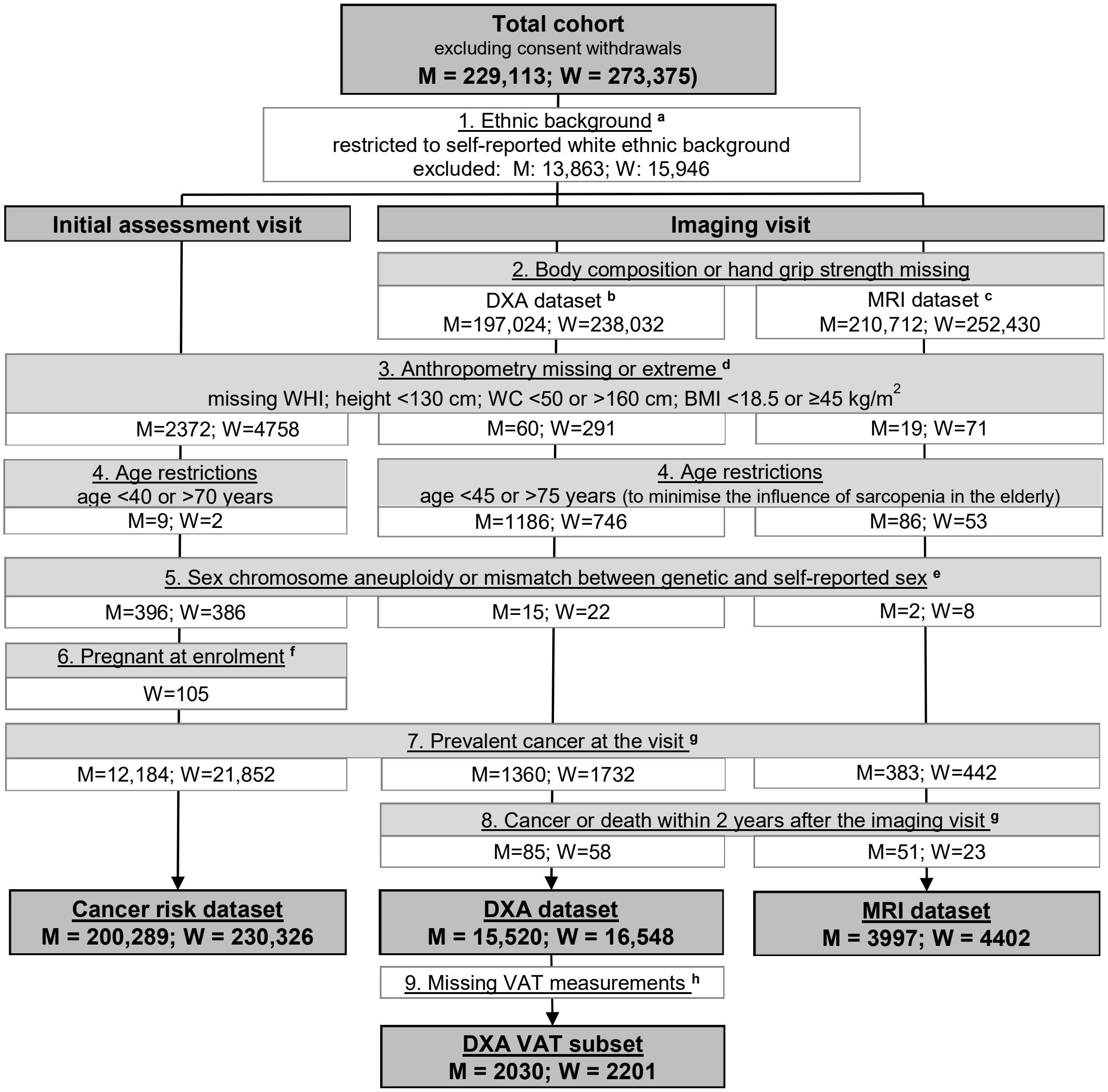

Supplementary Figure S1 Flow diagram of UK Biobank participants included in the study

BMI – body mass index; **DXA** – dual-emission X-ray absorptiometry scan; **M** – number of men; **MRI** – magnetic resonance imaging scan; **VAT** – visceral adipose tissue; **W** – number of women; **WC** – waist circumference; **WHI** – waist-to-hip index, calculated using all anthropometric measurements (waist and hip circumference, weight, and height) and hence missing if any of them were missing. Supplementary Methods include details of the definition of variables. The exclusion criteria were applied sequentially in the displayed order, such that each excluded individual was counted only once. Specific fields used to define the exclusions are listed below:

[a] – Field [21000-0.0] "*Ethnic background*"; retained in the study were codes: 1 "*White*", 1001 "*British*", 1002 "*Irish*", 1003 "*Any other white background*".

[b] – exclusions in the DXA dataset were determined by missing values in any of the DXA OR bioelectrical impedance measurements OR hand grip strength at the imaging visit: Field [23283-2.0] "*Total mass*" (NA or 0); Field [23100-2.0] "*Whole body fat mass*"; Field [23101-2.0] "*Whole body fat-free mass*"; the sum of Field [23109-2.0] "*Impedance of arm (right)*" and Field [23110-2.0] "*Impedance of arm (left)*"; Field [23129-2.0] "*Trunk fat-free mass*"; Field [46-2.0] "*Hand grip strength (left)*"; Field [47-2.0] "*Hand grip strength (right)*" (missing or zero in both Fields [46/7-2.0]).

[c] – exclusions the MRI dataset were determined by missing values in any of the MRI measurements OR hand grip strength at the imaging visit (Field [22407-2.0] "*Visceral adipose tissue volume (VAT)*"; Field [46-2.0] "*Hand grip strength (left)*"; Field [47-2.0] "*Hand grip strength (right)*").

[d] – anthropometric measurements were obtained from Fields [48-0/2.0] "*Waist circumference*", Field [49-0/2.0] "*Hip circumference*", Field [50-0/2.0] "*Standing height*" and Field [21002-0/2.0] "*Weight*".

[e] – excluded were participants with code 1 for Field [22019-0.0] "*Sex chromosome aneuploidy*" OR with a mismatch between Field [22001-0.0] "*Genetic sex*" and Field [34-0.0] "*Sex (self-reported)*".

[f] – Field [3140-0.0] "*Pregnant"*; Answer: 1 "Yes".

[g] – for ascertainment of prevalent and incident cancer cases see Supplementary Methods.

[h] – additional exclusions for the DXA VAT subset were determined by values in Field [23288-2.0] "*VAT (visceral adipose tissue) mass*": missing, zero or larger than Field [23245-2.0] "*Android Fat Mass*".

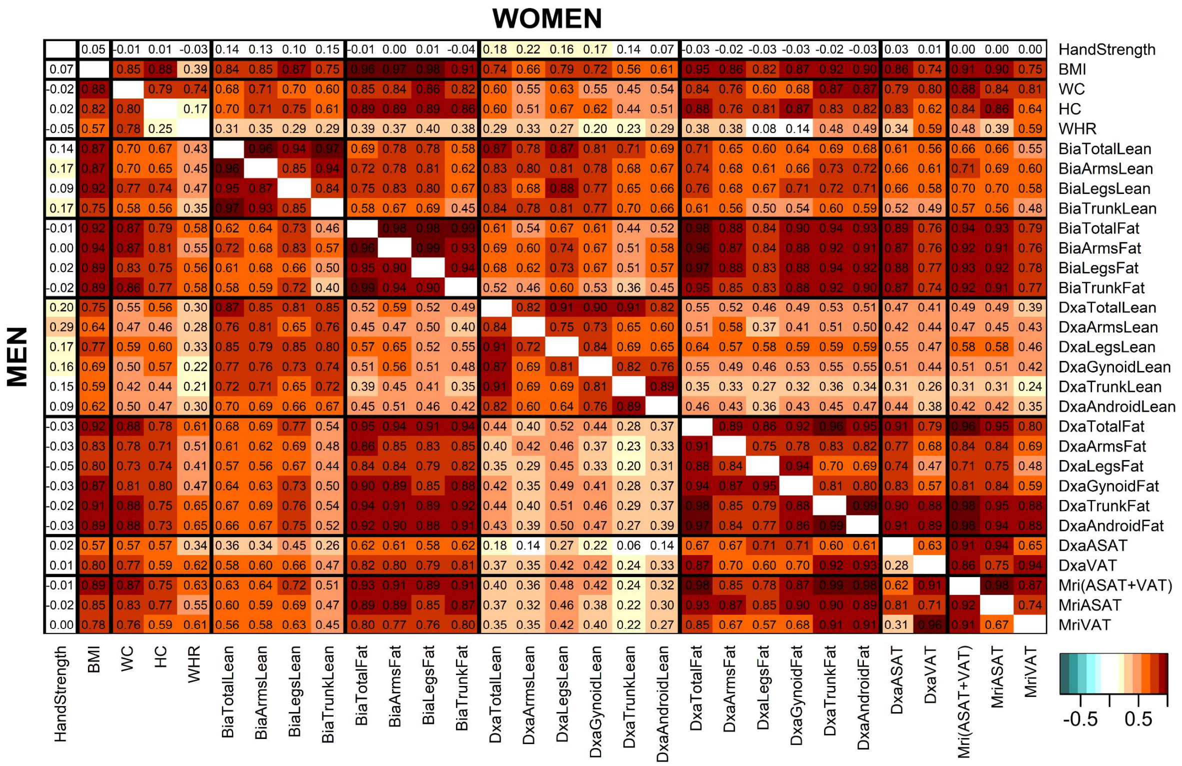

Supplementary Figure S2 Correlation between anthropometry and body composition among individuals with the same height

ASAT – abdominal subcutaneous adipose tissue; **Bia…** – bioelectrical impedance analysis (BIA) measurements; **BMI** – body mass index; **Dxa…** – dual-emission X-ray absorptiometry (DXA) measurements; **HC** – hip circumference; **Mri…** – magnetic resonance imaging (MRI) measurements; **VAT** – visceral adipose tissue; **WC** – waist circumference; **WHR** – waist-to-hip ratio. **Men** – bottom left half of panel. **Women** – top right half of panel. **Cells** – show partial Pearson correlation coefficients, with adjustment for age at enrolment, weight change during the last year preceding enrolment, smoking status, alcohol consumption, physical activity, Townsend deprivation index, region (for hand grip strength, DXA and BIA measurements, except DxaVAT and DxaASAT) and, for women, a combined variable with menopausal status and use of hormone replacement therapy for post-menopausal women. Anthropometry, hand grip strength, BIA and DXA measurements were obtained from the DXA dataset, MRI measurements from the overlap of the DXA and MRI datasets and DxaVAT and DxaASAT from the overlap of the DXA VAT and MRI datasets (see Supplementary Figure S1 for the definition of datasets). Measurements were converted to allometric indices with scaling only for height and then to sex-specific z-scores (value minus mean, divided by the standard deviation) (see scaling regression coefficients δ, formulas, means and standard deviations in Supplementary Table S1).

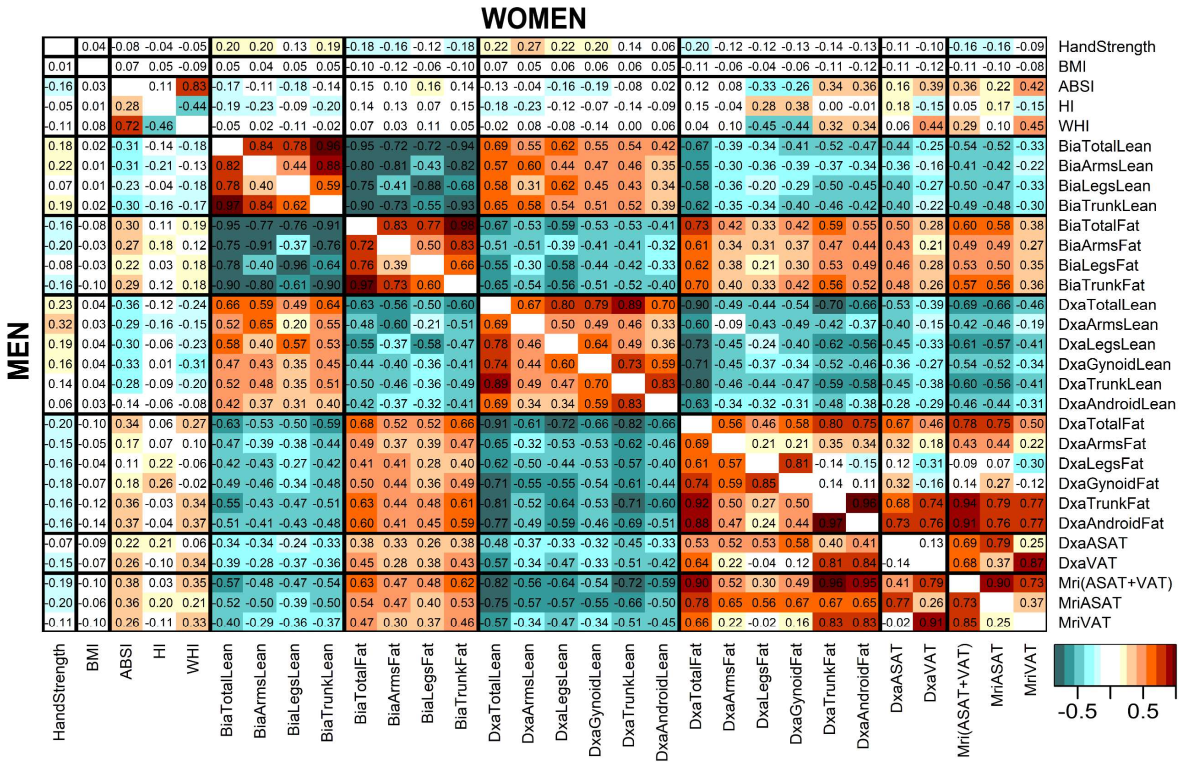

Supplementary Figure S3 Correlation between anthropometry and body composition among individuals with the same height and weight

ABSI – a body shape index; **ASAT** – abdominal subcutaneous adipose tissue; **Bia…** – bioelectrical impedance analysis (BIA) measurements; **BMI** – body mass index; **Dxa…** – dual-emission X-ray absorptiometry (DXA) measurements; **HI** – hip index; **Mri…** – magnetic resonance imaging (MRI) measurements; **VAT** – visceral adipose tissue; **WHI** – waist-to-hip index. **Men** – bottom left half of panel. **Women** – top right half of panel. **Cells** – show partial Pearson correlation coefficients, with adjustment for BMI, height, age at enrolment, weight change during the last year preceding enrolment, smoking status, alcohol consumption, physical activity, Townsend deprivation index, region (for hand grip strength, DXA and BIA measurements, except DxaVAT and DxaASAT) and, for women, a combined variable with menopausal status and use of hormone replacement therapy (for post-menopausal women). Anthropometry, hand grip strength, BIA and DXA measurements were obtained from the DXA dataset, MRI measurements from the overlap of the DXA and MRI datasets and DxaVAT and DxaASAT from the overlap of the DXA VAT and MRI datasets (see Supplementary Figure S1 for definition of datasets). Measurements were converted to allometric indices with scaling for weight and height and then to sex-specific z-scores (value minus mean, divided by the standard deviation) (see scaling regression coefficients β and γ, correspondingly, formulas, means and standard deviations in Supplementary Table S1) .

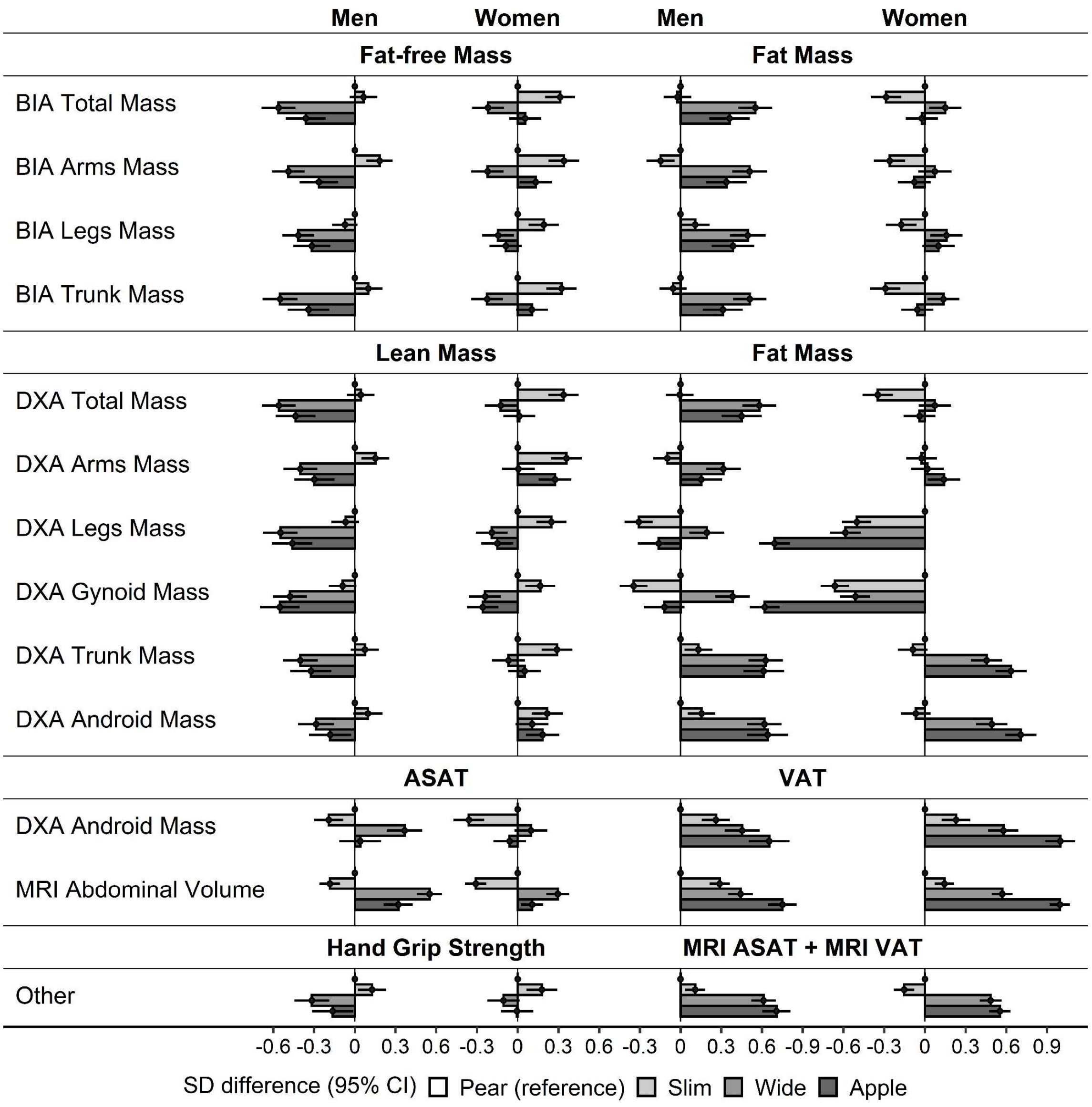

Supplementary Figure S4 Body composition profiles of body-shape phenotypes (DXA VAT subset)

ABSI – a body shape index (cut-offs: ≥80 in men; ≥73 in women); **Apple** – large-ABSI-small-HI; **ASAT** – abdominal subcutaneous adipose tissue; **BIA** – bioelectrical impedance analysis measurements; **CI** – confidence interval; **DXA** – dual-emission X-ray absorptiometry measurements; **HI** – hip index (cut-offs: ≥49 in men; ≥64 in women); **MRI** – magnetic resonance imaging measurements; **Pear** – small-ABSI-large-HI; **SD** – standard deviation; **Slim** – small-ABSI-small-HI; **VAT** – visceral adipose tissue; **Wide** – large-ABSI-large-HI; **SD difference (95% CI)** – derived from linear regression models with adjustment for age, self-reported weight change within the year preceding the visit, smoking status, alcohol consumption, physical activity, Townsend deprivation index, region (except for a single region for VAT, ASAT and MRI) and, in women, menopausal status and use of hormonal replacement therapy (see definition of covariates in

Supplementary Methods). Body-composition measurements were converted to allometric indices with scaling for height and weight (see scaling coefficients in Supplementary Table S1) and then to sex-specific z-scores (value minus mean, divided by the standard deviation). Note that DXA lean mass does not include bone mass, which is included in BIA fat-free mass. This is a sensitivity analysis for the restricted DXA VAT dataset, which contains measurements for regional DXA lean and fat mass provided by UK Biobank (see Supplementary Figure S1 for the definition of datasets).

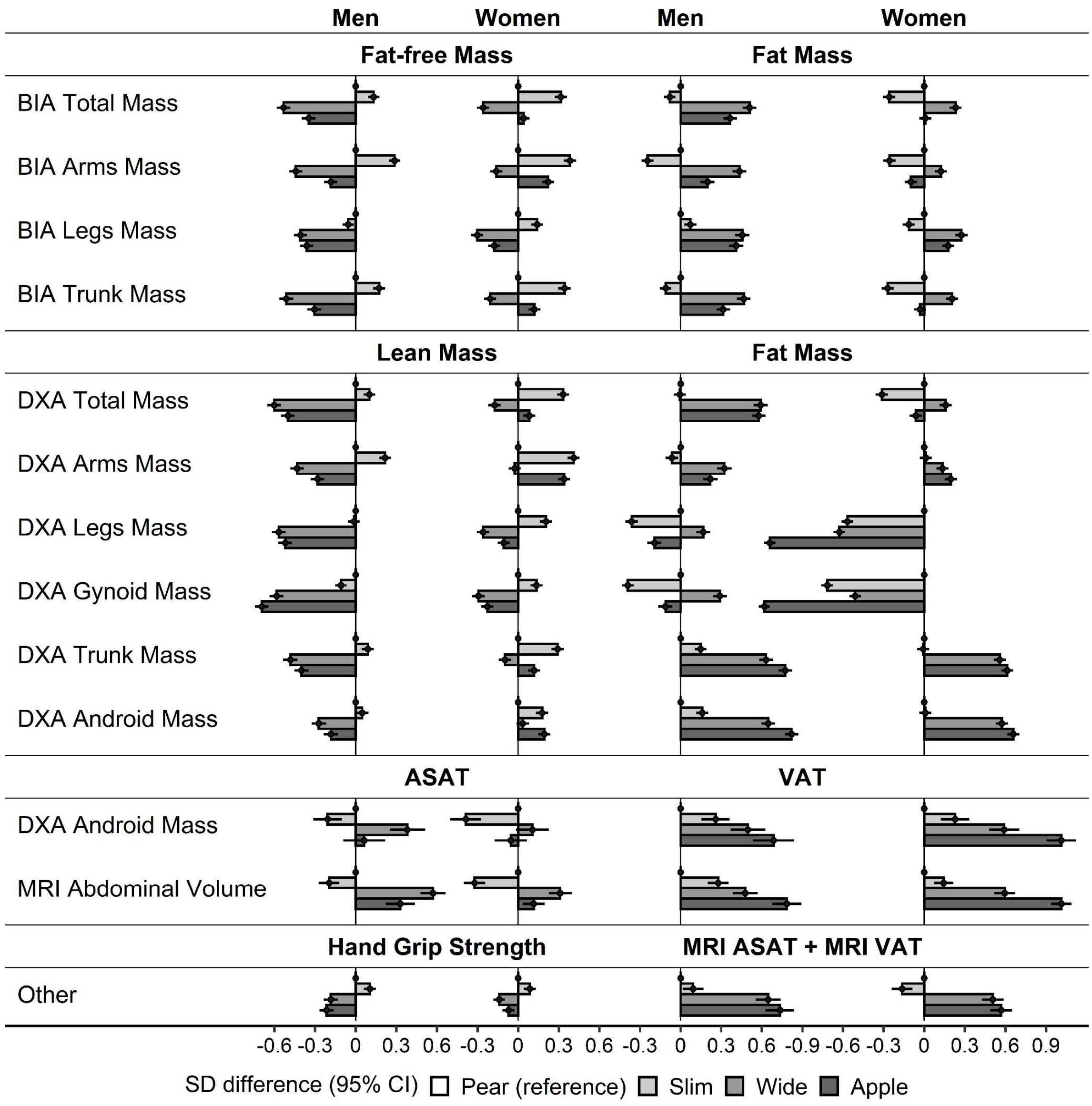

Supplementary Figure S5 Body composition profiles of body-shape phenotypes (minimally adjusted models)

ABSI – a body shape index (cut-offs: ≥80 in men; ≥73 in women); **Apple** – large-ABSI-small-HI; **ASAT** – abdominal subcutaneous adipose tissue; **BIA** – bioelectrical impedance analysis measurements; **CI** – confidence interval; **DXA** – dual-emission X-ray absorptiometry measurements; **HI** – hip index (cut-offs: ≥49 in men; ≥64 in women); **MRI** – magnetic resonance imaging measurements; **Pear** – small-ABSI-large-HI; **SD** – standard deviation; **Slim** – small-ABSI-small-HI; **VAT** – visceral adipose tissue; **Wide** – large-ABSI-large-HI; **SD difference (95% CI)** – derived from linear regression models with adjustment only for age and region (except for a single region for VAT, ASAT and MRI). Body-composition measurements were converted to allometric indices with scaling for weight and height (see scaling regression coefficients in Supplementary Table S1) and then to sex-specific z-scores (value minus mean, divided by the standard deviation).

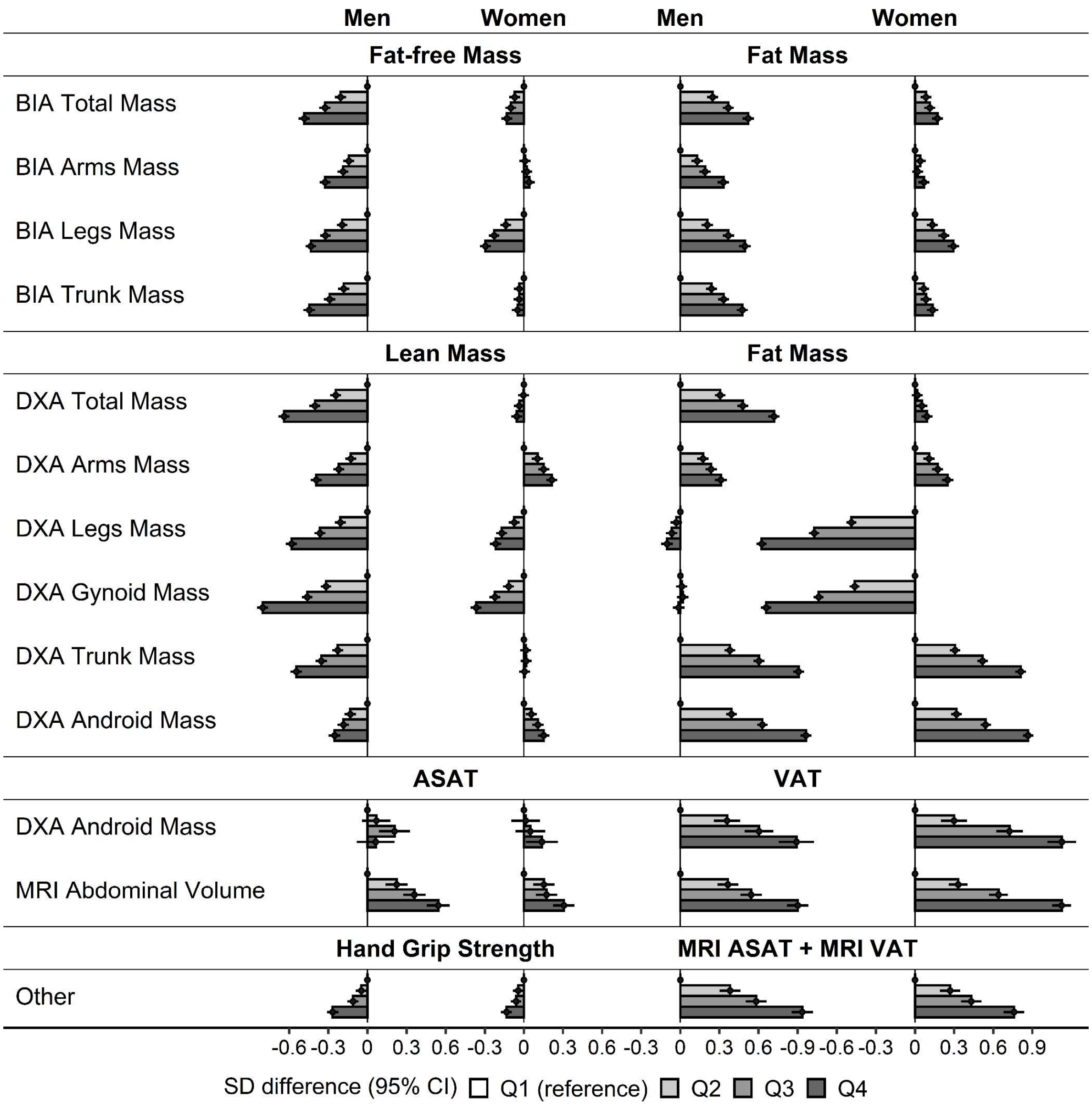

Supplementary Figure S6 Body composition profiles of waist-to-hip index quartiles

ASAT – abdominal subcutaneous adipose tissue; **BIA** – bioelectrical impedance analysis measurements; **CI** – confidence interval; **DXA** – dual-emission X-ray absorptiometry measurements; **MRI** – magnetic resonance imaging measurements; **Q1-4** – WHI quartiles (quartile boundaries were: 3.946, 4.088, 4.235 for men and 3.436, 3.608, 3.797 for women in the DXA dataset; 3.902, 4.022, 4.150 for men and 3.420, 3.578, 3.747 for women in the DXA VAT subset; 3.905, 4.027, 4.156 for men and 3.404, 3.569, 3.737 for women in the MRI dataset, see Supplementary Figure S1 for definition of datasets); **SD** – standard deviation; **VAT** – visceral adipose tissue; **WHI** – waist-to-hip index.

SD difference (95% CI) – derived from linear regression models with adjustment for age, self-reported weight change within the year preceding the visit, smoking status, alcohol consumption, physical activity, Townsend deprivation index, region (except for a single region for VAT, ASAT and MRI) and, in women, menopausal status and use of hormonal replacement therapy (see

definition of covariates in Supplementary Methods). Body-composition measurements were converted to allometric indices with scaling for height and weight (see scaling regression coefficients in Supplementary Table S1) and then to sex-specific z-scores (value minus mean, divided by the standard deviation). Note that DXA lean mass does not include bone mass, which is included in BIA fat-free mass. See Supplementary Methods for the calculation of DXA lean and fat mass.

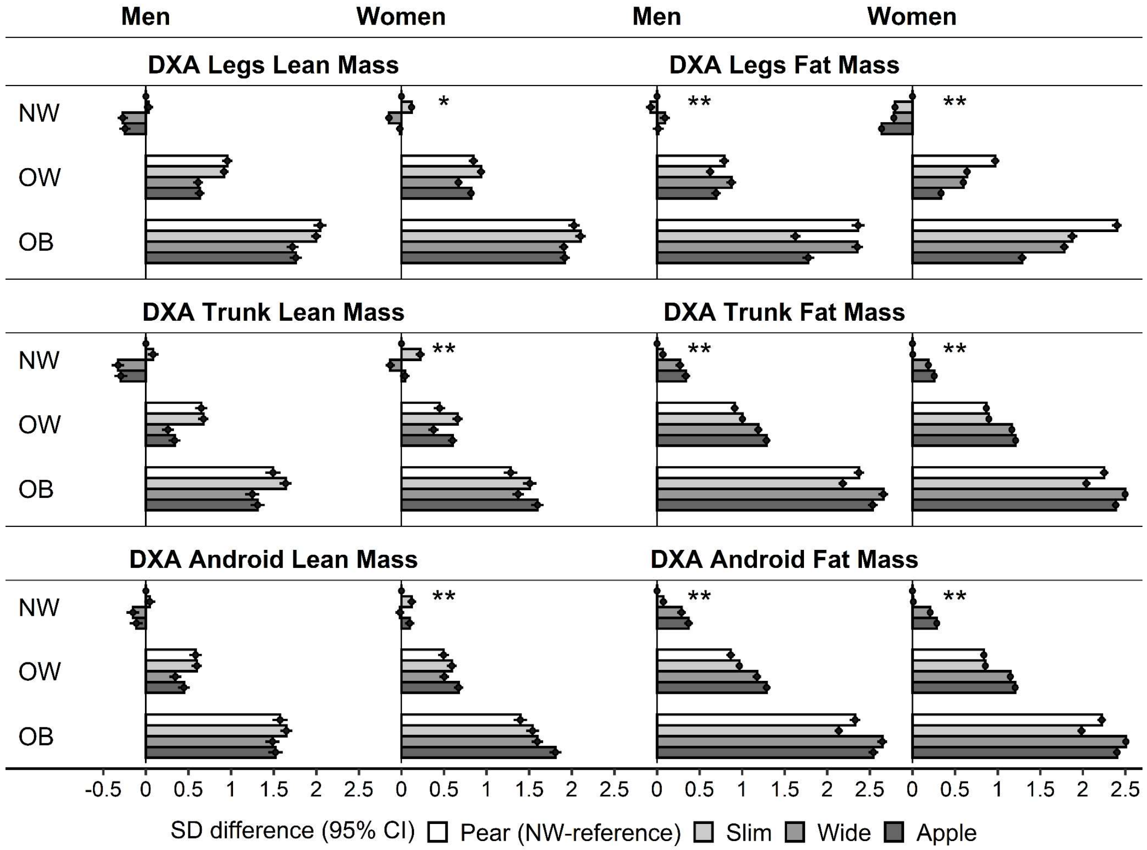

Supplementary Figure S7 Body composition profiles of categories by body-shape phenotype and BMI category (extension)

ABSI – a body shape index (cut-offs: ≥80 in men; ≥73 in women); **Apple** – large-ABSI-small-HI; **ASAT** – abdominal subcutaneous adipose tissue; **BMI** – body mass index; **CI** – confidence interval; **DXA** – dual-emission X-ray absorptiometry measurements; **HI** – hip index (cut-offs: ≥49 in men; ≥64 in women); **MRI** – magnetic resonance imaging measurements; **NW** – normal weight BMI≥18.5 to <25.0 kg/m^2; **OB** – obese BMI≥30.0 to <45 kg/m^2; **OW** – overweight BMI≥25.0 to <30.0 kg/m^2; **Pear** – small-ABSI-large-HI; **SD** – standard deviation; **Slim** – small-ABSI-small-HI; **VAT** – visceral adipose tissue; **Wide** – large-ABSI-large-HI.

SD difference (95% CI) – derived from linear regression models with adjustment for age, self-reported weight change within the year preceding the visit, smoking status, alcohol consumption, physical activity, Townsend deprivation index, region (except for a single region for VAT, ASAT and MRI) and, in women, menopausal status and use of hormonal replacement therapy (see definition of covariates in Supplementary Methods and numerical values in Supplementary Table S6). Body-composition measurements were converted to allometric indices with scaling for height (see scaling regression coefficients in Supplementary Table S1) and then to sex-specific z-scores (value minus mean, divided by the standard deviation).

* p<0.05; ** p<0.001 p-values from a likelihood ratio test comparing a model with separate ABSI-by-HI and BMI categorical variables with a model with an BMI-by-ABSI-by-HI cross-classification.

A. Colon cancer (minimally adjusted models)

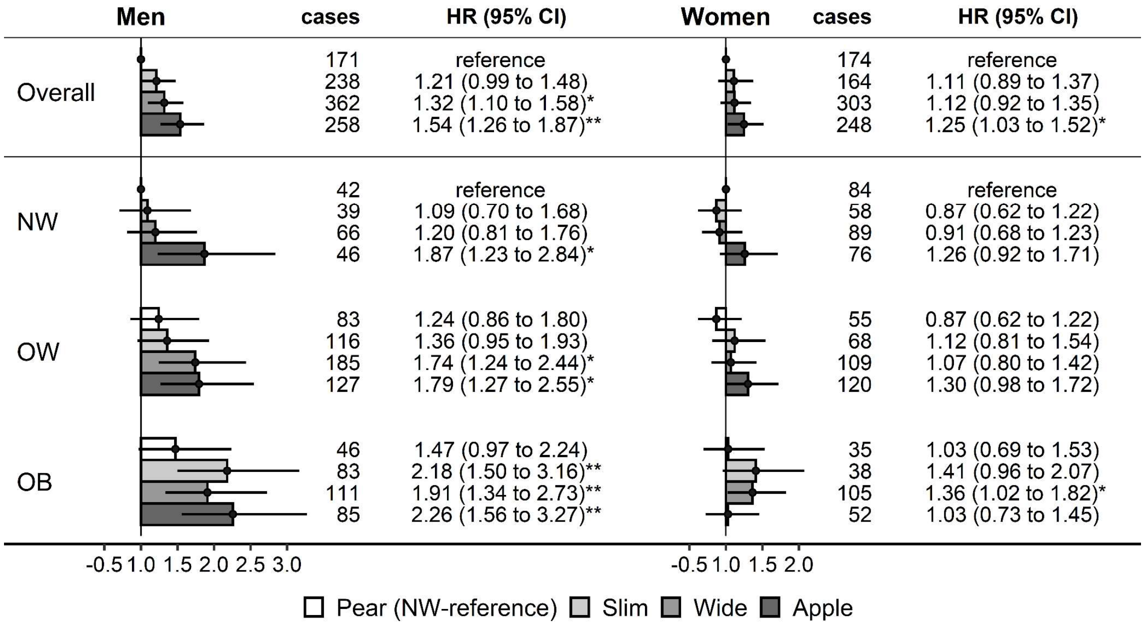

	Men cases	Men HR (95% CI)	Women cases	Women HR (95% CI)
Overall	171	reference	174	reference
	238	1.21 (0.99 to 1.48)	164	1.11 (0.89 to 1.37)
	362	1.32 (1.10 to 1.58)*	303	1.12 (0.92 to 1.35)
	258	1.54 (1.26 to 1.87)**	248	1.25 (1.03 to 1.52)*
NW	42	reference	84	reference
	39	1.09 (0.70 to 1.68)	58	0.87 (0.62 to 1.22)
	66	1.20 (0.81 to 1.76)	89	0.91 (0.68 to 1.23)
	46	1.87 (1.23 to 2.84)*	76	1.26 (0.92 to 1.71)
OW	83	1.24 (0.86 to 1.80)	55	0.87 (0.62 to 1.22)
	116	1.36 (0.95 to 1.93)	68	1.12 (0.81 to 1.54)
	185	1.74 (1.24 to 2.44)*	109	1.07 (0.80 to 1.42)
	127	1.79 (1.27 to 2.55)*	120	1.30 (0.98 to 1.72)
OB	46	1.47 (0.97 to 2.24)	35	1.03 (0.69 to 1.53)
	83	2.18 (1.50 to 3.16)**	38	1.41 (0.96 to 2.07)
	111	1.91 (1.34 to 2.73)**	105	1.36 (1.02 to 1.82)*
	85	2.26 (1.56 to 3.27)**	52	1.03 (0.73 to 1.45)

B. Colon cancer (excluding participants with less than two years of follow-up)

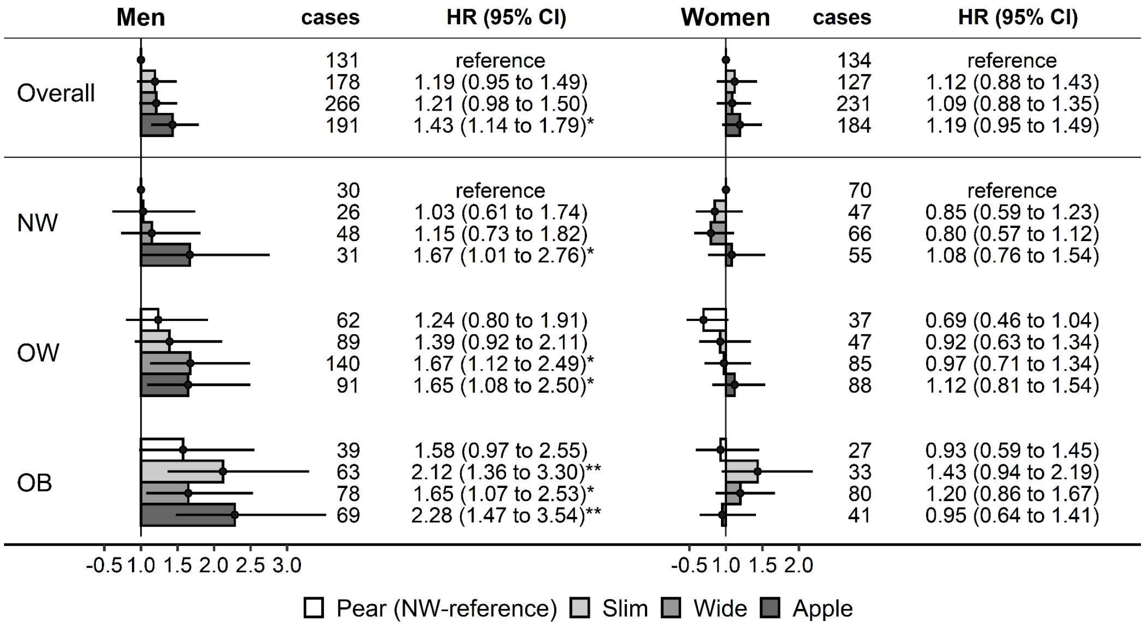

	Men cases	Men HR (95% CI)	Women cases	Women HR (95% CI)
Overall	131	reference	134	reference
	178	1.19 (0.95 to 1.49)	127	1.12 (0.88 to 1.43)
	266	1.21 (0.98 to 1.50)	231	1.09 (0.88 to 1.35)
	191	1.43 (1.14 to 1.79)*	184	1.19 (0.95 to 1.49)
NW	30	reference	70	reference
	26	1.03 (0.61 to 1.74)	47	0.85 (0.59 to 1.23)
	48	1.15 (0.73 to 1.82)	66	0.80 (0.57 to 1.12)
	31	1.67 (1.01 to 2.76)*	55	1.08 (0.76 to 1.54)
OW	62	1.24 (0.80 to 1.91)	37	0.69 (0.46 to 1.04)
	89	1.39 (0.92 to 2.11)	47	0.92 (0.63 to 1.34)
	140	1.67 (1.12 to 2.49)*	85	0.97 (0.71 to 1.34)
	91	1.65 (1.08 to 2.50)*	88	1.12 (0.81 to 1.54)
OB	39	1.58 (0.97 to 2.55)	27	0.93 (0.59 to 1.45)
	63	2.12 (1.36 to 3.30)**	33	1.43 (0.94 to 2.19)
	78	1.65 (1.07 to 2.53)*	80	1.20 (0.86 to 1.67)
	69	2.28 (1.47 to 3.54)**	41	0.95 (0.64 to 1.41)

Supplementary Figure S8 Body-shape phenotypes in relation to colon cancer risk (sensitivity analyses)

ABSI – a body shape index (cut-offs: ≥80 in men; ≥73 in women); **Apple** – large-ABSI-small-HI; **BMI** – body mass index; **CI** – confidence interval; **HI** – hip index (cut-offs: ≥49 in men; ≥64 in women); **HR** – hazard ratio; **NW** – normal weight BMI≥18.5 to <25.0 kg/m^2; **OB** – obese BMI≥30.0

to <45 kg/m^2; **OW** – overweight BMI≥25.0 to <30.0 kg/m^2; **Pear** – small-ABSI-large-HI; **Slim** – small-ABSI-small-HI; **Wide** – large-ABSI-large-HI.

HR (95% CI) – derived from cox proportional hazards models: (A) stratified by age and region and adjusted for BMI (for overall) and height; (B) additionally adjusted for self-reported weight change within the year preceding the visit, smoking status, alcohol consumption, physical activity, Townsend deprivation index, diet (consumption of fresh fruit and vegetables, red meat, processed meat, fibre), use of non-steroidal anti-inflammatory drugs, family history of cancer and, in women, menopausal status, use of oral contraceptives and hormonal replacement therapy and age at last live birth (see definition of covariates in Supplementary Methods).

References

The numbers of the cited references correspond to the main document.

5. Krakauer NY, Krakauer JC. An Anthropometric Risk Index Based on Combining Height, Weight, Waist, and Hip Measurements. *J Obes* 2016 doi:10.1155/2016/8094275.

7. Christakoudi S, Tsilidis KK; Evangelou E, at al. A Body Shape Index (ABSI), hip index and risk of cancer in the UK Biobank cohort. *Cancer Med* 2021; doi: 10.1002/CAM4.4097

13. Stults-Kolehmainen MA, Stanforth PR, Bartholomew JB, et al. DXA estimates of fat in abdominal, trunk and hip regions varies by ethnicity in men. *Nutr Diabetes* 2013;3:e64. doi: 10.1038/nutd.2013.5

14. West J, Dahlqvist Leinhard O, Romu T, et al. Feasibility of MR-Based Body Composition Analysis in Large Scale Population Studies. *PLoS One* 2016; 11(9). doi: 10.1371/journal.pone.0163332

17. Bradbury KE, Young HJ, Guo W, et al. Dietary assessment in UK Biobank: an evaluation of the performance of the touchscreen dietary questionnaire. *J Nutr Sci* 2018; 7 doi: 10.1017/jns.2017.66

28. Kaul S, Rothney MP, Peters DM, et al. Dual-energy X-ray absorptiometry for quantification of visceral fat. *Obesity (Silver Spring)* 2012;20(6):1313-8. doi: 10.1038/oby.2011.393
